# Spike 1 trimer, a nanoparticle vaccine against porcine epidemic diarrhea virus induces protective immunity challenge in piglets

**DOI:** 10.3389/fmicb.2024.1386136

**Published:** 2024-04-08

**Authors:** Linjie Li, Shuanghui Yin, Jingjing Zhou, Liping Zhang, Zhidong Teng, Lu Qiao, Yunhang Wang, Jiaxi Yu, Haoyue Zang, Yaozhong Ding, Xinsheng Liu, Shiqi Sun, Huichen Guo

**Affiliations:** ^1^State Key Laboratory for Animal Disease Control and Prevention, College of Veterinary Medicine, Lanzhou University, Lanzhou Veterinary Research Institute, Chinese Academy of Agricultural Sciences, Lanzhou, China; ^2^Gansu Province Research Center for Basic Disciplines of Pathogen Biology, Lanzhou, China

**Keywords:** PEDV, S1-Trimer, nanovaccines, mice, sows, piglets

## Abstract

Porcine epidemic diarrhea virus (PEDV) is considered the cause for porcine epidemic diarrhea (PED) outbreaks and hefty losses in pig farming. However, no effective commercial vaccines against PEDV mutant strains are available nowadays. Here, we constructed three native-like trimeric candidate nanovaccines, i.e., spike 1 trimer (S1-Trimer), collagenase equivalent domain trimer (COE-Trimer), and receptor-binding domain trimer (RBD-Trimer) for PEDV based on Trimer-Tag technology. And evaluated its physical properties and immune efficacy. The result showed that the candidate nanovaccines were safe for mice and pregnant sows, and no animal death or miscarriage occurred in our study. S1-Trimer showed stable physical properties, high cell uptake rate and receptor affinity. In the mouse, sow and piglet models, immunization of S1-Trimer induced high-level of humoral immunity containing PEDV-specific IgG and IgA. S1-Trimer-driven mucosal IgA responses and systemic IgG responses exhibited high titers of virus neutralizing antibodies (NAbs) *in vitro*. S1-Trimer induced Th1-biased cellular immune responses in mice. Moreover, the piglets from the S1-Trimer and inactivated vaccine groups displayed significantly fewer microscopic lesions in the intestinal tissue, with only one and two piglets showing mild diarrhea. The viral load in feces and intestines from the S1-Trimer and inactivated vaccine groups were significantly lower than those of the PBS group. For the first time, our data demonstrated the protective efficacy of Trimer-Tag-based nanovaccines used for PEDV. The S1-Trimer developed in this study was a competitive vaccine candidate, and Trimer-Tag may be an important platform for the rapid production of safe and effective subunit vaccines in the future.

## 1 Introduction

Porcine epidemic diarrhea (PED) is an acute and highly contagious enteric disease characterized by severe dehydration and watery diarrhea (Debouck and Pensaert, 1980). This disease was first reported in the United Kingdom in 1971 (Wood, 1977). The pathogen of PED is the porcine epidemic diarrhea virus (PEDV), which can infect pigs of all ages, with a high morbidity rate in neonatal piglets. In 2010, China experienced a PED outbreak (Sun et al., 2012). which resulted in considerable economic losses. Inactivated or attenuated CV777 and AJ1102 strains were the main vaccines used to control PED outbreaks in China. However, PEDV vaccines that are based on classical strains, however, could not prevent the spread of PEDV variants in China. Therefore, more effective vaccines must be developed. Self-assembly nanoparticles (NPs) in vaccines have been successfully developed. Through the use of NPs as multimerization platforms, viral proteins were selected and integrated to a noninfectious and highly immunogenic scaffold to mimic the three-dimensional structure of native viral particles (Jennings and Bachmann, 2008; Shirbaghaee and Bolhassani, 2016).

PEDV is a single stranded positive RNA virus with an envelope (Kocherhans et al., 2001). The genome is approximately 28 kb, and contains two replicase polyproteins, i.e., open reading frame (ORF) 1a and ORF1b, and four structural proteins, i.e., spike (S), envelope (E), membrane (M), and nucleocapsid (N) (Huang et al., 2013). The S protein was considered a good candidate for subunit vaccines (Kirchdoerfer et al., 2021), because it could mediate the production of neutralizing antibodies. Neutralizing epitope regions have been already gradually identified, cell attachment domains were key targets of neutralizing antibodies (Li et al., 2017), S1^A^ (aa 435–485) (Chang et al., 2019), COE (aa 499–638) (Chang et al., 2002), the SS2 (aa 744–759) and SS6 (aa 756–771) (Okda et al., 2017), and the C-terminal epitope 2C10 (aa 1,368–1,374, 1,371–1,377) (Cruz et al., 2006, 2008; Okda et al., 2017). The S protein consists of two domains: S1 (aa 1–735) and S2 (736—the last aa). The S1 domain could interact with the cellular receptor (Li, 2012). A study on the intramuscular injection of S1 protein in late-term pregnant sows indicated that S1 protein could provide effective protection against PEDV challenge in neonatal piglets (Oh et al., 2014). S2 includes a fusion peptide (FP) that mediates the fusion of cellular membranes and viruses. The FP core (IEDLLF) is identical to all coronaviruses of the four genera. Previously studies suggested that immunization with vaccines based on the core FP sequence (IEDLLF) provided protection for pigs against PEDV and SARS-CoV-2 (Maeda et al., 2021). FPs were also verified as an attractive candidate vaccine against coronavirus (Walls et al., 2020). The PEDV S protein was demonstrated as a natural homotrimer (Colman and Lawrence, 2003; Wrapp and McLellan, 2019). It is important for vaccine efficacy that maintaining viral antigens natural trimeric conformation. A recent study showed that asymptomatic HIV-positive carriers produced neutralizing antibodies that could be recognized by the native trimeric gp140 but not by monomeric gp120 (de Taeye et al., 2016). These problems were addressed by using human C-propeptide of α1(I) collagen (COLIA1) as Trimer-Tag to produce NPs that could protect animal models against COVID-19 and antitumor (Liu et al., 2017; Liang et al., 2021).

Here, we described the use of a platform technology called Trimer-Tag (Liu et al., 2017). With expressed by yeast to produce rapidly a native-like fusion form of trimeric S1 protein subunit and FP antigen derived from the wild-type viral sequence as a PEDV vaccine candidate. And the fusion of human C-propeptide of α1(I) collagen (Trimer-Tag) to the C-terminus of PEDV antigen formed homotrimer through disulfide bonding, maintained the natural conformation of S protein. The results demonstrated that S1-Trimer induced high levels of humoral immune response and Th1-biased cellular immune responses in mice. After immunization of sows with S1-Trimer, passive immunity effectively protected the piglets against the challenge of PEDV.

## 2 Materials and methods

### 2.1 Cell lines, viruses, and animals

Macrophage RAW264.7 and Vero-CCL81 were cultured in Dulbecco’s modified Eagle medium (DMEM; Gibco, United States) containing 10% fetal bovine serum (FBS; Gibco, United States) and 100 U/mL penicillin-streptomycin solution at 37°C with 5% CO_2_. Bone marrow-derived dendritic cells (BMDCs) were isolated from the femurs and tibias of six-week-old C57BL/6 mice and cultured in the Roswell Park Memorial Institute (RPMI; Gibco, United States) 1,640 supplemented with 15% heat-inactivated FBS, 100 U/mL penicillin, 100 μg/mL streptomycin, and 55 μM β-mercaptoethanol. PichiaPink strain 1 (Invitrogen) were cultured in YPD medium.

The PEDV GS2022 strain (GenBank: OR608353) used in this study was isolated from pigs with severe diarrhea and passed through the Vero cell. GS2022 strain belongs to G2d (Li et al., 2024).

Female BALB/c and C57BL/6 mice (six-week-old) in specific pathogen-free grade were purchased from and housed in the Laboratory Animal Centre, Lanzhou Veterinary Research Institute. Pregnant sows were purchased from the Lei Xin pig farm (Lanzhou Gansu).

All animal experiments were approved and supervised by the Animal Ethics Committee of Lanzhou Veterinary Research Institute, Chinese Academy of Agriculture Sciences (Approval number: LVRIAEC-2023-050; LVRIAEC-2023-053), and all animal procedures were performed in accordance with the Animal Ethics Procedures and Guidelines of the People’s Republic of China.

### 2.2 Gene synthesis and plasmid construction

The PEDV S1-Trimer fusion protein was produced by gene-synthesizing a cDNA encoding the S1 subunit and FP of wild-type PEDV S protein (amino acid residues 1–789 and 891–908) using yeast codon optimized by GenScript. The S1 subunit and FP were linked through a linker (GS). The PEDV RBD construct (amino acid residues 499–638) and COE construct (499–638, 748–755, 764–771, 891–908 and 1,368–1,374) for preparation of the RBD-Trimer and COE-Trimer fusion protein were basically the same as above. The cDNA was subcloned into pPink-HC expression vector (Thermo Fisher Scientific) at EcoRI and KpnI sites to allow in-frame fusion of the soluble RBD, COE and S1 protein to Trimer-Tag (amino acid residue 1,156–1,406 from human type I (α) collagen) as described previously (Liu et al., 2017; Liang et al., 2021).

Amino acid sequences of PEDV AJ1102 S protein (GenBank: JX188454) and human type I (α) collagen (GenBank: NP_000079.2) were used in this study.

### 2.3 Protein expression and purification

The recombinant protein was expressed in accordance with the manufacturer’s instructions (Thermo Fisher Scientific) and was purified with nickel-nitrilotriacetic acid (Ni-NTA) resin (Roche). Pierce bicinchoninic acid (BCA) assay kit (Thermo Fisher Scientific), western blots, and SDS-PAGE were performed to detect the concentration, expression, and purification of the recombinant protein. The antibody used for Western blotting was rabbit anti-6-His Tag Antibody HRP conjugates (Bethyl, United States; 1:5000).

### 2.4 Generation of NPs

The protein was purified and transferred to a 10 kDa molecular weight cutoff cellulose ester dialysis tubing and dialyzed overnight at 4°C to exchange the buffer and assemble NPs. BCA assay kit, dynamic light scattering (DLS), and transmission electron microscopy (TEM; HITACHI, Japan) were performed to detect the concentration and characterize the NPs. Then COLIA1-Trimer, RBD-Trimer, COE-Trimer, and S1-Trimer were mixed 1:1.2 (v/v) with an IAS 201 adjuvant (SEPPIC, France), respectively.

### 2.5 Prediction 3D structure of NPs

The structures of the PEDV CV777 strain (PDB ID: 6U7K) (Wrapp and McLellan, 2019) and Pintung 52 strain (PDB ID:7W6M) (Huang et al., 2022) were used as templates to compare with the RBD-Trimer, COE-Trimer, and S1-Trimer. The SiFold server^1^ and the SWISS-MODEL server^2^ were exploited for protein simulation, whereas PyMoL (The PyMOL Molecular Graphics System, Version 2.6 Schrodinger, LLC) was used to display 3D structures and surfaces.

### 2.6 Thermostability and long-term storage stability evaluation

The thermostability and aggregation of NPs were measured by the samples of NPs and monomers which were diluted to a concentration of 500 μg/mL. Afterward, the NPs and monomers were incubated at 25, 35, 45, 55, 65°C for 1 h and then cooled to 4°C. The supernatant was performed via a BCA assay kit and DLS analysis. The sample held at 25°C was defined as 100% soluble. All samples were run in triplicate.

NPs were stored at room temperature for 6 weeks to evaluate the stability of long-term storage. At the indicated time points, the samples were observed via TEM for NP integrity analysis. The number of NPs was counted using Image J software (Bethesda, MD, United States).

### 2.7 Cellular uptake and cytotoxicity

BMDCs, RAW264.7 and Vero cells were seeded in six-well dish and treated with 10 μg of RBD-Trimer, COE-Trimer, S1-Trimer, RBD-Monomer, COE-Monomer or S1-Monomer for 6 h. Lyso-Tracker Red (Solarbio, China; 1:15000), FITC-Monoclonal Mouse Anti-His antibody (OKA, China; 1:1000), and 4′,6-diamidino-2-phenylindole (DAPI; Sigma, United States; 1:2000) were used to label lysosomes, antigens, and nuclei. An inverted fluorescence microscope was used to take fluorescence images (Olympus, Japan). CellTiter 96^®^ AQueous One Solution Cell Proliferation Assay (MTS) (Promega, United States) was performed to detect cell viability. Optical density (OD) was measured at 490 nm.

### 2.8 Immunization of BALB/c mice and piglets

A total of 50 six-week-old female BALB/c mice were randomly divided into five groups. Then, the mice were immunized with PBS (pH = 7.4), COLIA1-Trimer (25 μg), RBD-Trimer (25 μg), COE-Trimer (25 μg), and S1-Trimer (25 μg) via intramuscular injection at zeroth and second week. Orbital blood was collected weekly after immunization. Sera and intestine samples were collected and stored at −20°C for further analyses. Mice were sacrificed at the fourth week.

A total of 15 pregnant sows (35 days before delivery) were randomly divided into three groups, and each group had five sows. All sow pathogen and serum tests were PEDV negative. The day of production was regarded as day 0. The sows were, respectively, administered S1-Trimer (100 ug), inactivated PEDV AJ1102 strain (2 mL) containing 10^7.5^ TCID_50_/mL, and PBS (2 mL) intramuscularly at −35 days and −20 days antepartum. Clinical symptoms, including anorexia, abortion, and necrosis at the inoculation site, were observed after every vaccination. After production, five five-day-old piglets were randomly selected from each sow, fed with fresh milk (Jingzhun, China) three times a day, and challenged with the PEDV GS2022 strain (3 × 10^4.5^ TCID_50_/mL).

After immunization, serum was collected from the sows at −35, −15, 5, 15 and 25 days to detect titers of PEDV-specific IgG and NAbs. Milk was collected from the sows at 0, 5, and 15 days to detect titers of PEDV-specific IgA and NAbs. Saliva was collected from the sows at −35, −5 and 25 days and detected titers of PEDV-specific IgA and NAbs.

After being challenged orally with PEDV, the piglets were euthanized and dissected on the seventh days. Clinical signs were monitored every day. Diarrhea scores ranges from 1 to 3, with 1 being normal, 2 being pasty feces, and 3 being liquid diarrhea with no solid content (Lu et al., 2020). Feces were collected every day and detected for virus load via real-time quantitative PCR (RT-qPCR). The specific primers target to PEDV N gene are shown in Table 1. Sera were collected and tested for NAbs, and PEDV-specific IgG and IgA. Small intestines were collected and detected for neutralizing antibodies, PEDV-specific IgA, and viral load.

**Table 1:** The primers information used in RT-qPCR.

Primer	Sequence
PEDV-N-F	5′-AGATCGCCAGTTTAGCACCA-3′
PEDV-N-R	5′-GGCAAACCCACATCATCGT-3′
Mouse-IFN-γ F	5′-TCAGCTGATCCTTTGGACCC-3′
Mouse-IFN-γ R	5′-CTCAGAGCTAGGCCGCAGG-3′
Mouse-IL-4 F	5′-TCTTGATAAACTTAATTGTCTCTCGTCAC-3′
Mouse-IL-4 R	5′-GCAGGATGACAACTAGCTGGG-3′
Mouse-β-actin F	5′-GCTGTCCCTGTATGCCTCT-3′
Mouse-β-actin R	5′-TTGATGTCACGCACGATTT-3′
Pig-IFN-γ-F	5′-AGAATTGGAAAGAGGAGAGTGACAA-3′
Pig-IFN-γ-R	5′-TGAATGGCCTGGTTATCTTTGA-3′
Pig-IL-4-F	5′-CTCCCAACTGATCCCAACCC-3′
Pig-IL-4-R	5′-TGCACGAGTTCTTTCTCGCT-3′
Pig-GAPDH-F	5′-ACATGGCCTCCAAGGAGTAAGA-3′
Pig-GAPDH-R	5′-GATCGAGTTGGGGCTGTGACT-3′

To evaluate the safety of vaccines, body weight of mouse was weighted weekly, body weight and temperature of piglet was measured daily.

### 2.9 Enzyme-linked immunosorbent assay

PEDV-specific IgA/IgG titers in the milk, saliva, intestine samples and serum collected from immunized animals were determined via enzyme-linked immunosorbent assay (ELISA). PEDV-specific IgA titers were evaluated using a commercial mice PEDV antibody IgA ELISA kit (Sbjbio, China) and a porcine PEDV IgA ELISA kit (Mlbio, China) according to the manufacturer’s instruction.

The PEDV-specific IgG titers were detected by coating 96-well plates (Corning) with RBD, COE or S1 (500 ng/mL, 50 μL/well) at 4°C overnight. Goat antimouse IgG HRP (Sigma, United States; 1:1000) or rabbit anti pig IgG HRP (Thermo, United States; 1:1000) were used as the secondary antibody. The OD was measured at 450 nm.

### 2.10 Neutralization assay

Sera, milk, saliva, and intestine samples were inactivated at 56°C for 30 min. Heat-inactivated samples were serially diluted in two-fold dilutions to 1:512 and mixed with an equal volume of solution containing 100 TCID50 PEDV for 1 h at 37°C. Mixtures were transferred to Vero-CCL81 cells and cultured in 96-well plates for 72 h at 37°C. Cytopathic effect (CPE) was observed and recorded. The reciprocal of the highest dilution that caused complete neutralization was considered as the neutralization titer (NT).

### 2.11 Detection of cytokines in mice and sows

In accordance with the manufacturer’s instruction, the levels of IFN-γ and IL-4 in mice serum were detected by a commercial mice IFN-γ and IL-4 ELISA kit (R&D Systems, United States). The levels of IFN-γ and IL-4 in mice splenocytes were detected by RT-qPCR.

Peripheral blood mononuclear cells (PBMCs) were isolated from the blood of sows and cultured in 12-well plates at 37°C with or without of concanavalin A (Con A). The mRNA expression of IFN-γ and IL-4 in PBMCs were analyzed by RT-qPCR.

The specific primers of IFN-γ and IL-4 are shown in Table 1.

### 2.12 Proliferation of mouse splenic lymphocyte assay

Splenic lymphocytes were isolated and cultured in 96-well plates at 37°C with 5% CO_2_. After stimulation with or without Con A, the proliferation of splenic lymphocyte was detected by MTS according to the manufacturer’s instructions. The OD was measured at 490 nm.

### 2.13 Histopathology and immunohistochemistry

Jejunum was collected, then villous length (V) and crypt depth (C) were measured at 10 different sites in each sample, and the average of V:C was calculated. Intestinal lesion scores ranged from 1 to 4, with 1 being normal (V:C ≥ 3), 2 being mild villus atrophy (2 ≤ V:C ≤ 3), and 3 being moderate villus (1 ≤ V:C ≤ 2), 4 being severe villus atrophy, (V:C ≤ 1) or without villi or crypt. PEDV N-specific monoclonal antibody was used for Histopathology and immunohistochemistry (IHC).

### 2.14 Statistical analysis

Excel and GraphPad Prism 8 software (GraphPad Software, Inc., La Jolla, CA, United States) were used in this study to perform statistical analysis. Experimental data were analyzed using one-way ANOVA or student’s *t*-test. Data are shown as mean ± SD, and differences were defined as statistically significant at “∗” *p* < 0.05, “∗∗” *p* < 0.01, “∗∗∗” *p* < 0.001, and “∗∗∗∗” *p* < 0.0001.

## 3 Results

### 3.1 High-level expression and successful purification

**Figure 1:**
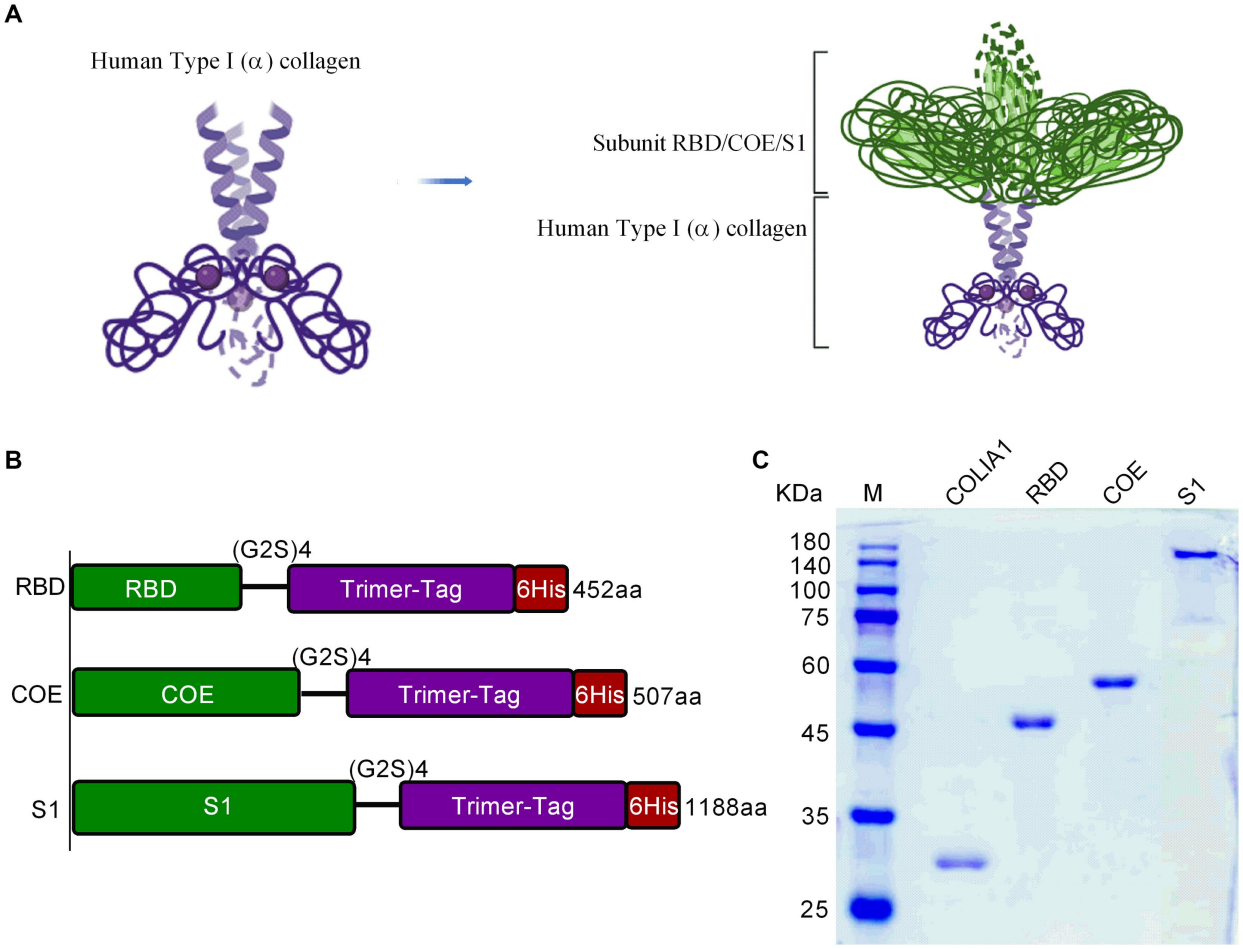
The structural characteristics and purification of NPs. **(A)** Schematic of NPs structure. **(B)** Diagram of recombinant protein gene structure. Each gene was showed in accordance with the displayed palette of **(A)**. **(C)** SDS-PAGE of purified COLIA1, RBD, COE and S1.

In the present study, we focused on the PEDV S-glycoprotein to design RBD-Trimer, COE-Trimer, and S1-Trimer vaccines based on Trimer-Tag technology (Liu et al., 2017; Liang et al., 2021). As previously described, COLIA1 could self-assemble into trimers via disulfide bonds (Sharma et al., 2017) (Supplementary Figure S1). Therefore, the addition of COLIA1 at the C-terminus of PEDV RBD, COE, and S1 resulted in the formation of a homotrimer linked by disulfide bonds (Figure 1A). As shown in Figure 1B, the cDNA encoding the recombinant protein subcloned into the pPink-HC expression vector and expressed in yeast. The expression of COLIA1, RBD, COE and S1 were detected by western blot analysis (Supplementary Figure S2). COLIA1, RBD, COE, and S1 were demonstrated by SDS-PAGE after purification (Figure 1C).

### 3.2 S1-Trimer was predicted to be structurally similar to the PEDV S protein

**Figure 2:**
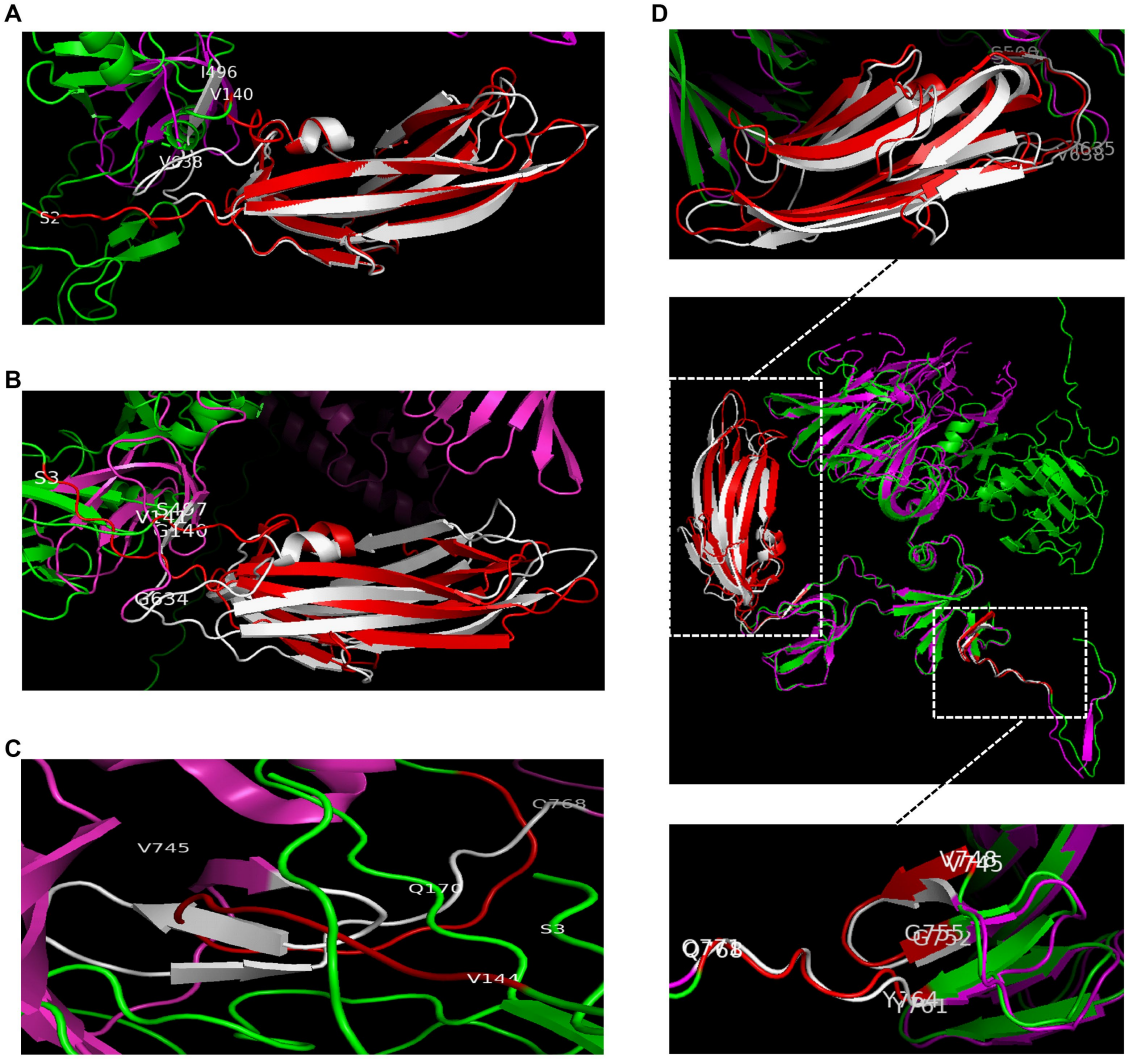
The structural differences between 6U7K and NPs. **(A)** The structural differences between 6U7K (deep pink and white) and RBD-Trimer (green and red) in residues 499–638. **(B)** The structural differences between 6U7K (deep pink and white) and COE-Trimer (green and red) in residues 499–638. **(C)** The structural differences between 6U7K (deep pink and white) and COE-Trimer (green and red) in residues 748–771. **(D)** The structural differences between 6U7K (deep pink and white) and S1-Trimer (green and red).

It is important for immunogenicity of nanovaccines that maintaining structure similarity to natural S proteins. The structures of RBD-Monomer, COE-Monomer, and S1-Monomer were predicted by the SiFold server (Supplementary Figures S3A–C). The structure of the PEDV CV777 S protein (PDB code 6U7K) was used as template to analyze the difference. No difference was observed between the structure of the RBD-Monomer and 6U7K in residues 499–638 (Figure 2A). In residues 748–771, we noticed the absence of two β-sheets in the COE-Monomer, while the structure was similar in residues 499–638 (Figures 2B,C). Furthermore, no significant difference was found between the S1-Monomer and 6U7K in residues 499–638 and 748–771 (Figure 2D). With PT-P5 (PDB code 7W6M) used as a template, the homology model of RDB-Trimer, COE-Trimer, and S1-Trimer was predicted by SWISS-MODEL; the similarity of the model and the template was 97, 85, and 97.48%, respectively. All of them could form homotrimers (Supplementary Figures S3D–F). S1-Trimer was predicted maintained the closest trimeric structure to the native PEDV S protein among the three nanoparticles.

### 3.3 Successful assembly of trimers

**Figure 3:**
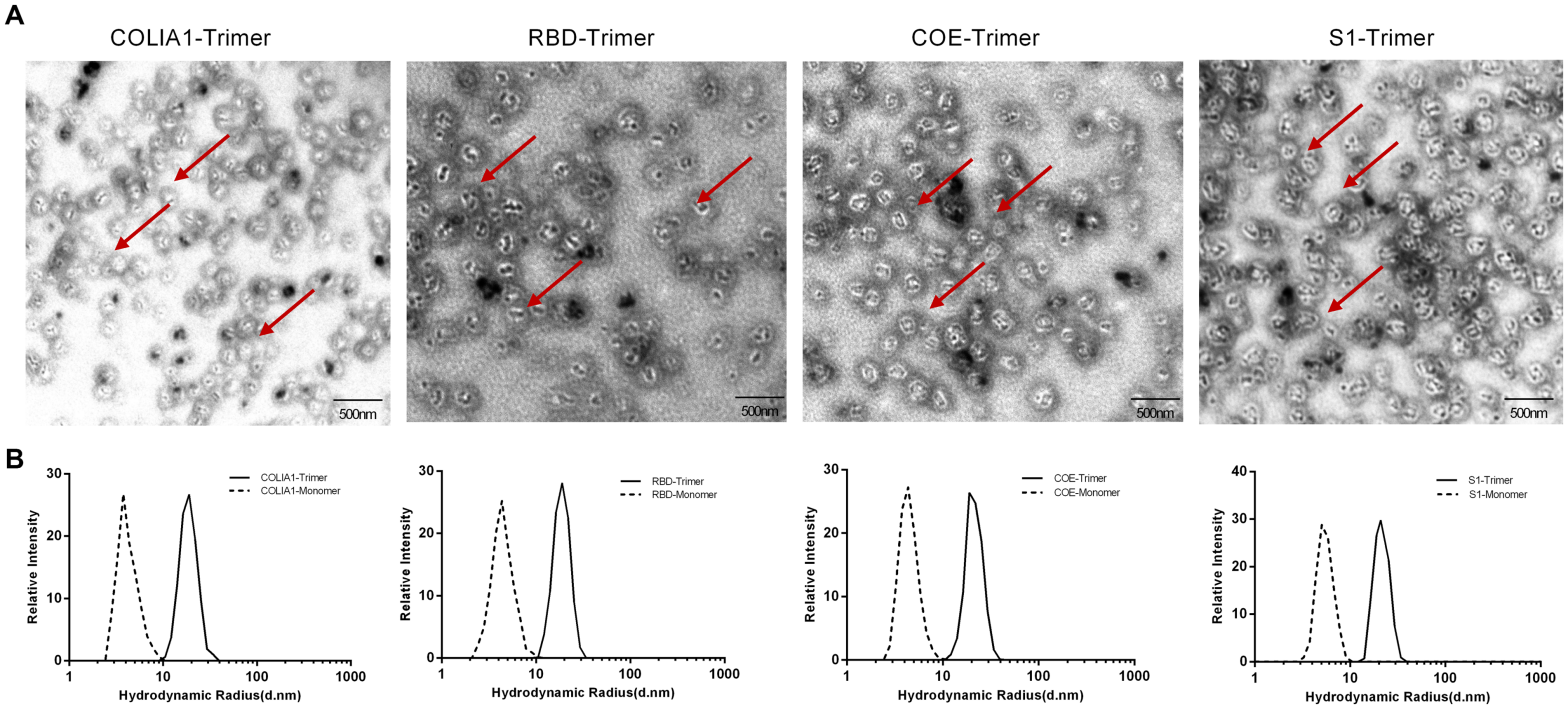
Phenotype characterization of NPs. **(A)** TEM image of COLIA1-Trimer, RBD-Trimer, COE-Trimer and S1-Trimer. The arrow indicates the trimers. **(B)** DLS determination of hydrodynamic radius (Rd) of COLIA1-Monomer/Trimer, RBD-Monomer/Trimer, COE-Monomer/Trimer and S1-Monomer/Trimer.

TEM and DSL were performed to observe the characteristics of the COLIA1-Trimer, RBD-Trimer, COE-Trimer and S1-Trimer. As shown in Figure 3A, recombinant proteins self-assembled to trimer via disulfides based on Trimer-Tag. Moreover, COLIA1-Trimer, RBD-Trimer, COE-Trimer, and S1-Trimer formed particles, as confirmed by the DLS reading at 18.92, 20.11, 20.91, and 22.66 nm, respectively. The particle size of COLIA1-Monomer, RBD-Monomer, COE-Monomer, and S1-Monomer were 3.95, 4.36, 4.44, and 5.51 nm (Figure 3B). The TEM and DSL data suggested the successful formation of COLIA1-Trimer, RBD-Trimer, COE-Trimer, and S1-Trimer.

### 3.4 NPs had stable physical properties

**Figure 4:**
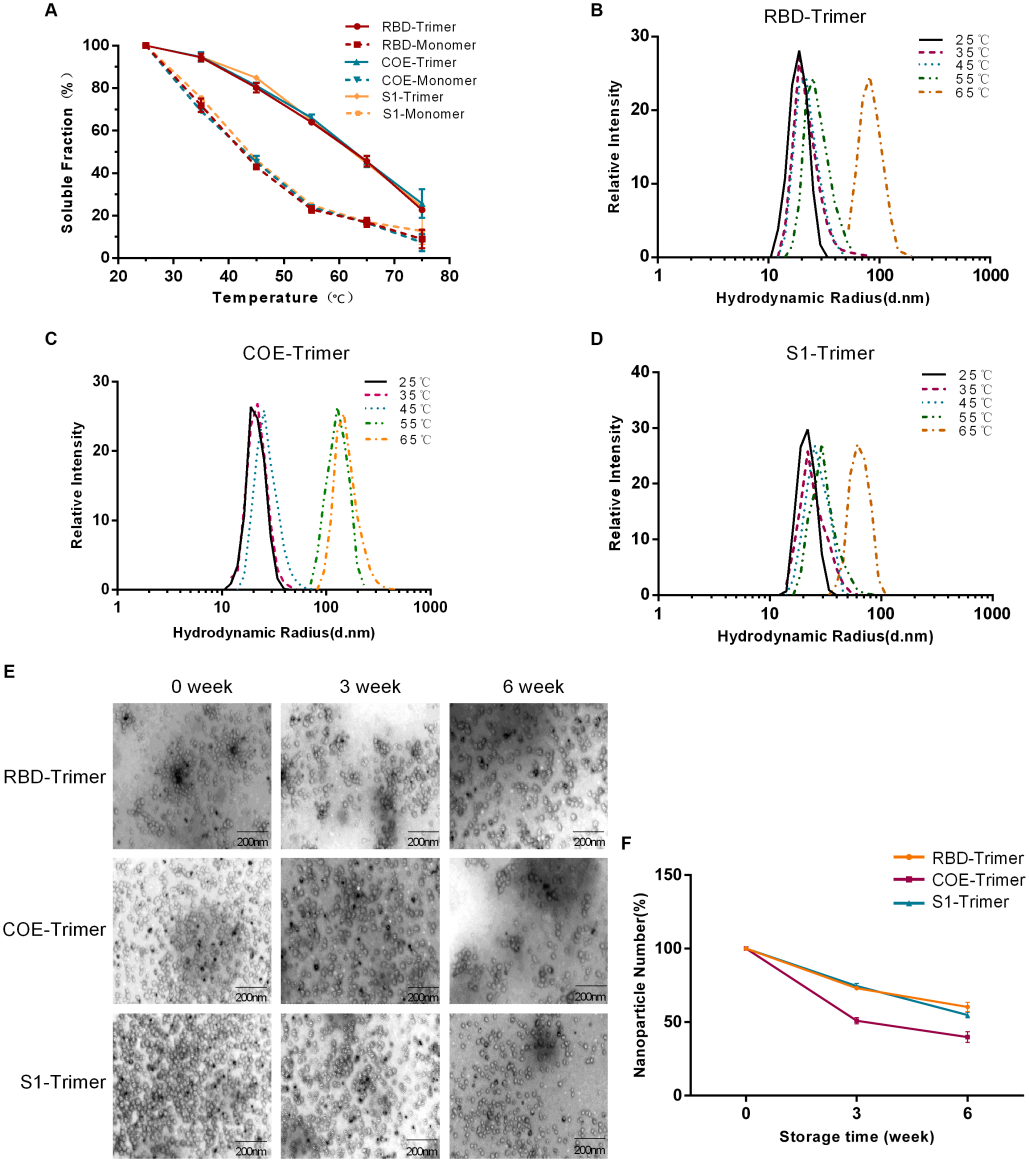
Evaluation of physical stability and long-term storage stability of NPs. **(A)** Solubility of monomeric and trimeric proteins after treatment by difference temperatures. RBD-Monomer/Trimer, COE-Monomer/Trimer and S1-Monomer/Trimer were incubated for 1 h at the indicated temperatures. The soluble protein was quantified by BCA. **(B–D)** Detection of DLS of RBD-Trimer, COE-Trimer and S1-Trimer after treatment by difference temperatures. **(E)** Long-term storage stability analysis of NPs. NPs were stored at room temperature for 0, 3 and 6 weeks, and subjected to NPs imaging by TEM. **(F)** NPs counting was calculated by Image J.

The tolerance of varying temperatures and long-term storage is an important factor in evaluating the effectiveness of vaccines. RBD-Trimer, COE-Trimer, and S1-Trimer were incubated at temperatures ranging from 25°C to 65°C for 1 h, and BCA and DSL were performed to test the heat stability. Figure 4A, reveals that in the group of trimers only ~20% of NPs were lost to aggregation at 45°C. Furthermore, ~45% of the protein remained in the soluble fraction, even when the temperature reached up to 65°C. Moreover, no significant difference was found between RBD-Trimer, COE-Trimer, and S1-Trimer. However, the soluble protein content in trimers was higher than that of monomers, i.e., only ~15% soluble protein in solution at 65°C in the group of monomers. With the temperature increases, the hydrodynamic radius of RBD-Trimer and S1-Trimer not increased until 65°C, as measured by DLS (Figures 4B,D). In addition, a significant increase in the hydrodynamic radius of COE-Trimer was observed at 55°C (Figure 4C). We also evaluated the stability of long-term storage at room temperature. After 3 and 6 weeks of maintaining the particles, NPs were analyzed by TEM to evaluate the integrity and quantity. The results indicated that the percentage of intact RBD-Trimer and S1-Trimer were over 70% for 3 weeks and up to 50% at 6 weeks. The percentage of intact COE-Trimer was ~50% at 3 weeks and ~36% at 6 weeks (Figures 4E,F). Compared with RBD-Trimer and S1-Trimer, the instability of COE-Trimer might be caused by the presence of numerous linkers presence in COE-Trimer. All data demonstrated that under general conditions, the vaccines designed in this study were robust and tolerated long-term storage and varying temperature. Moreover, RBD-Trimer and S1-Trimer exhibited improved physical stability.

### 3.5 S1-Trimer has a high level of cellular uptake, lysosome escape, and receptor affinity

**Figure 5:**
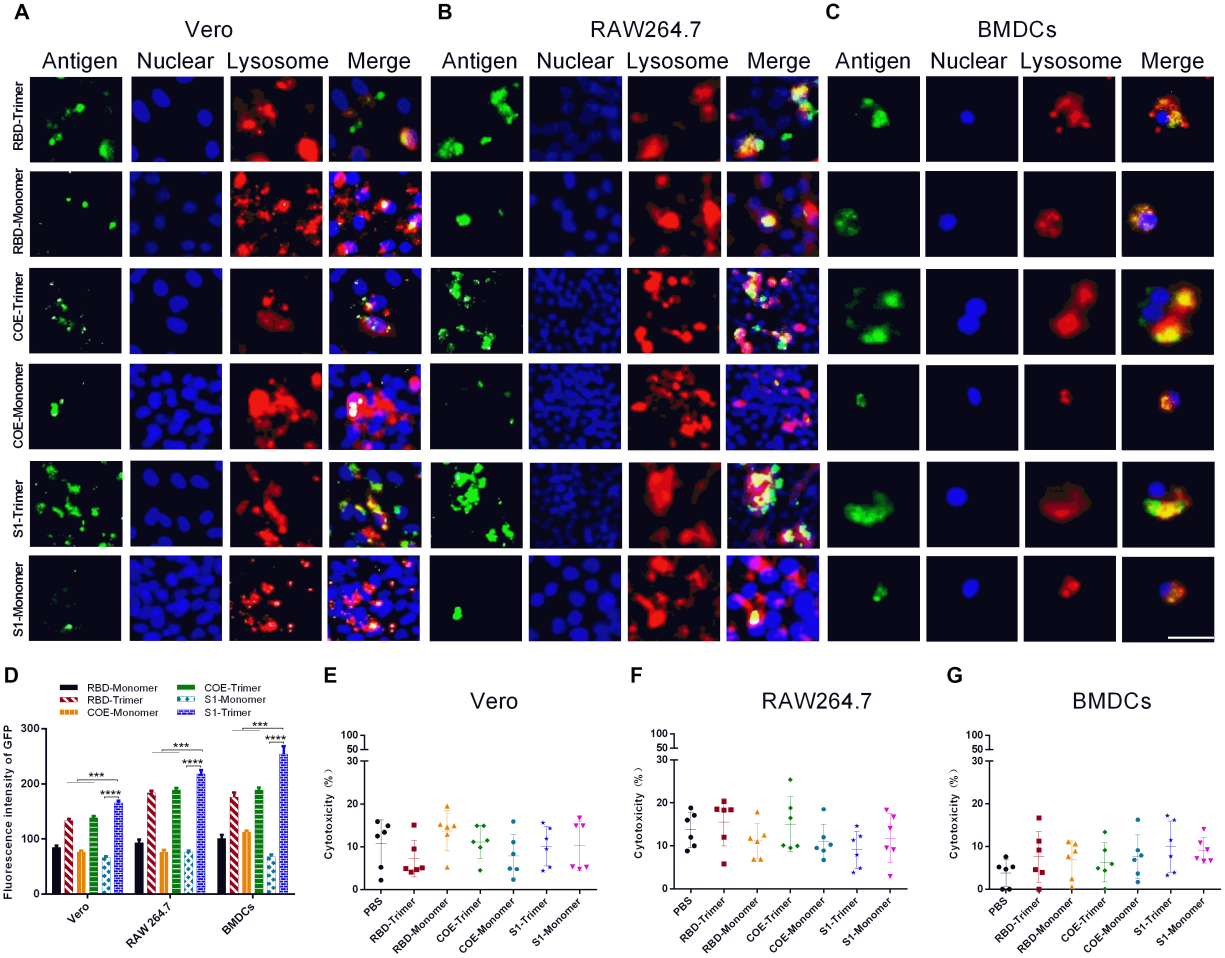
Cellular uptake and cytotoxicity of NPs in Vero, RAW264.7 and BMDCs. **(A–C)** Immunofluorescence microscopic observation of the cellular uptake, lysosomal escapeland of RBD-Monomer/Trimer, COE-Monomer/Trimer and S1-Monomer/Trimer in Vero, RAW264.7 and BMDCs. Antigens (channel 1, green), nucleus (channel 2, blue), and lysosome (channel 3, red). Scale bars, 100 μm. **(D)** Cellular uptake rates of RBD-Monomer/Trimer, COE-Monomer/Trimer and S1-Monomer/Trimer. Fluorescence intensity of GFP was determined by Image J (*n* = 3). All error bars are expressed as ±SD. ^*^p < 0.05, ^**^p < 0.01, ^***^p < 0.001, and ^****^p < 0.0001. **(E–G)** The cytotoxicity of Vero, RAW264.7 and BMDCs after treatment by RBD-Monomer/Trimer, COE-Monomer/Trimer and S1-Monomer/Trimer for 6 h. All experiments were performed independently triplicate.

The administration site had numerous antigen-presenting cells (APCs). APCs take up antigens and induce an immune response when antigens are injected into bodies intramuscularly. Furthermore, Vero cells are susceptible to PEDV (Hofmann and Wyler, 1988), and the characteristics of PEDV are usually studied using Vero cells. The S protein binds with its receptor to mediate viral entry into Vero cells. Before investigating the potential of RBD-Trimer, COE-Trimer, and S1-Trimer as nanovaccines in animal models, the cytotoxicity, cellular uptake, lysosomal escape, and the receptor affinity of RBD-Trimer, COE-Trimer, and S1-Trimer were first assessed by Vero cells and APCs (BMDCs and macrophage RAW264.7). The cellular uptake of the trimers was better, and more NPs circumvented the lysosome and diffused into the cytoplasm in comparison with the monomers. The results could be indicated by the experiments of Vero cells, BMDCs, and RAW264.7. Given that Vero cells have PEDV-specific receptors on the surface, the uptake of Vero cells can also indicate the receptor affinity between antigens and receptors. As shown in Figures 5A,D, the affinity difference between S1-Trimer and the receptor was significantly higher than that of RBD-Trimer and COE-Trimer. In addition, the cellular uptake of S1-Trimer was significantly higher than that of the other two groups (Figures 5A–D). As expected, when concentration was 10 μg/mL, no significant difference in cell viability was found between the trimers and monomers (Figures 5E–G). When the concentration of trimers and monomers reached up to 20 μg/mL, however, Vero cells and APCs were almost all dead (data not shown). All data suggested that S1-Trimer has advantages in cellular uptake, lysosomal escape, and receptor affinity in all groups.

### 3.6 Effective humoral and cellular immune responses were induced by S1-Trimer in mice

**Figure 6:**
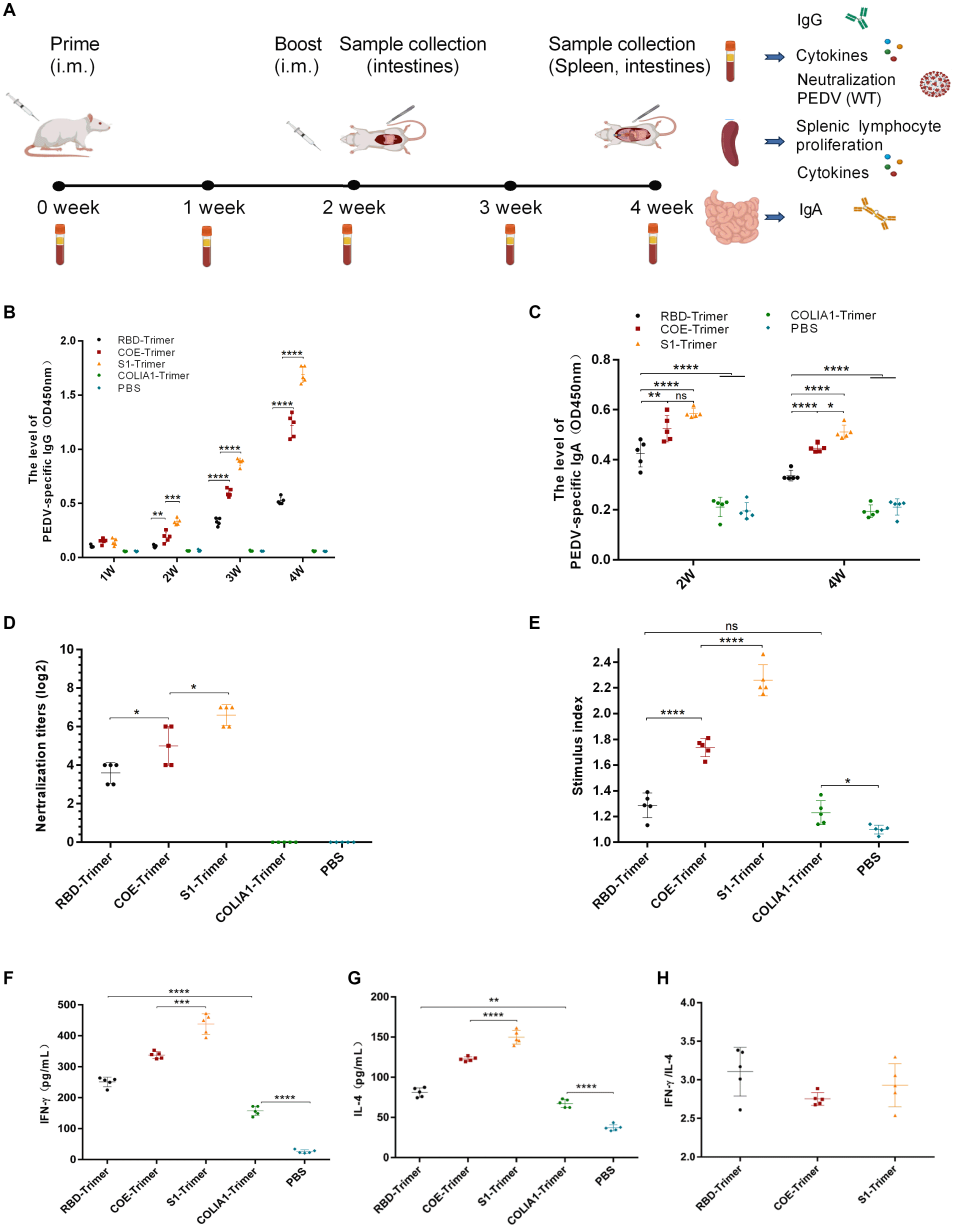
Anti-PEDV humoral and cellular immunity induced by RBD-Trimer, COE-Trimer and S1-Trimer in mice. **(A)** Schematic of experimental protocol in mice. **(B)** PEDV specific IgG in serum assayed by ELISA. **(C)** PEDV specific IgA in intestine samples assayed by an ELISA kit. **(D)** The NAbs titer in serum against infectious live PEDV. **(E)** The index of splenic T lymphocyte proliferation. **(F)** The level of IFN-γ **(G)** IL-4 in serum detected by an ELISA kit. **(H)** The ratio of IFN-γ/IL-4. All error bars are expressed as ±SD. ^*^p < 0.05, ^**^p < 0.01, ^***^p < 0.001, and ^****^p < 0.0001. ns, not significant. (All groups, *n* = 5).

Many studies indicated that the PEDV S protein included main neutralizing epitopes and the receptor-binding region could produce effective immune protection in animal models (Oh et al., 2014; Makadiya et al., 2016; Subramaniam et al., 2018). Here, we produced COLIA1-Trimer, RBD-Trimer, COE-Trimer and S1-Trimer based on Trimer-Tag technology as previously reported (Liu et al., 2017). Mice were immunized with PBS (pH = 7.4), COLIA1-Trimer (25 μg), RBD-Trimer (25 μg), COE-Trimer (25 μg), and S1-Trimer (25 μg) by intramuscular injection at zeroth week (prime) and second week (boost), respectively. Orbital blood was collected weekly, and intestines were collected on the second and fourth weeks after prime immunization. Samples were collected and stored at −20°C for further analyses. Mice were sacrificed on the fourth week (Figure 6A).

The data of mouse body weight showed that weight increased in all mice and there were no significant difference between PBS group, COLIA1-Trimer group, RBD-Trimer group, COE-Trimer group, and S1-Trimer group (Supplementary Figure S4A). It is suggested that both of nanoparticles and Trimer tag were safety for the mouse.

ELISA was used to detect PEDV-specific IgG and IgA. As shown in Figure 6B, PEDV-specific IgG was not detected in the PBS and COLIA1-Trimer groups. S1-Trimer induced the highest PEDV-specific IgG titers compared with RBD-Trimer, and COE-Trimer since the second week. A significant difference was also found between S1-Trimer, RBD-Trimer, and COE-Trimer. Furthermore, S1-Trimer induced the highest titers of PEDV-specific IgA on the second and fourth weeks after prime immunization, and the differences were significant. The titers of PEDV-specific IgA in RBD-Trimer, COE-Trimer, and S1-Trimer, however, decreased on the fourth week compared with the second week (Figure 6C).

Sera were collected on the fourth week and were used to detect NAbs titers to understand the ability of IgG against PEDV. The NAbs of S1-Trimer against PEDV were significantly higher than RBD-Trimer and COE-Trimer vaccinated groups (Figure 6D).

The proliferation of spleen T-lymphocyte was studied further. The data from Figure 6E suggest that COLIA1-Trimer, RBD-Trimer, COE-Trimer, and S1-Trimer could promote T-lymphocyte proliferation. A significant increase in stimulation index was were induced by the trimer vaccinated group compared with PBS group. Furthermore, S1-Trimer was more effective in stimulating the proliferation of spleen T-lymphocytes compared with RBD-Trimer and COE-Trimer.

Antigen-specific cell-mediated immunity (CMI) was studied by analyzing the level of IFN-γ and IL-4 from immunized mice sera and splenocytes by ELISA and RT-qPCR. The data revealed that S1-Trimer induced the highest level of IFN-γ and IL-4 in sera and mediated high expressions of IFN-γ and IL-4 at the mRNA level in splenocytes (Figures 6F,G; Supplementary Figures S5A,B). In addition, RBD-Trimer, COE-Trimer and S1-Trimer exhibited Th1-biased CMI response, in which the TH1/TH2 cytokine ratio [IFN γ/interleukin-4–positive (IL-4)] was more than 1 (Figure 6H; Supplementary Figure S5C).

All data indicated that S1-Trimer exhibited optimal levels of humoral and CMI response in mice.

### 3.7 Effective humoral and cellular immune responses were induced by S1-Trimer in sows

**Figure 7:**
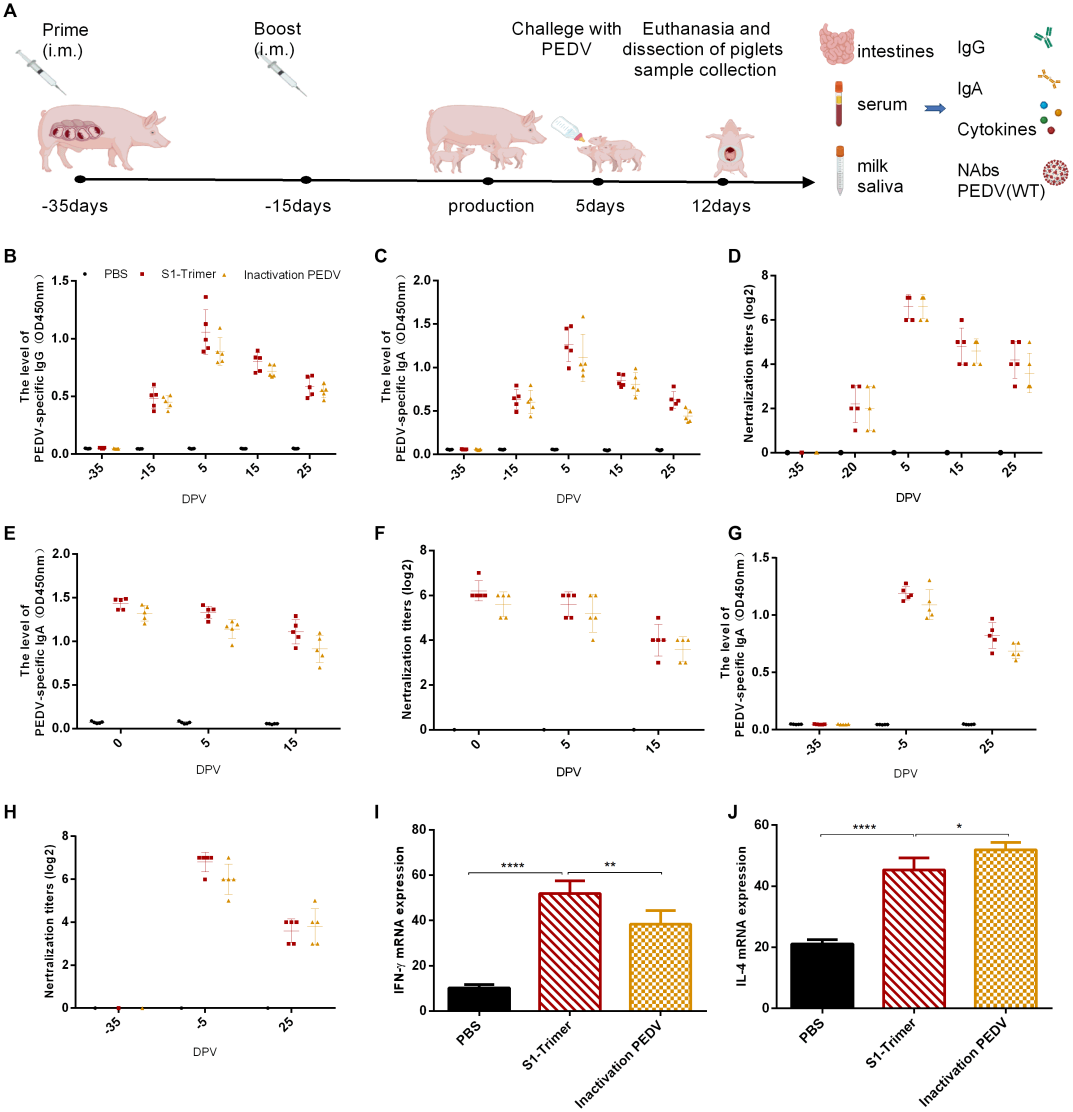
Anti-PEDV humoral and cellular immunity induced by S1-Trimer in sows. **(A)** Schematic of experimental protocol in sow. **(B)** PEDV specific IgG in serum assayed by ELISA. **(C)** PEDV specific IgA in serum assayed by ELISA. **(D)** The NAbs titer in serum against infectious live PEDV. **(E)** PEDV specific IgA in milk assayed by ELISA. **(F)** The NAbs titer in milk against infectious live PEDV. **(G)** PEDV specific IgA in saliva assayed by ELISA. **(H)** The NAbs titer in saliva against infectious live PEDV. **(I)** The level of IFN-γ and **(J)** IL-4 mRNA expression in PBMCs assayed by qRT-PCR and normalized to that of GAPDH. All error bars are expressed as ±SD. ^*^p < 0.05, ^**^p < 0.01, and ^****^p < 0.0001. (All groups, *n* = 5).

To evaluate the ability of S1-Trimer induced prophylactic NAbs against PEDV, we immunized sows with S1-Trimer or inactivated PEDV vaccine (Figure 7A). The S1-Trimer and inactivated vaccine were safe that no sow death or clinical symptoms including anorexia, diarrhea, abortion, and suppuration, necrosis at the inoculation site were observed. The S1-Trimer induced a high titer of PEDV S-specific IgG and IgA antibodies. The titers of IgG and IgA were similar to those in the inactivated PEDV vaccine group (Figures 7B,C,E,G). As shown in Figures 7D,F,H, the S1-Trimer and inactivated PEDV vaccines induced high titers of NAbs in serum, milk, and saliva samples. Moreover, S1-Trimer induced high expression of IFN-γ and IL-4 at the mRNA level in PBMCs. The level of IFN-γ and IL-4 induced by the S1-Trimer was significantly higher than that by the PBS group (Figures 7I,J). In summary, S1-Trimer induces potent humoral and cellular immune responses in sows.

### 3.8 S1-Trimer protected piglets against PEDV

**Figure 8:**
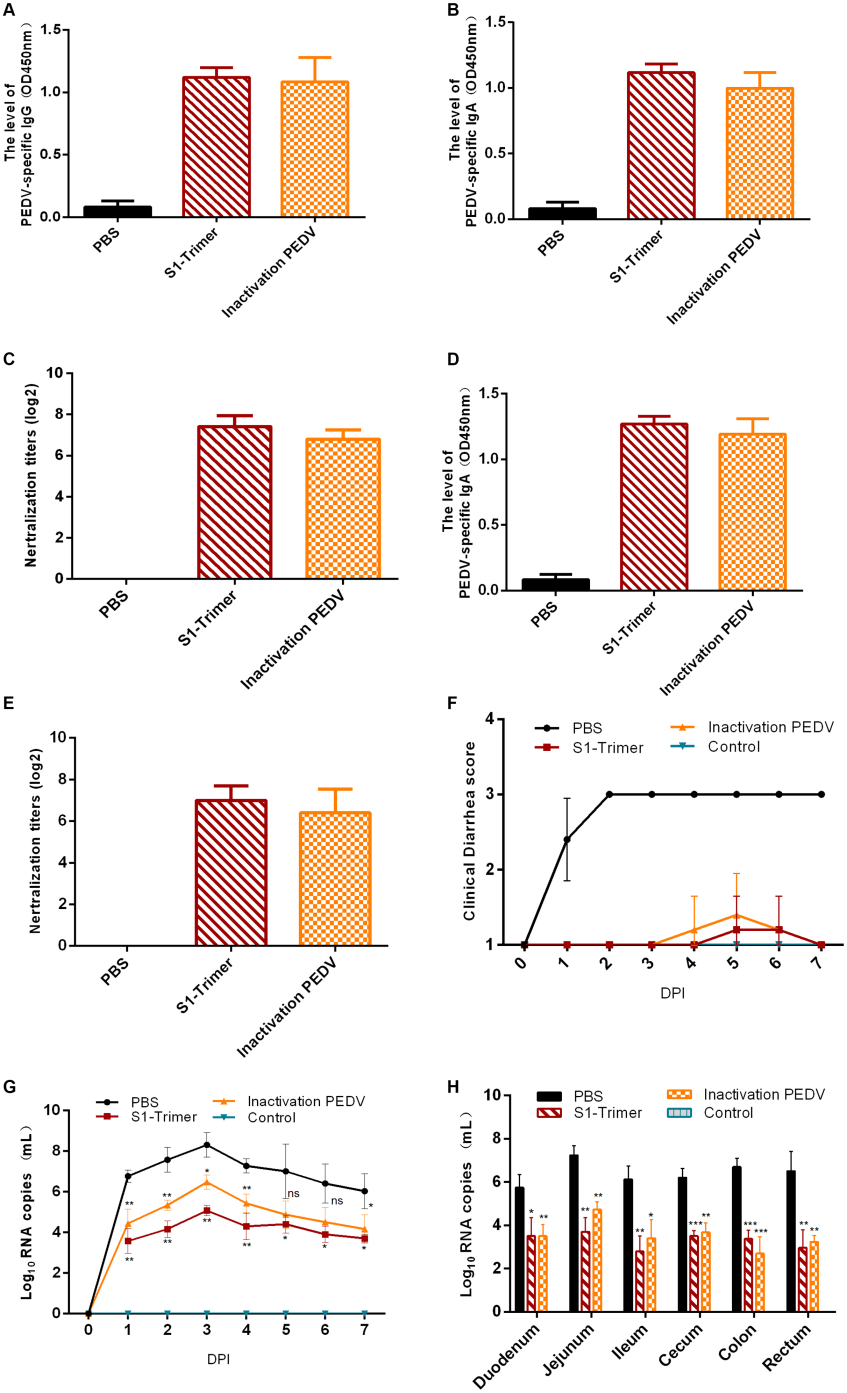
Protection of 5-day-old piglets from vaccinated sows. **(A)** PEDV specific IgG in serum assayed by ELISA. **(B)** The NAbs titer in serum against infectious live PEDV. **(C)** PEDV specific IgA in intestines assayed by ELISA. **(D)** The NAbs titer in intestines against infectious live PEDV. **(E)** Titers of NAbs against infectious live PEDV were measured in intestine samples. **(F)** The scores of piglets’ diarrhea. **(G)** The viral load in fecal swabs. **(H)** The viral load in different intestinal segments. All error bars are expressed as ±SD. ^*^p < 0.05, ^**^p < 0.01, ^***^p < 0.001, and ^****^p < 0.0001. ns, not significant. (All groups, *n* = 5).

Five-day-old piglets from the S1-Trimer immunization group, the inactivated PEDV vaccine immunization group, and the PBS group challenged with PEDV GS2022 (3 mL × 10^4.5^ TCID_50_/mL per piglets) were used to estimate the protection of S1-Trimer. The data of piglets body weight showed that after challenged with GS2022, weight increased in control group, S1-Trimer group, and inactivated vaccine group. And the temperature of piglets in control group, S1-Trimer group, and inactivated vaccine group maintained 38–39°C. However, the body weight and temperature of piglets in the PBS group decreased (Supplementary Figures S4B,C). Sera and intestines were collected, and the level of IgG, IgA, and NAbs was detected. Similar to the inactivated vaccine group, high levels of IgG and IgA were detected in the serum of the S1-Trimer group (Figures 8A,B). The S1-Trimer and inactivated vaccine induced high levels of NAbs in serum (Figure 8C). In addition, S1-Trimer and inactivated vaccine induced high levels of IgA and NAbs in the small intestines (Figures 8D,E).

**Figure 9:**
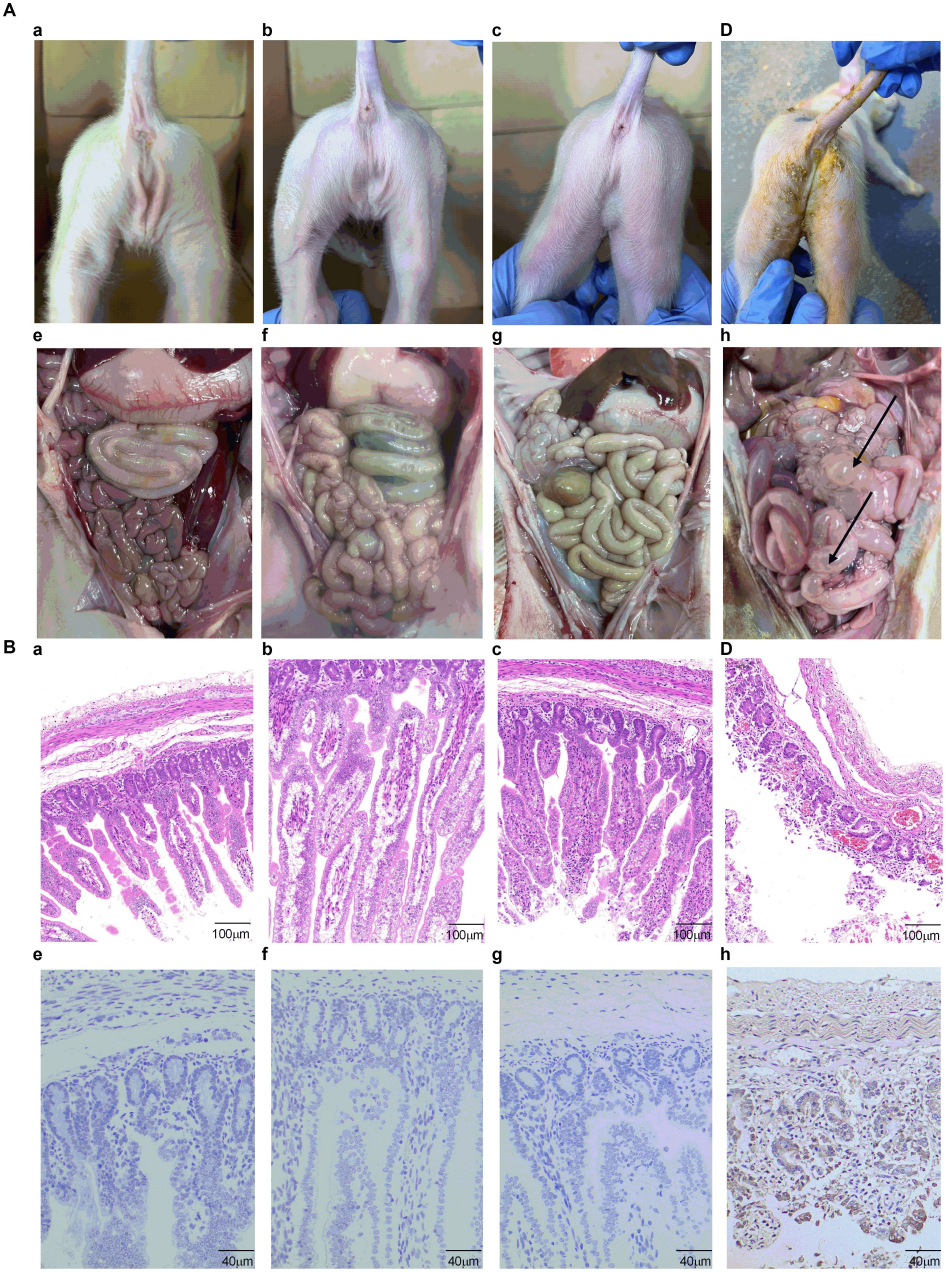
The analysis of clinical symptoms, necropsy examination, histopathological and IHC analysis. **(A)** Clinical symptoms of piglets of **(a)** control group, **(b)** S1-Trimer group, **(c)** inactivated PEDV vaccine group, and **(d)** PBS group. The necropsy examination of piglets of **(e)** control group, **(f)** S1-Trimer group, **(g)** inactivated PEDV vaccine group, and **(h)** PBS group. **(B)** Histopathological analysis in jejunum of **(a)** control group, **(b)** S1-Trimer group, **(c)** inactivated PEDV vaccine group. **(d)** PBS group. IHC analysis in jejunum of **(e)** control group, **(f)** S1-Trimer group, **(g)** inactivated PEDV vaccine group, **(h)** PBS group.

The clinical symptoms were monitored and scored in accordance with the severity of their diarrhea. At 1 DPI, mild diarrhea with softening feces and watery diarrhea were observed in the PBS group. At 2 DPI, all piglets developed severe diarrhea with yellow watery feces (Figure 8F; Supplementary Figure S6A) and vomited with yellow vomitus containing undigested flocculent milk (Supplementary Figure S6B). Only one piglet in the S1-Trimer group had mild diarrhea at 5 DPI. Two piglets in the inactivated vaccine group had mild diarrhea, at 4–5 DPI. Diarrhea was not observed on the seventh day. The feces of the control group piglets were all normal (Figure 8F; Supplementary Figure S6C). After being infected with PEDV, piglets in the S1-Trimer group, the inactivated vaccine group, and the control group did not show any clinical symptoms (Figures 9Aa–c).

Viral load in fecal swabs and intestinal segments was detected by RT-qPCR. In the control group, PEDV RNA was detected in all fecal swabs collected between 1 and 7 DPI and peaked at 3 DPI. The viral load in the fecal swabs of the S1-Trimer group was significantly lower than that of PBS group. No PEDV RNA was detected in the fecal swab samples of the control group (Figure 8G). The distribution of PEDV in different intestinal segments was performed after necropsy. As shown in Figure 8H, after challenged with PEDV, all intestinal segments including the duodenum, jejunum, ileum, cecum, colon, and rectum had PEDV distribution, and viral load was highest in the jejunum. Moreover, the levels were significantly lower in vaccinated piglets compared with PBS piglets.

Histopathology and IHC were used to estimate the extent of intestinal issues. The small intestines of the PBS group were filled with gas and expanded, and the intestinal wall became thin and transparent (Figure 9Ah). By contrast, no macroscopic intestinal tissues were observed in the control group, S1-Trimer, and inactivated vaccine groups (Figures 9Ae–g). Histopathologic analysis showed jejunum intestinal villus and gland necrosis, and the villus ruptured and shed, while interstitial blood vessels were congested in PBS group (Figure 9Bd). By contrast, no pathological injuries were observed in the intestines of the control, S1-Trimer, and inactivated vaccine groups (Figures 9Ba–c). For the intestine lesion score (Supplementary Figure S7A), the ratios of villus length to crypt depth (V:C) of jejunum tissues from the control, S1-Trimer and inactivated vaccine were more than 3:1, while villi or crypt was absent in the PBS group. The result of IHC suggested that in the PBS group, the PEDV antigen was distributed mainly in the cytoplasm of atrophic villous epithelial cells (Figure 9Bh). No viral antigens were observed in the control, S1-Trimer, and inactivated vaccine groups (Figures 9Be–g). The percentage of positive area was significantly lower in the vaccination group compared with the PBS group (Supplementary Figure S7B).

All data suggested that S1-Trimer could provide protection against PEDV challenge in piglets.

## 4 Discussion

The outbreak of PEDV has caused huge losses in the global pig industry. Nowadays, commercialized PEDV vaccines mainly about attenuated and inactivated vaccines unable to protect pig from PEDV. Thus, an effective vaccine is still urgently needed. The S protein is the target of PEDV vaccine designs, because it located on the virus’ surface and it induces the production of NAbs. NP vaccines have the advantage of inducing immune response, and the rapid development of self-assembled NP vaccines provides new ideas for PEDV vaccine. NPs are easy to produce, cost low and are safe (Bruun et al., 2018). For example, a novel swine fever E2 self-assembled NP vaccine based on the Mi3 platform triggered an effective immune response in mice (Liu et al., 2021), and data for TRAIL-Trimer showed stronger antitumor efficacy (Liu et al., 2017). Moreover, NPs of SARS-CoV-2 protected animals against the challenge of SARS-CoV-2 (Kang et al., 2021; Liang et al., 2021).

In the present study, we used the Trimer-Tag platform to produce PEDV RBD-Trimer, COE-Trimer, and S1-Trimer. The data, revealed that S1-Trimer exhibited advantages as a candidate vaccine, in comparison with RBD-Trimer and COE-Trimer. S1-Trimer was predicted to maintain structure similar to that of natural PEDV S protein. Maintaining a trimeric structure similar to natural S proteins is necessary. A previous study showed that the lack of a complete trimeric structure made it difficult for COVID-19 RBD to capture the entire antibody (Faustini et al., 2021). The natural trimeric S-glycoprotein made epitope presentation more complete (Petherick, 2020). The fusion of a viral antigen and Trimer-Tag formed a stable disulfide bond-linked homotrimer. Therefore, NPs were produced in this study to possess relatively high thermal stability in temperatures ranging from 25°C to 65°C, except for COE-Trimer. Even if RBD-Trimer and S1-Trimer are stored at room temperature for 6 weeks, more than 50% of NPs are still preserved, and they exhibit high particle size uniformity and long-term stability. For the structure of COE-Trimer, multiple neutralizing epitopes linked by a linker may be the cause of instability.

The size of NPs was similar to pathogens of about 10–200 nm. Therefore, APCs uptook NPs preferentially and activated adaptive immunity (Graham et al., 2019). APCs had the advantage of absorption and internalization of NP-delivered antigens (Rehm, 2017). Thus, trimers were easier to be uptaken by cells than monomers. The absorption rate of particles varies greatly with the size of NPs, with larger particles slowing down. The optimal NP size was 20–50 nm (dos Santos et al., 2011). In our study, the diameters of RBD-Trimer, COE-Trimer, and S1-Trimer were ~20 nm with no significant difference. Given the presence of PEDV-specific receptors on the surface of Vero cells, receptor-mediated endocytosis was a pathway for Vero cells to uptake RBD-Trimer, COE-Trimer, and S1-Trimer. The rote of cellular uptake of S1-Trimer was significantly higher in Vero cells, indicating that S1-Trimer had the highest affinity for receptors. Previous research found that enveloped viruses have membrane FPs, which could efficiently escape lysosomes by changing their shape and fusing with targeted membranes when pH decreases. For example, hemagglutinin was the peptide of influenza viruses, which could help the virus disrupt the stability of lysosomal membranes and enter the cytoplasm (Ferrer-Miralles et al., 2008). The FPs made it easier for S1-Trimer to enter cells. These observations may explain why S1-Trimer had advantages of cellular uptake, lysosomal escape, and the receptor affinity compared with RBD-Trimer and COE-Trimer.

The presently study reported that subunit vaccine immunization was effective in humoral immune response but could not induce CMI response (Kardani et al., 2016). Given the Trimer-Tag platform, S1-Trimer exhibited high immunogenicity in humoral immune response and CMI response. In the mouse model, S1-Trimer not only induced high levels of PEDV-specific IgG, PEDV-specific IgA, and NAbs; it also promoted the proliferation of spleen T-lymphocytes and mediated Th1-biased CMI response. Fusion to Trimer-Tag allows the soluble wild-type S1 protein to form a disulfide bond-linked homotrimer. Moreover S1 remains noncovalently bound to Trimer-Tag, thus preserving the crucial antigenic epitopes necessary for viral neutralization. Compared with RBD-Trimer and COE-Trimer, S1-Trimer contained more complete antigen epitopes. Therefore, S1-Trimer induced higher NAbs against PEDV infection in the mouse model. Moreover, the highly organized structure of VLP was the key component for boosting IgA responses (Onodera et al., 2019). As a VLP, S1-Trimer has highly organized structure. Therefore, S1-Trimer drives mucosal IgA, which has also been confirmed in the piglet model.

For neonatal suckling piglets, the most promising and effective immunization route against enteric diseases was lactogenic immunity (Chattha et al., 2015; Langel et al., 2016). Previous studies showed that the most effective method to induce a strong sIgA response was the oral route (Zhang et al., 2020). In our study, S1-Trimer induced high levels of PEDV-specific IgG and IgA in serum, milk, and saliva. The NAbs level was similar to that of the inactivated PEDV vaccine group. S1-Trimer also mediated CMI response and induced high expression of IFN-γ and IL-4 in sow PBMCs. After immunization, pathogen-specific IgA+ plasmablasts were transported to the mammary gland and sIgA accumulated in milk. The data suggested that the protection had transferred to the suckling pig through passive immunity. High levels of IgG, IgA, and NAbs were observed in the serum and intestinal samples of piglets. After being challenged with PEDV, only one piglet in the S1-Trimer group displayed mild diarrhea, and lower viral RNA copies in the feces and intestinal tissues were detected in comparison with to the piglets in the PBS group.

In summary, we provided S1-Trimer, a candidate NP vaccine for PEDV based on Trimer-Tag. A successful PEDV vaccine must have four essential characteristics: safety, efficacy, scalability of manufacturing and distribution, and speed of development (Corey et al., 2020). The results of our study suggested PEDV S1-Trimer based on the Trimer-Tag platform is easy and quick to produce. S1-Trimer had great physical stability, high temperature resistance, and tolerance to long-term storage. Moreover, S1-Trimer was safe and robust and can induce high levels of humoral and cell-mediated immune responses in animal models. S1-Trimer also could protect piglets against the challenge of PEDV. Overall, the results of our study can help the development of effective PEDV vaccines to prevent PEDV outbreak.

## Data availability statement

The original contributions presented in the study are included in the article/Supplementary material, further inquiries can be directed to the corresponding author.

## Ethics statement

The animal studies were approved by the Animal Ethics Committee of Lanzhou Veterinary Research Institute, Chinese Academy of Agriculture Sciences (Approval number: LVRIAEC-2023-050; LVRIAEC-2023-053), and all animal procedures were performed in accordance with the Animal Ethics Procedures and Guidelines of the People’s Republic of China. The studies were conducted in accordance with the local legislation and institutional requirements. Written informed consent was obtained from the owners for the participation of their animals in this study.

## Author contributions

LL: Writing – original draft, Writing – review & editing. SY: Conceptualization, Writing – original draft, Writing – review & editing. JZ: Investigation, Visualization, Writing – review & editing. LZ: Methodology, Writing – original draft. ZT: Funding acquisition, Writing – review & editing. LQ: Formal analysis, Writing – review & editing. YW: Methodology, Writing – review & editing. JY: Data curation, Writing – original draft. HZ: Methodology, Writing – original draft. YD: Data curation, Writing – review & editing. XL: Methodology, Writing – original draft. SS: Formal analysis, Writing – original draft. HG: Formal analysis, Funding acquisition, Supervision, Writing – original draft, Writing – review & editing.

## Footnotes

^1^ www.boson-tech.cn

^2^ https://swissmodel.expasy.org/
