# Supplementary figures and images for: Spike 1 trimer, a nanoparticle vaccine against porcine epidemic diarrhea virus induces protective immunity challenge in piglets
Source: Front Microbiol. 2024 Apr 8;15:1386136. doi: 10.3389/fmicb.2024.1386136 (PMC11033347; doi:10.3389/fmicb.2024.1386136)

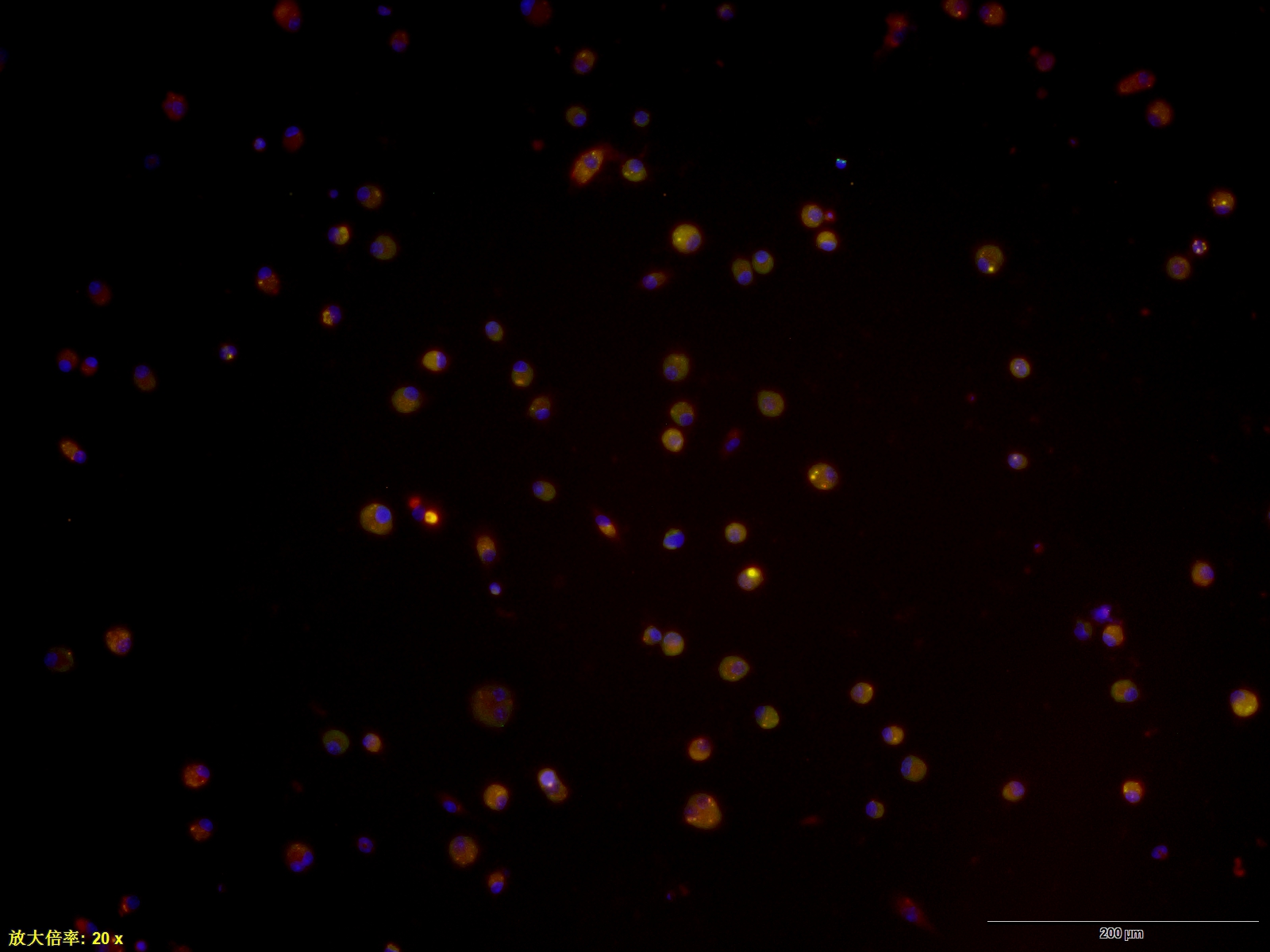

Supplement: Supplementary file 3 [file Data_Sheet_1.ZIP › cellular uptake-BMDC/COE-Monomer/图像_04.jpg]

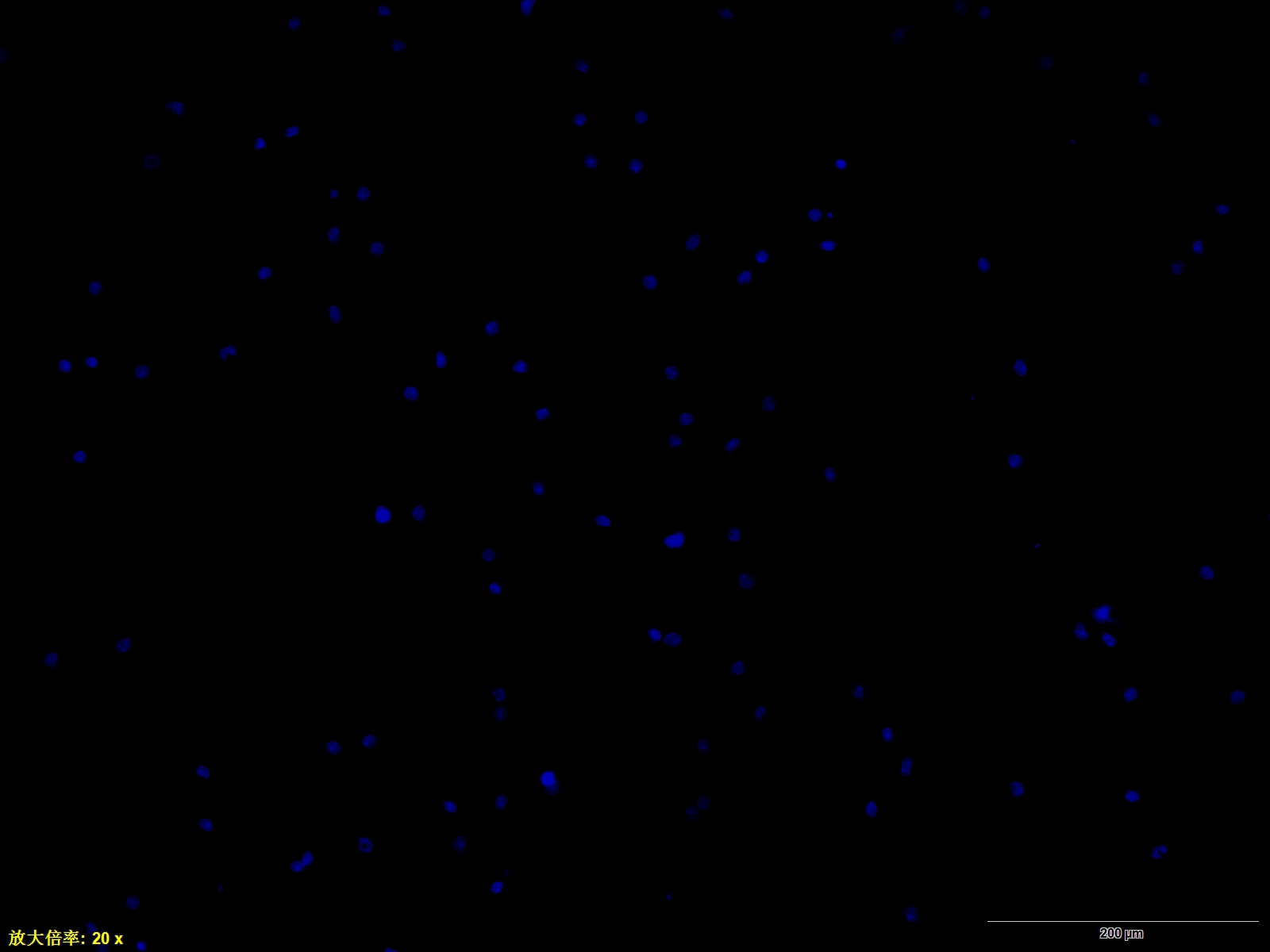

Supplement: Supplementary file 3 [file Data_Sheet_1.ZIP › cellular uptake-BMDC/COE-Monomer/图像_21869.jpg]

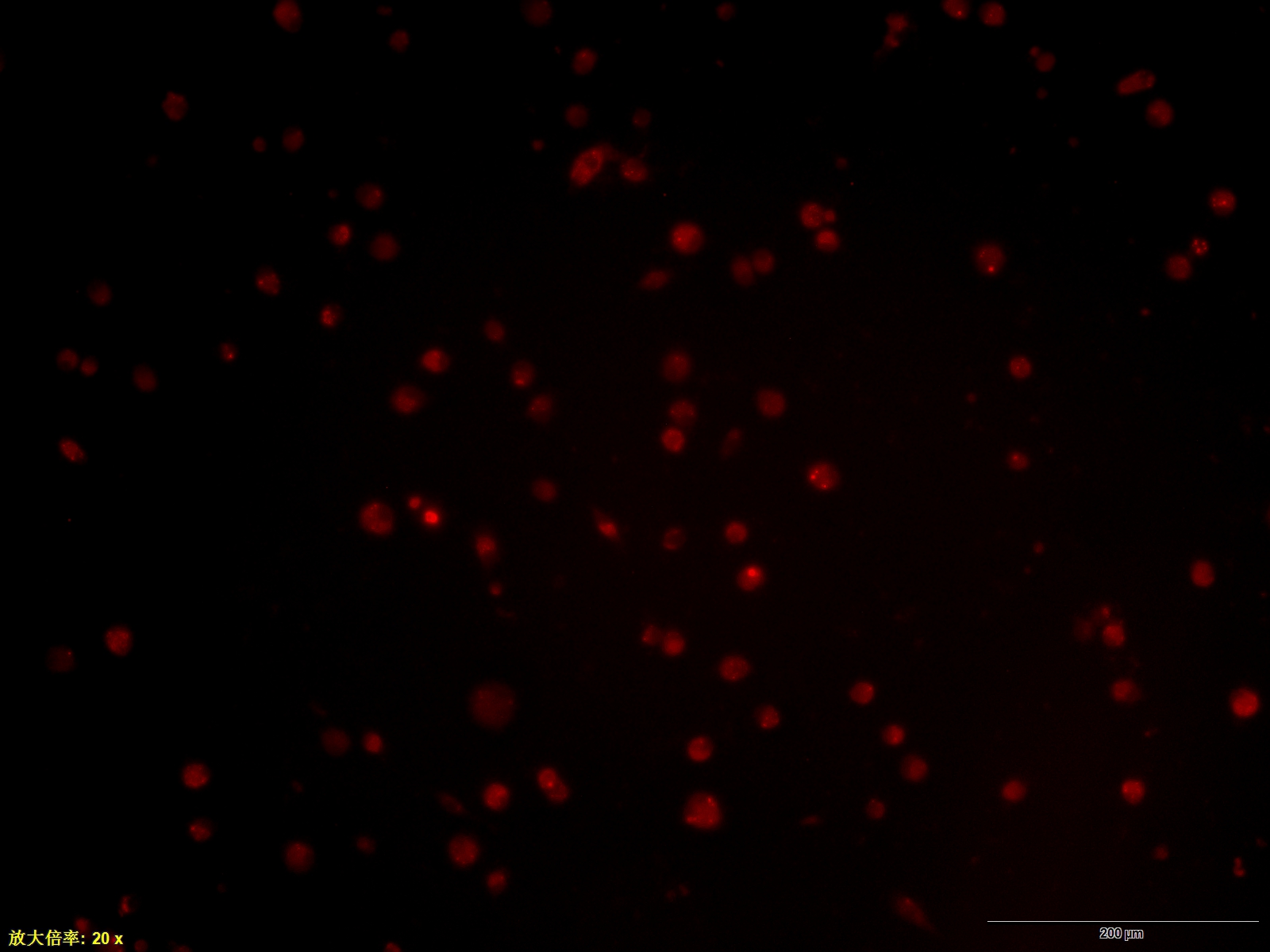

Supplement: Supplementary file 3 [file Data_Sheet_1.ZIP › cellular uptake-BMDC/COE-Monomer/图像_21871.jpg]

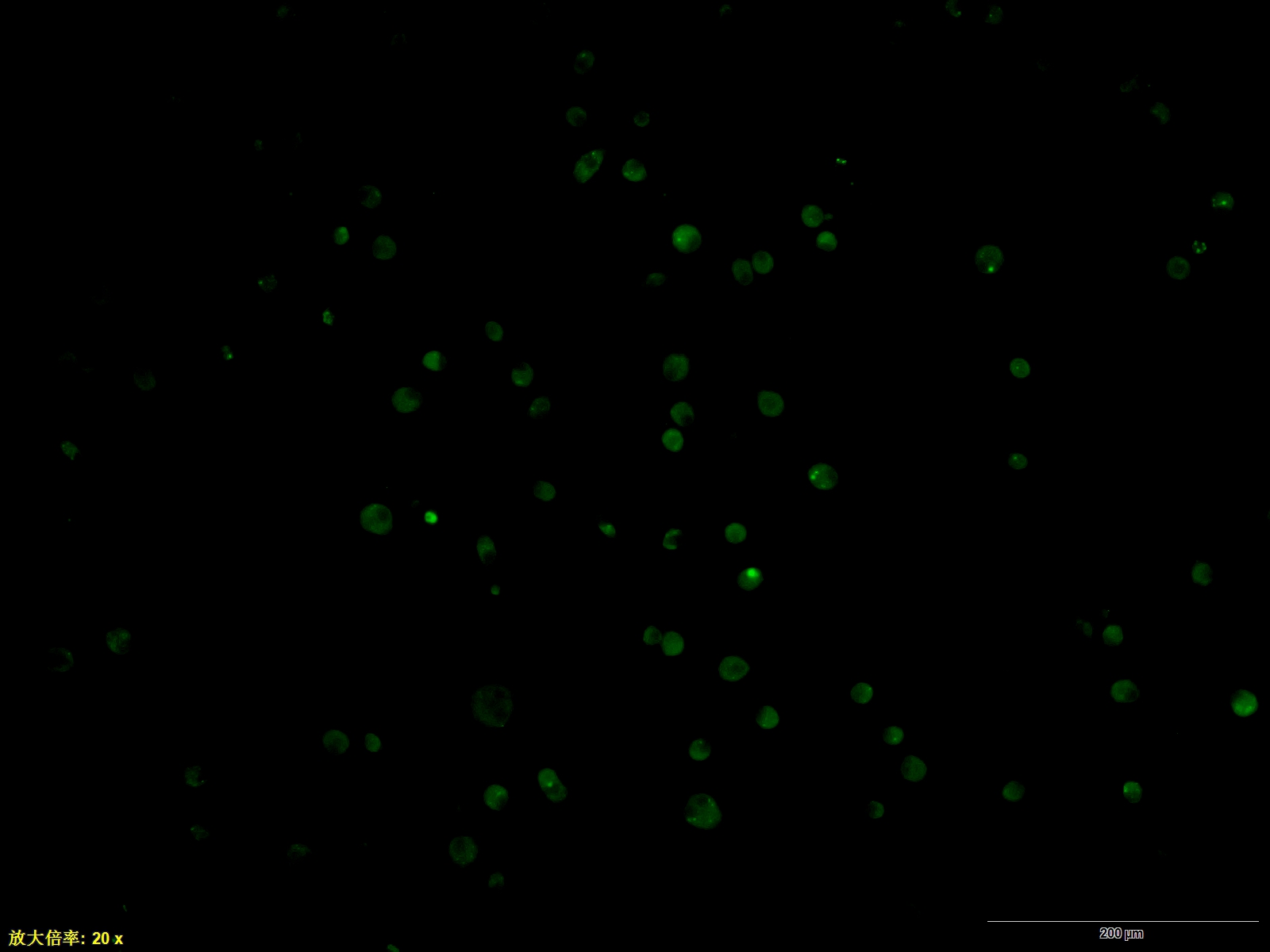

Supplement: Supplementary file 3 [file Data_Sheet_1.ZIP › cellular uptake-BMDC/COE-Monomer/图像_21873.jpg]

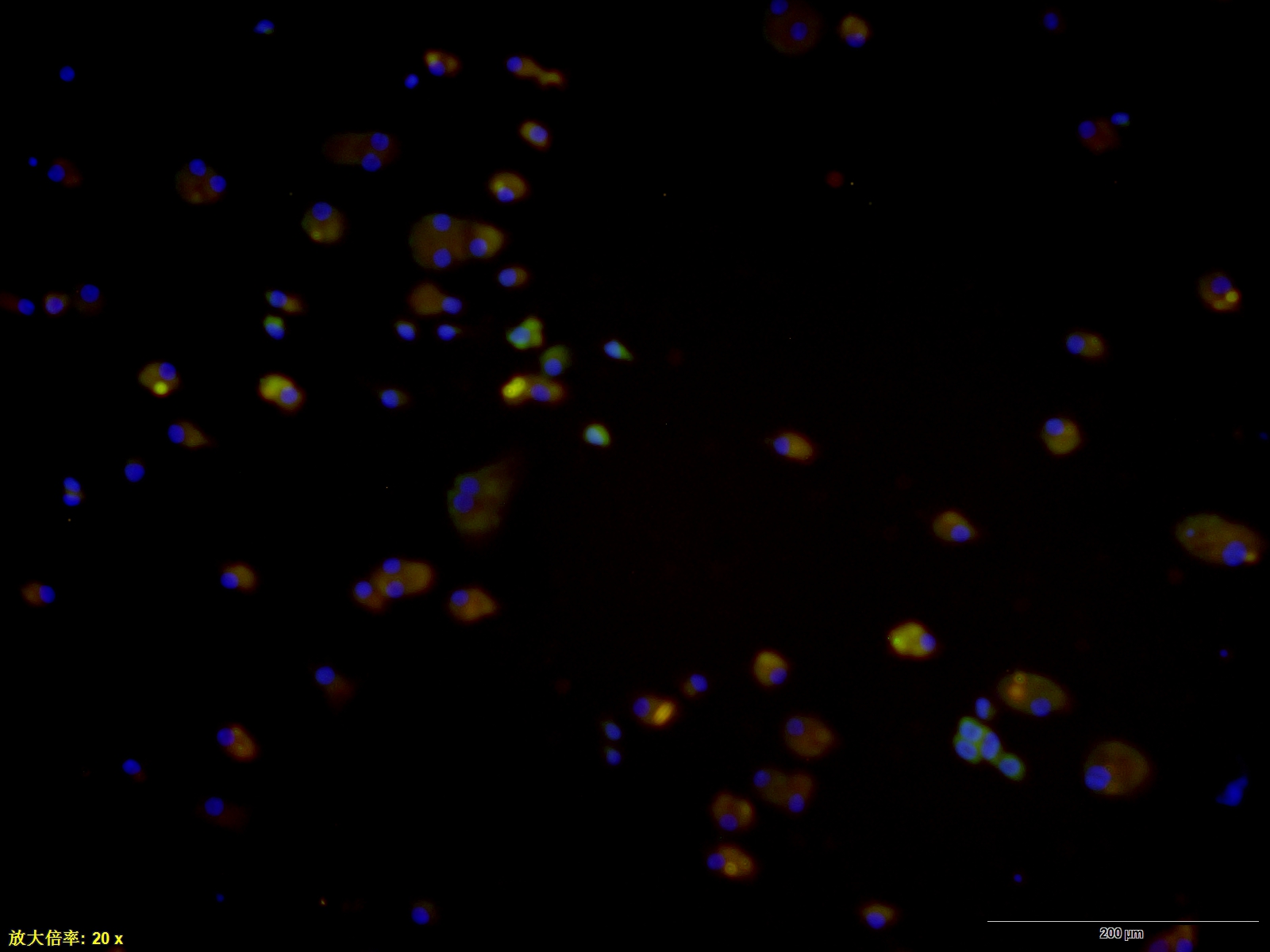

Supplement: Supplementary file 3 [file Data_Sheet_1.ZIP › cellular uptake-BMDC/COE-Trimer/图像_01.jpg]

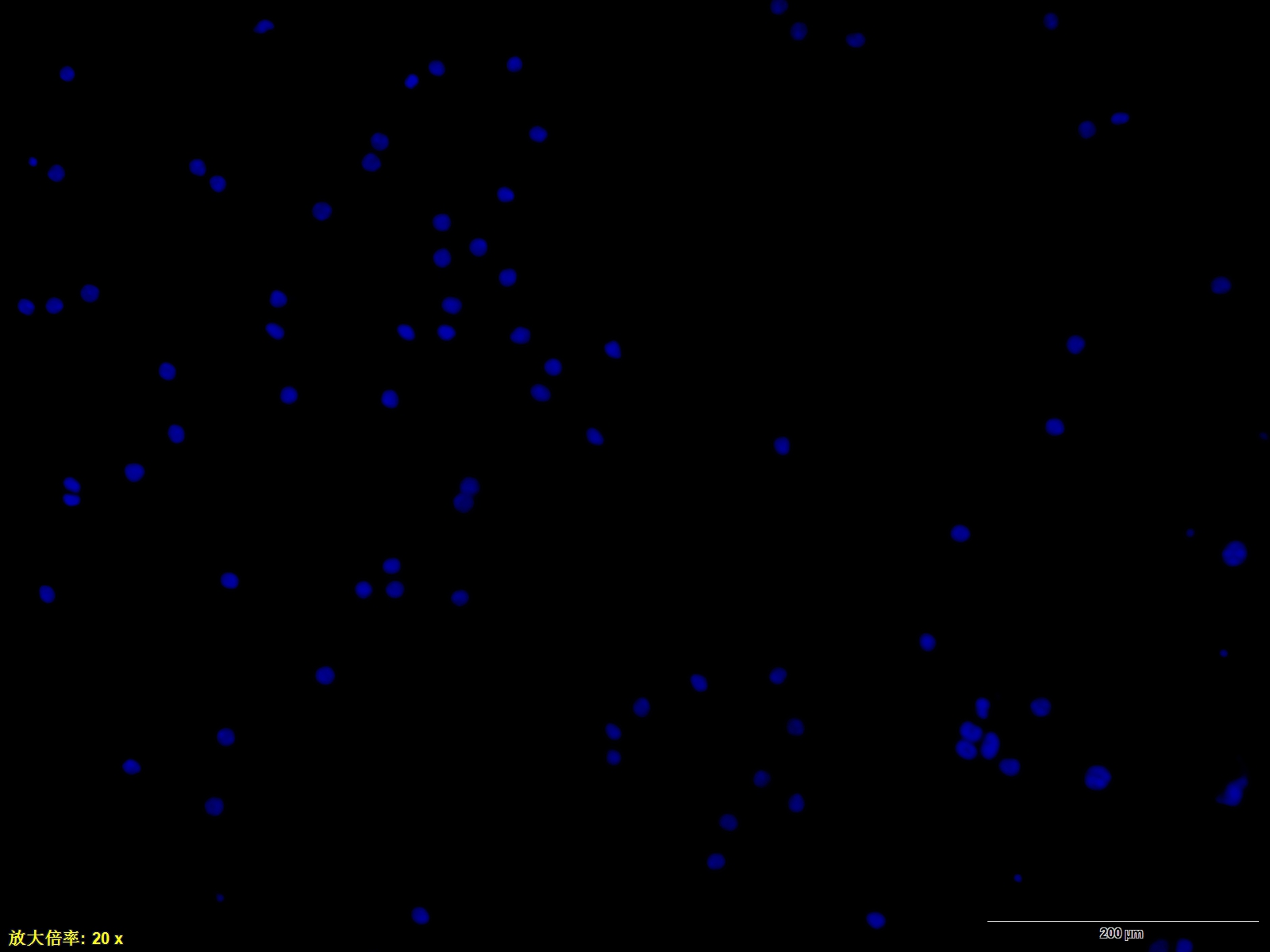

Supplement: Supplementary file 3 [file Data_Sheet_1.ZIP › cellular uptake-BMDC/COE-Trimer/图像_21859.jpg]

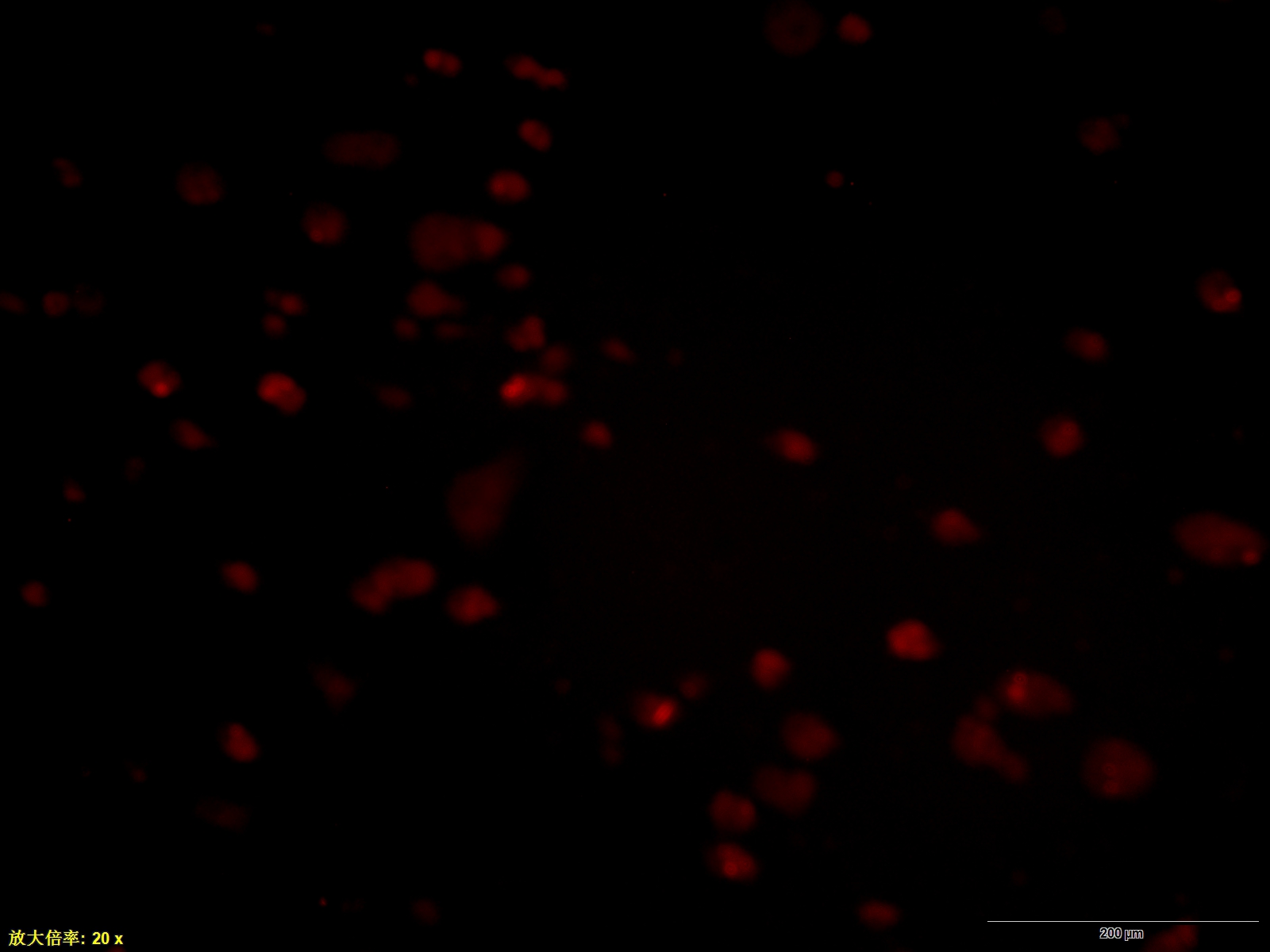

Supplement: Supplementary file 3 [file Data_Sheet_1.ZIP › cellular uptake-BMDC/COE-Trimer/图像_21860.jpg]

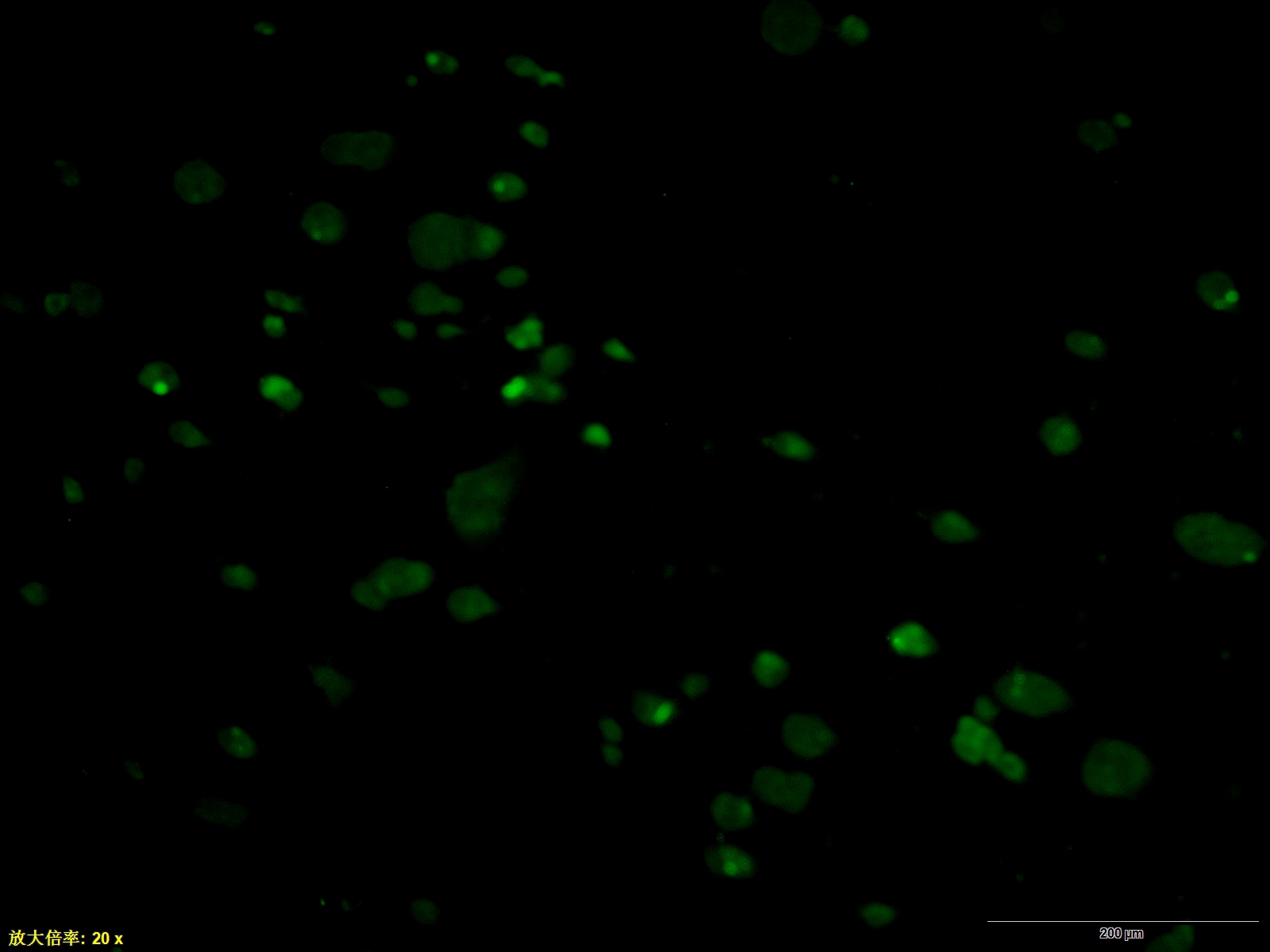

Supplement: Supplementary file 3 [file Data_Sheet_1.ZIP › cellular uptake-BMDC/COE-Trimer/图像_21862.jpg]

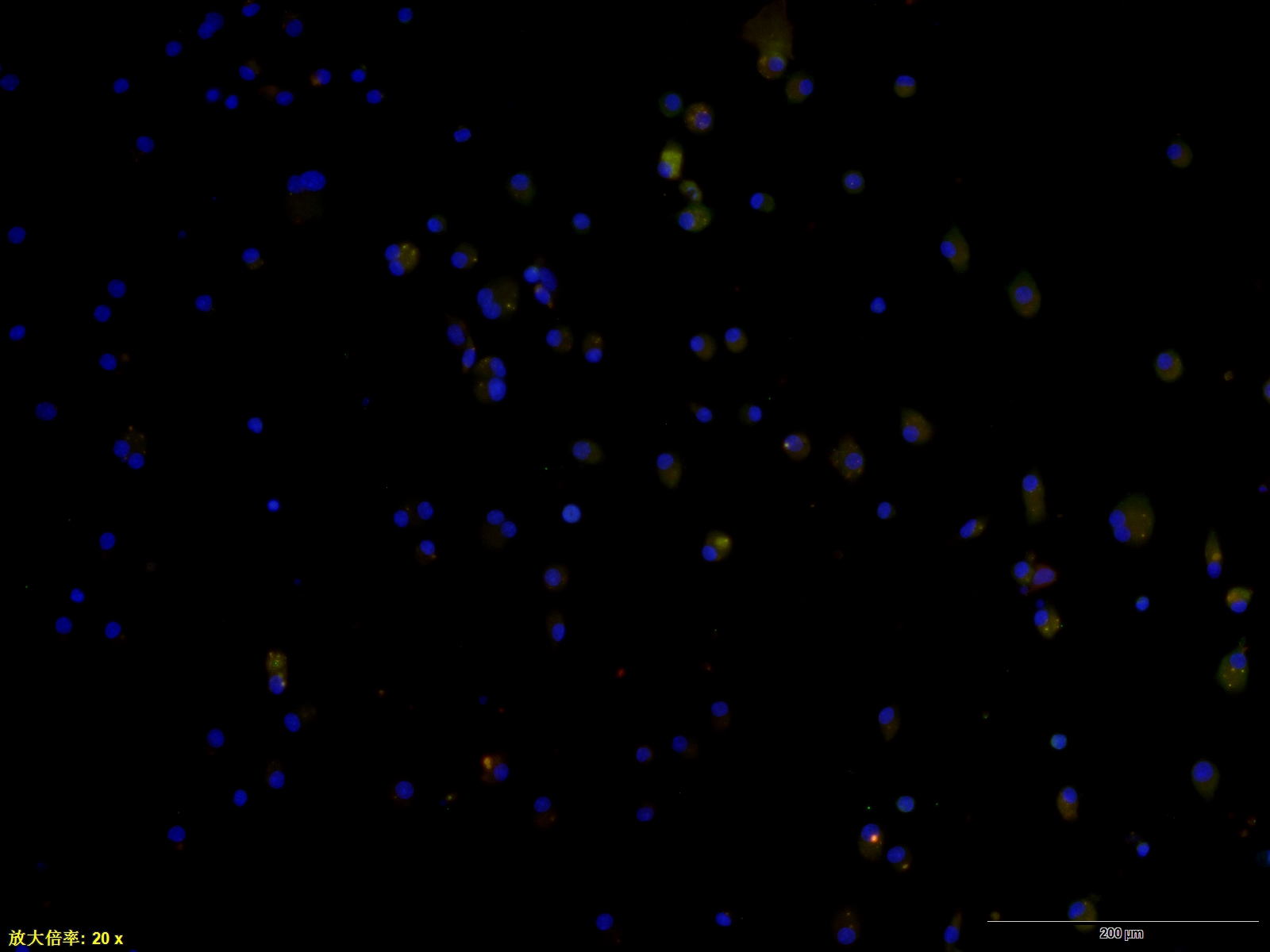

Supplement: Supplementary file 3 [file Data_Sheet_1.ZIP › cellular uptake-BMDC/RBD-Monomer/图像_01.jpg]

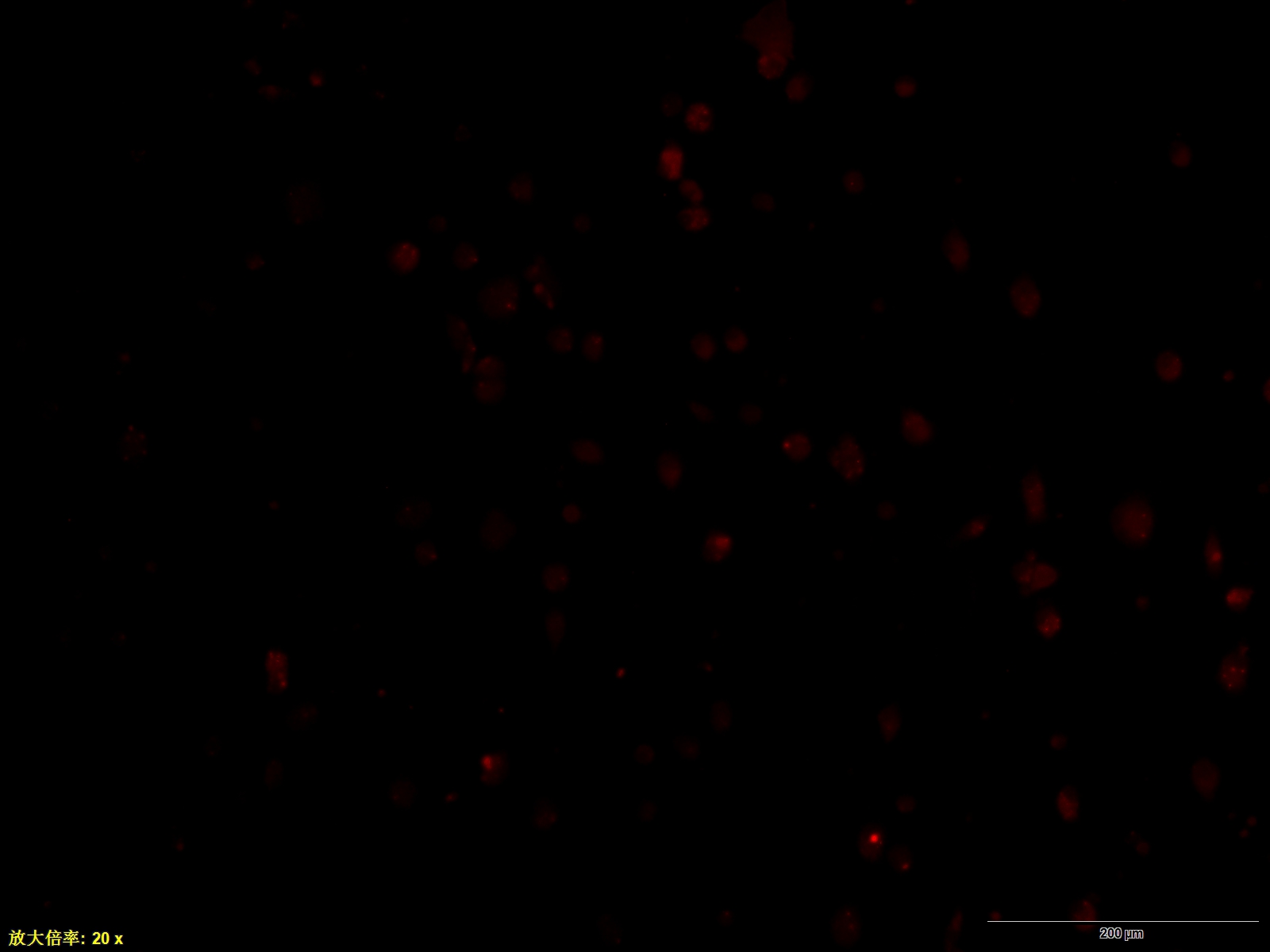

Supplement: Supplementary file 3 [file Data_Sheet_1.ZIP › cellular uptake-BMDC/RBD-Monomer/图像_21875.jpg]

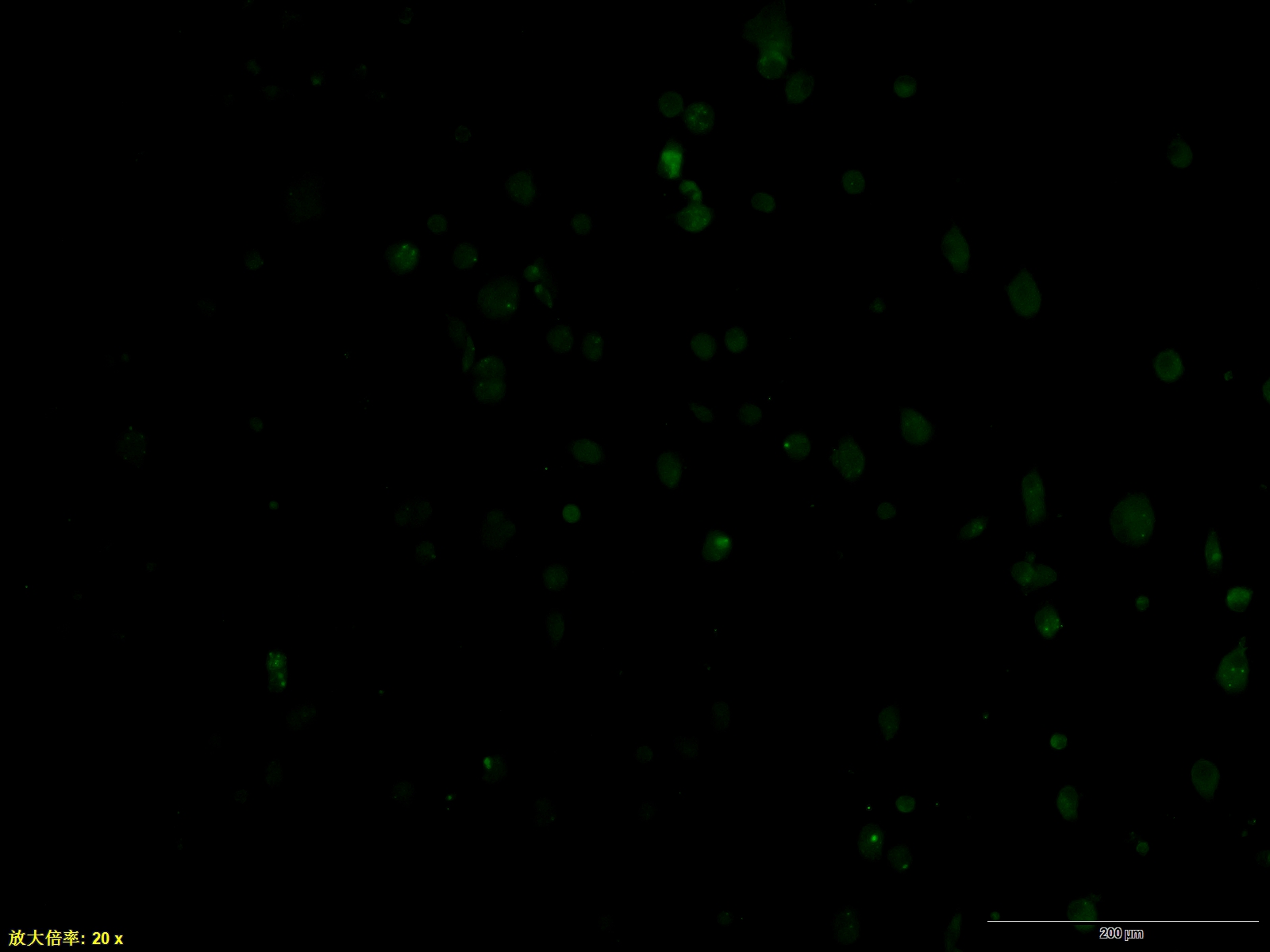

Supplement: Supplementary file 3 [file Data_Sheet_1.ZIP › cellular uptake-BMDC/RBD-Monomer/图像_21876.jpg]

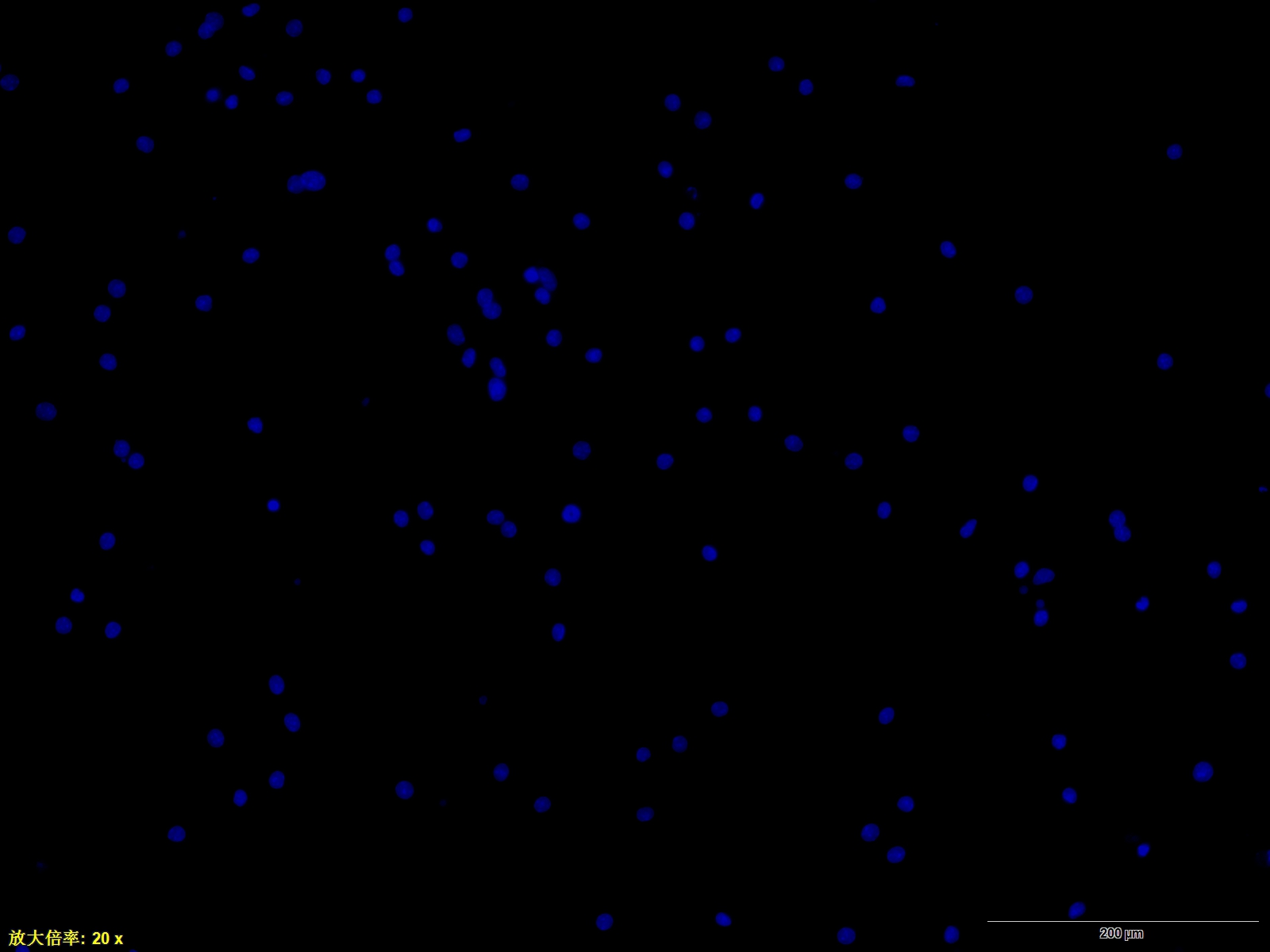

Supplement: Supplementary file 3 [file Data_Sheet_1.ZIP › cellular uptake-BMDC/RBD-Monomer/图像_21877.jpg]

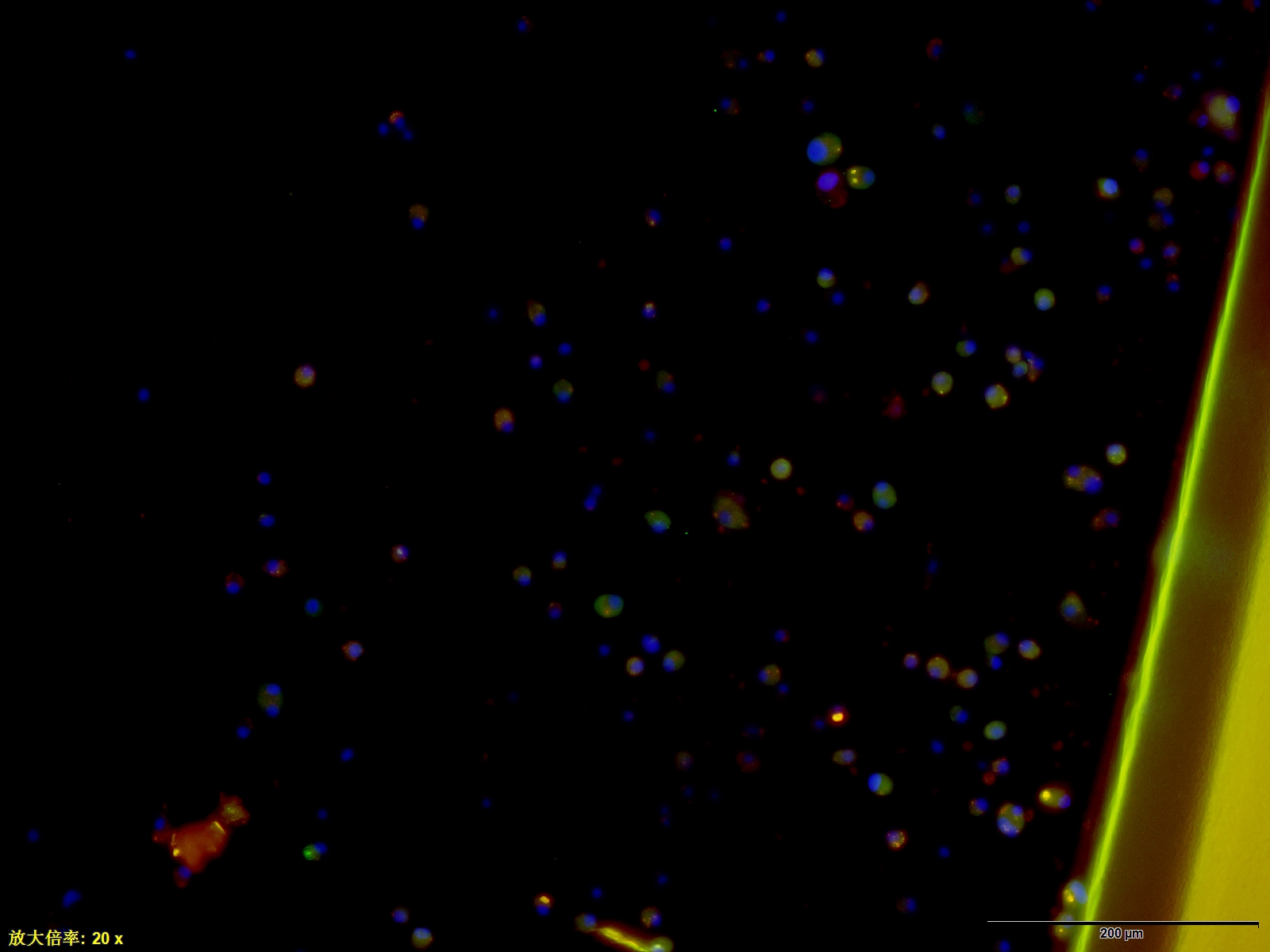

Supplement: Supplementary file 3 [file Data_Sheet_1.ZIP › cellular uptake-BMDC/RBD-Trimer/图像_01.jpg]

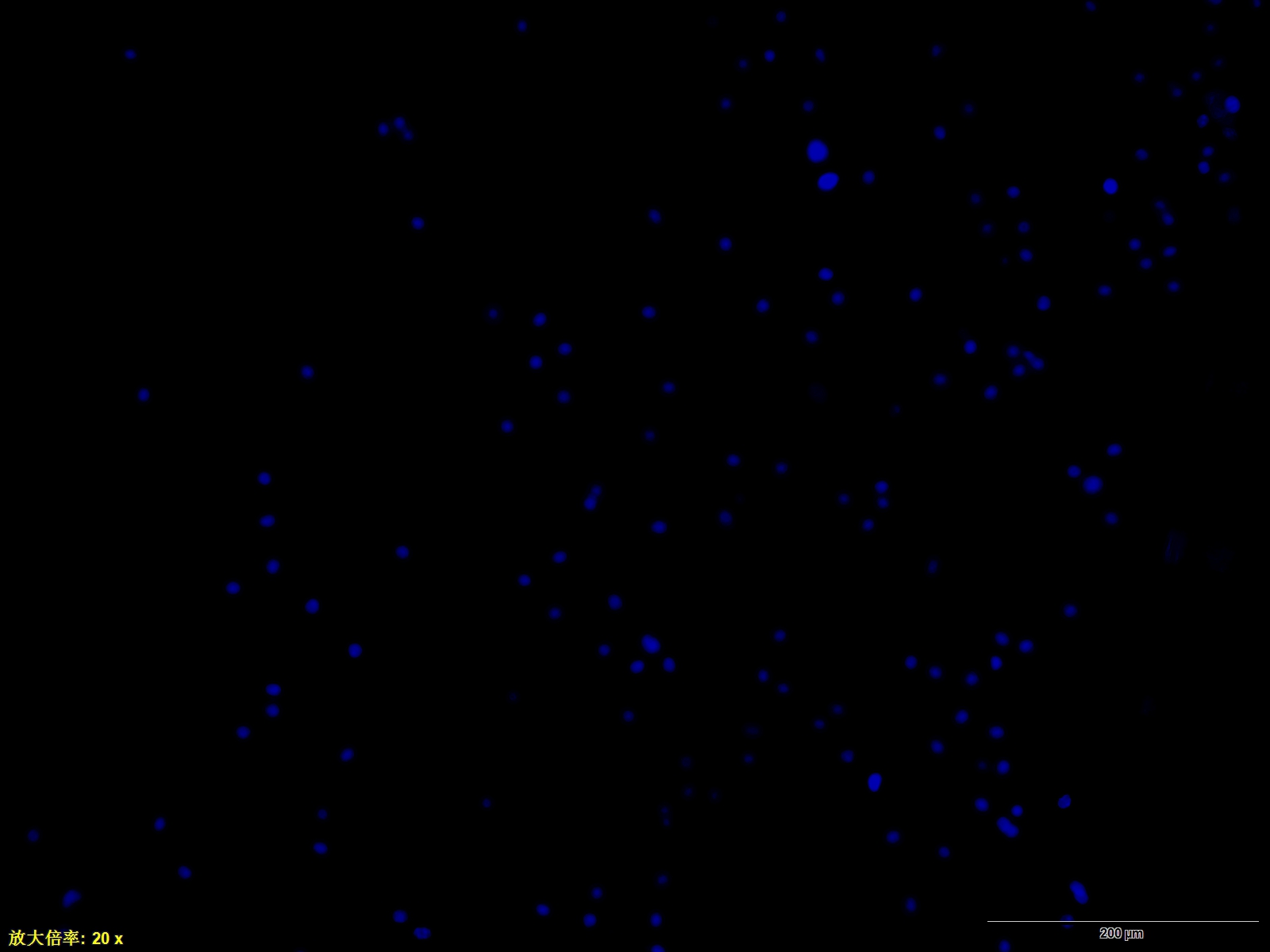

Supplement: Supplementary file 3 [file Data_Sheet_1.ZIP › cellular uptake-BMDC/RBD-Trimer/图像_21866.jpg]

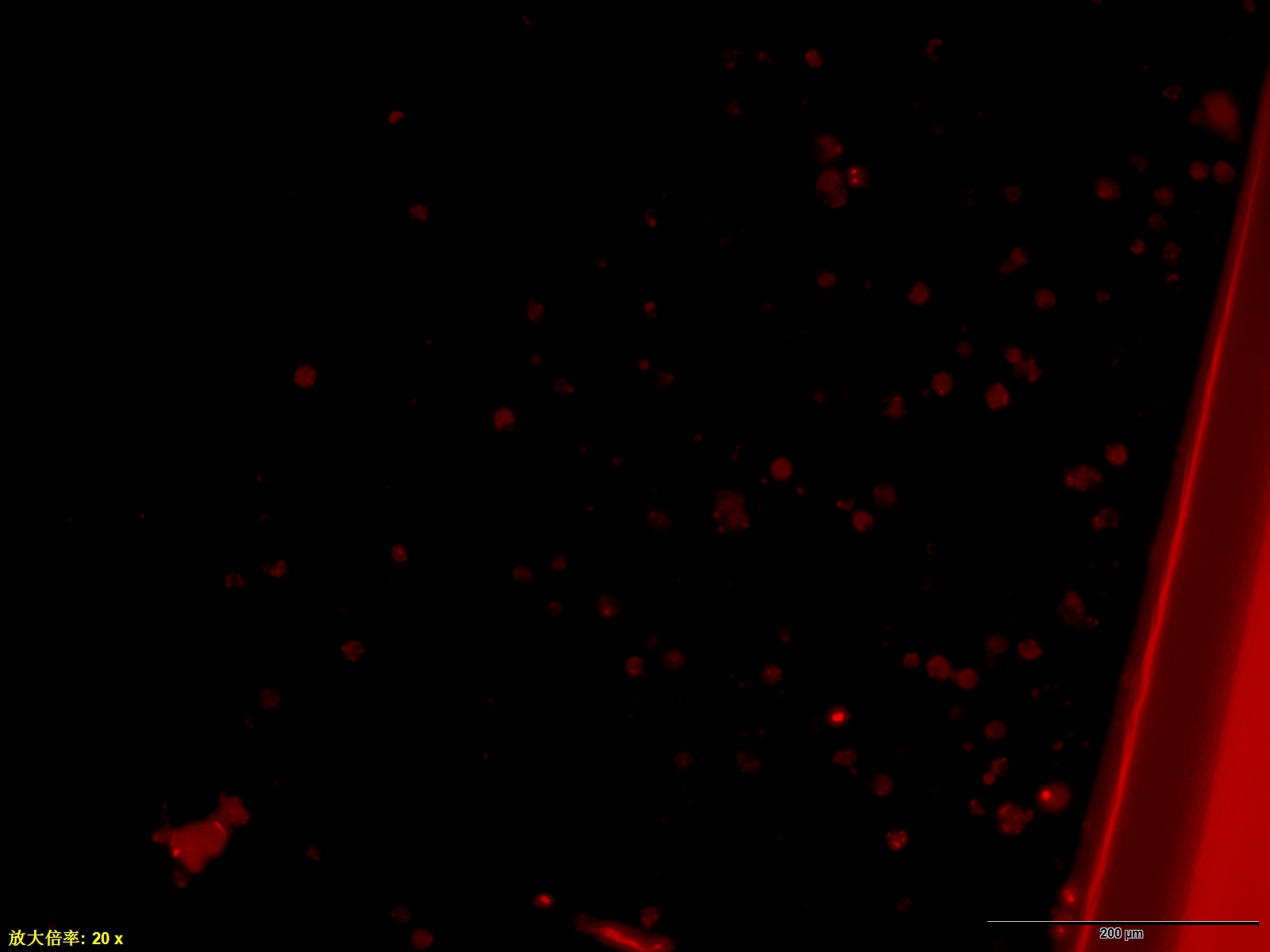

Supplement: Supplementary file 3 [file Data_Sheet_1.ZIP › cellular uptake-BMDC/RBD-Trimer/图像_21867.jpg]

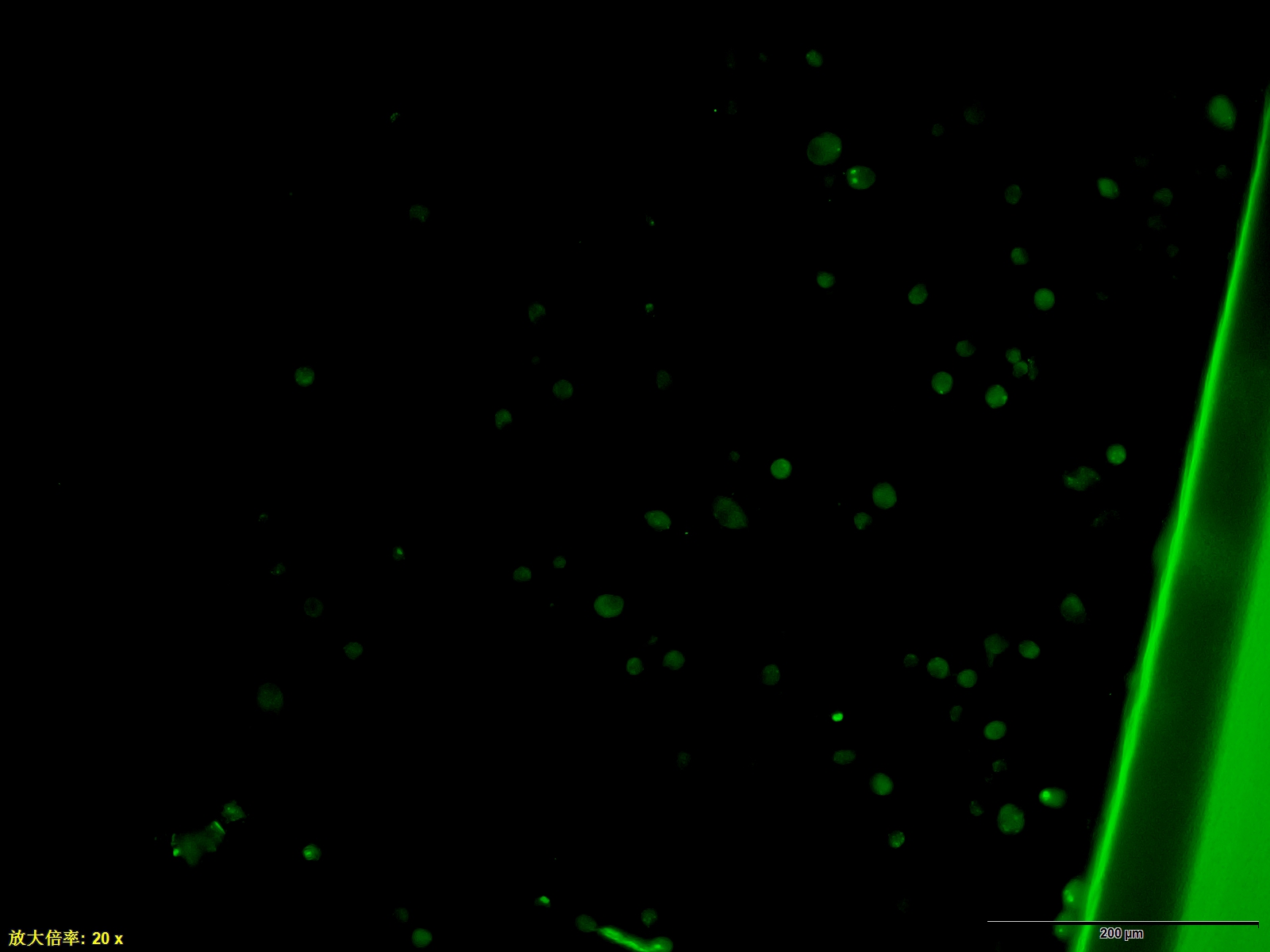

Supplement: Supplementary file 3 [file Data_Sheet_1.ZIP › cellular uptake-BMDC/RBD-Trimer/图像_21868.jpg]

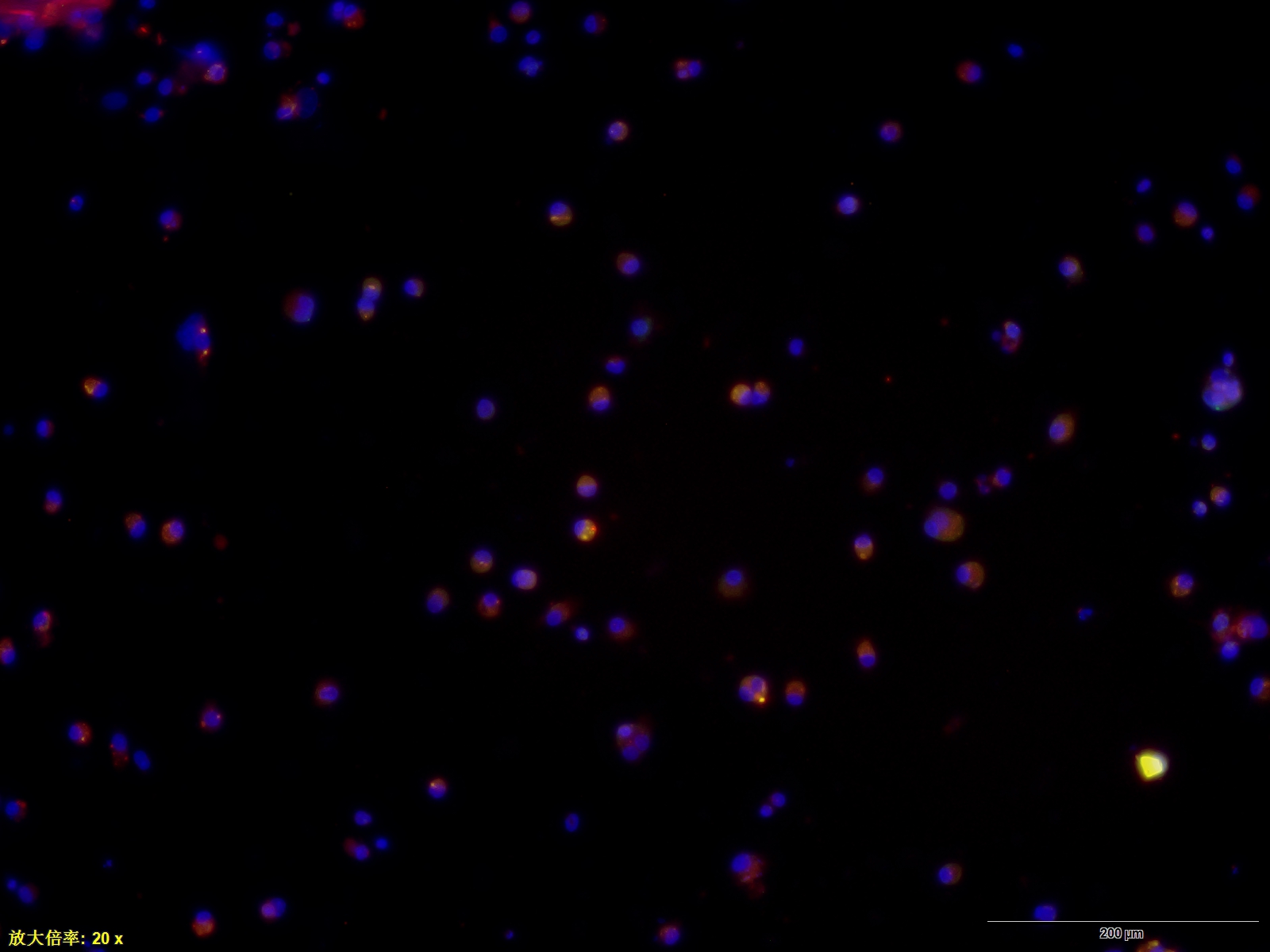

Supplement: Supplementary file 3 [file Data_Sheet_1.ZIP › cellular uptake-BMDC/S1-Monomer/图像_01.jpg]

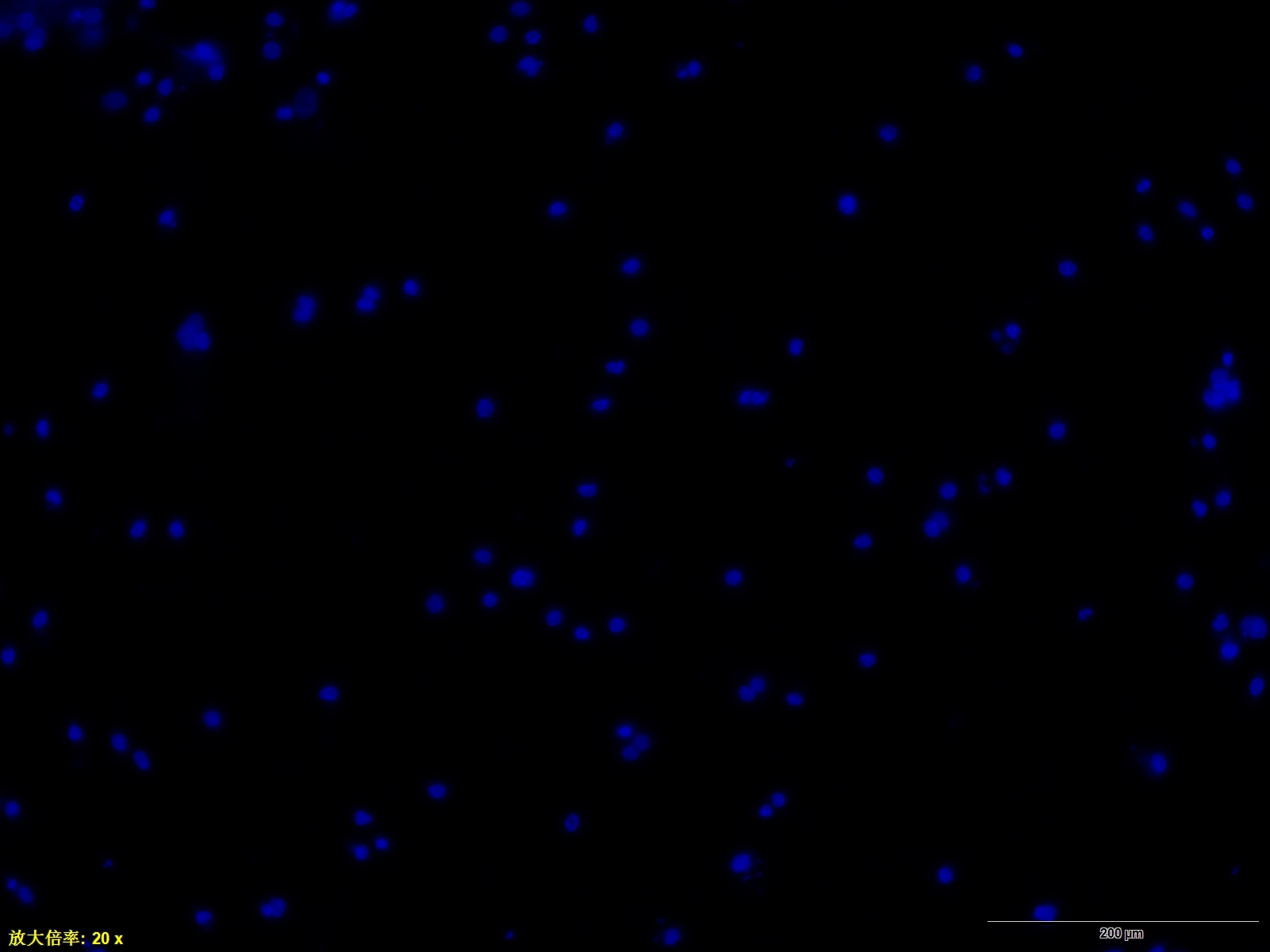

Supplement: Supplementary file 3 [file Data_Sheet_1.ZIP › cellular uptake-BMDC/S1-Monomer/图像_21856.jpg]

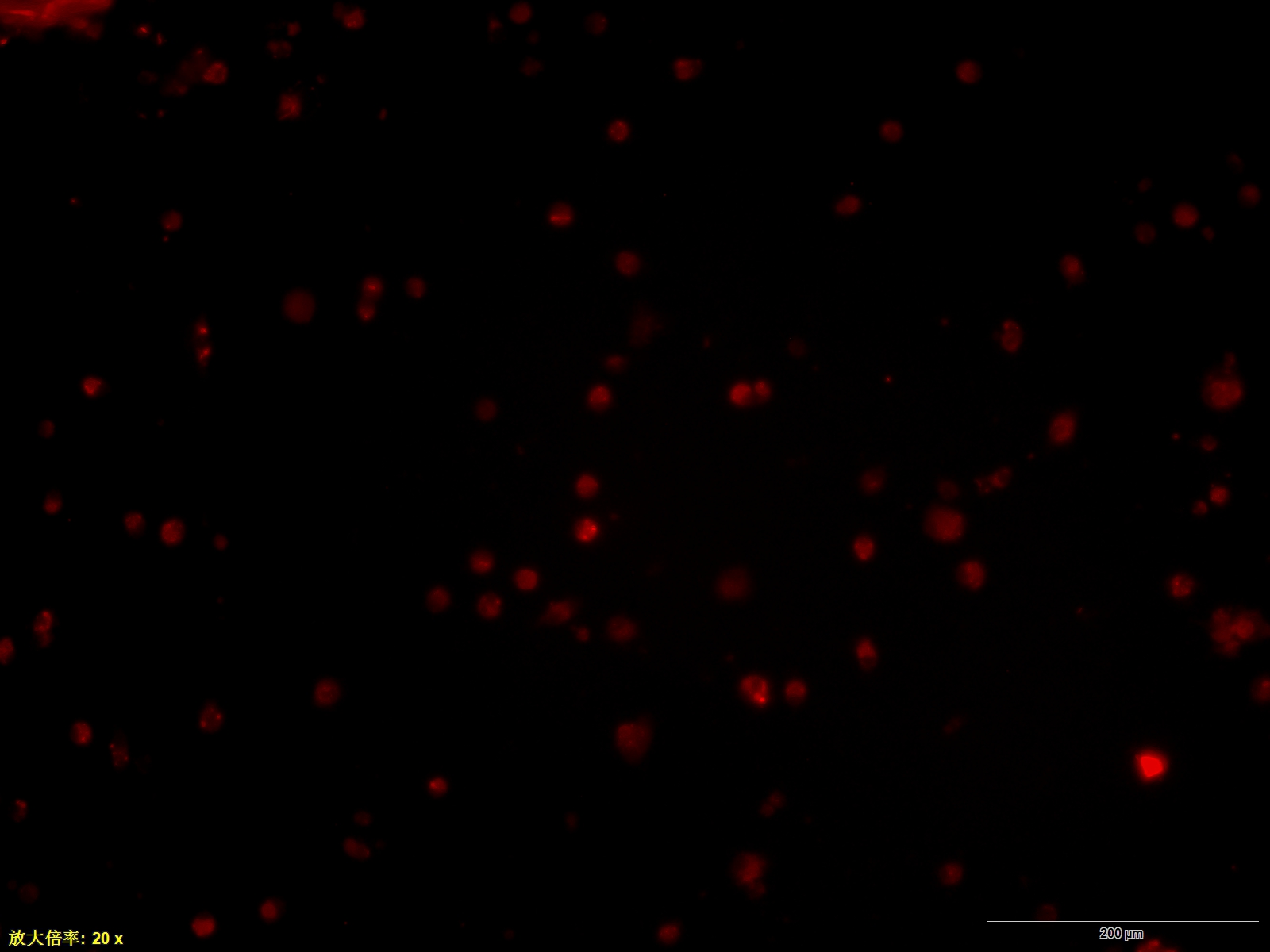

Supplement: Supplementary file 3 [file Data_Sheet_1.ZIP › cellular uptake-BMDC/S1-Monomer/图像_21857.jpg]

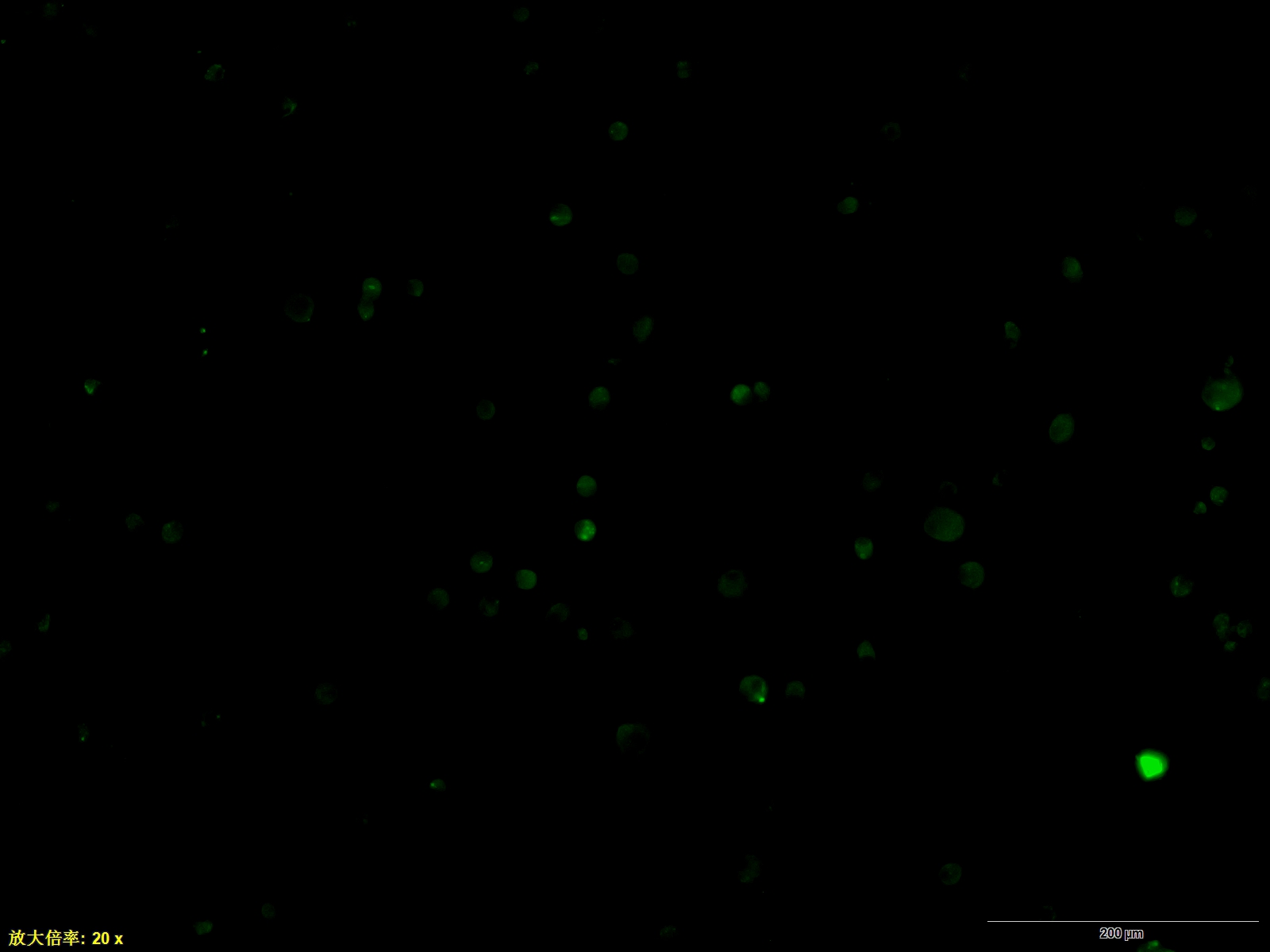

Supplement: Supplementary file 3 [file Data_Sheet_1.ZIP › cellular uptake-BMDC/S1-Monomer/图像_21858.jpg]

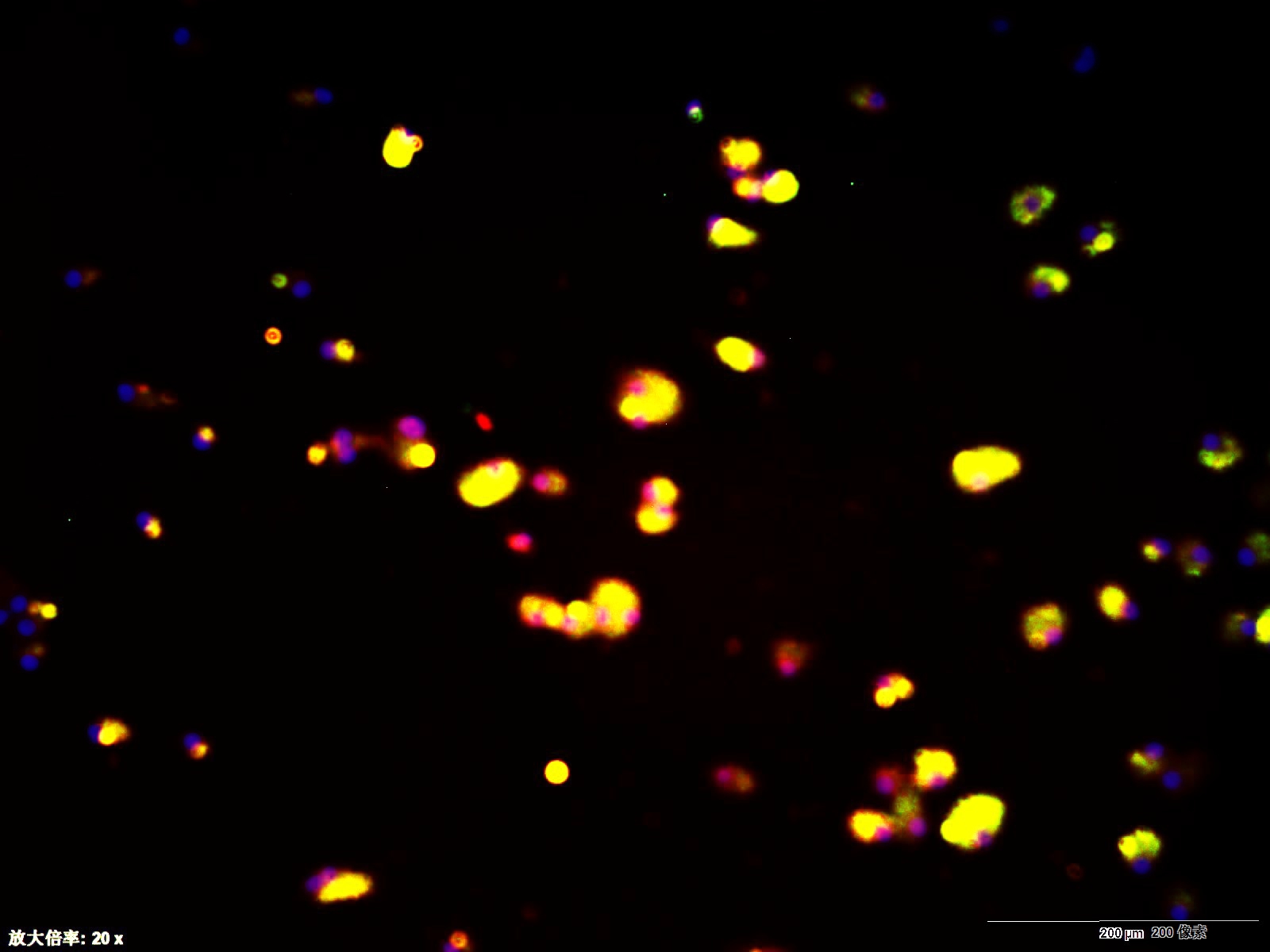

Supplement: Supplementary file 3 [file Data_Sheet_1.ZIP › cellular uptake-BMDC/S1-Trimer/图像_01.jpg]

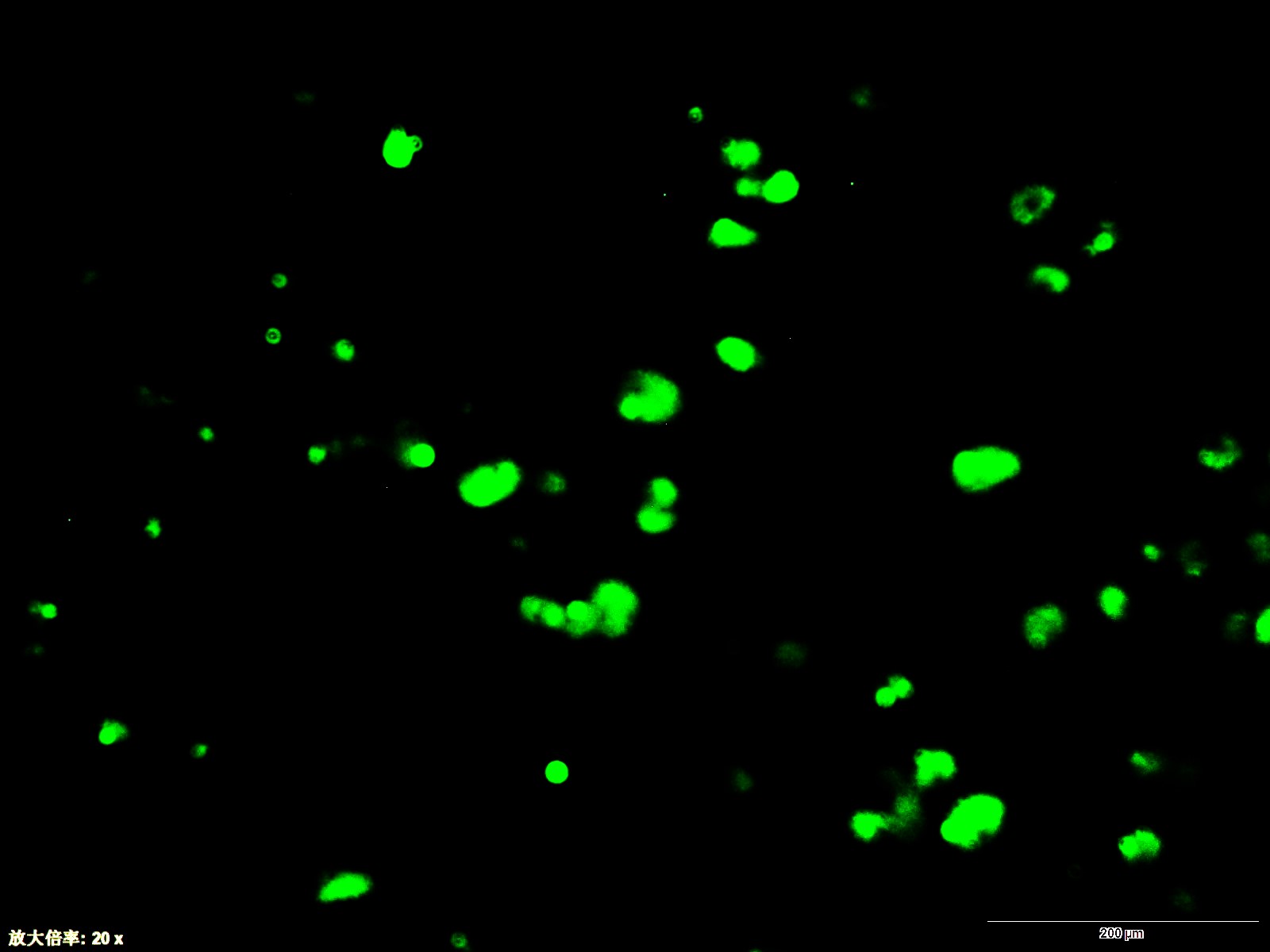

Supplement: Supplementary file 3 [file Data_Sheet_1.ZIP › cellular uptake-BMDC/S1-Trimer/图像_21863.jpg]

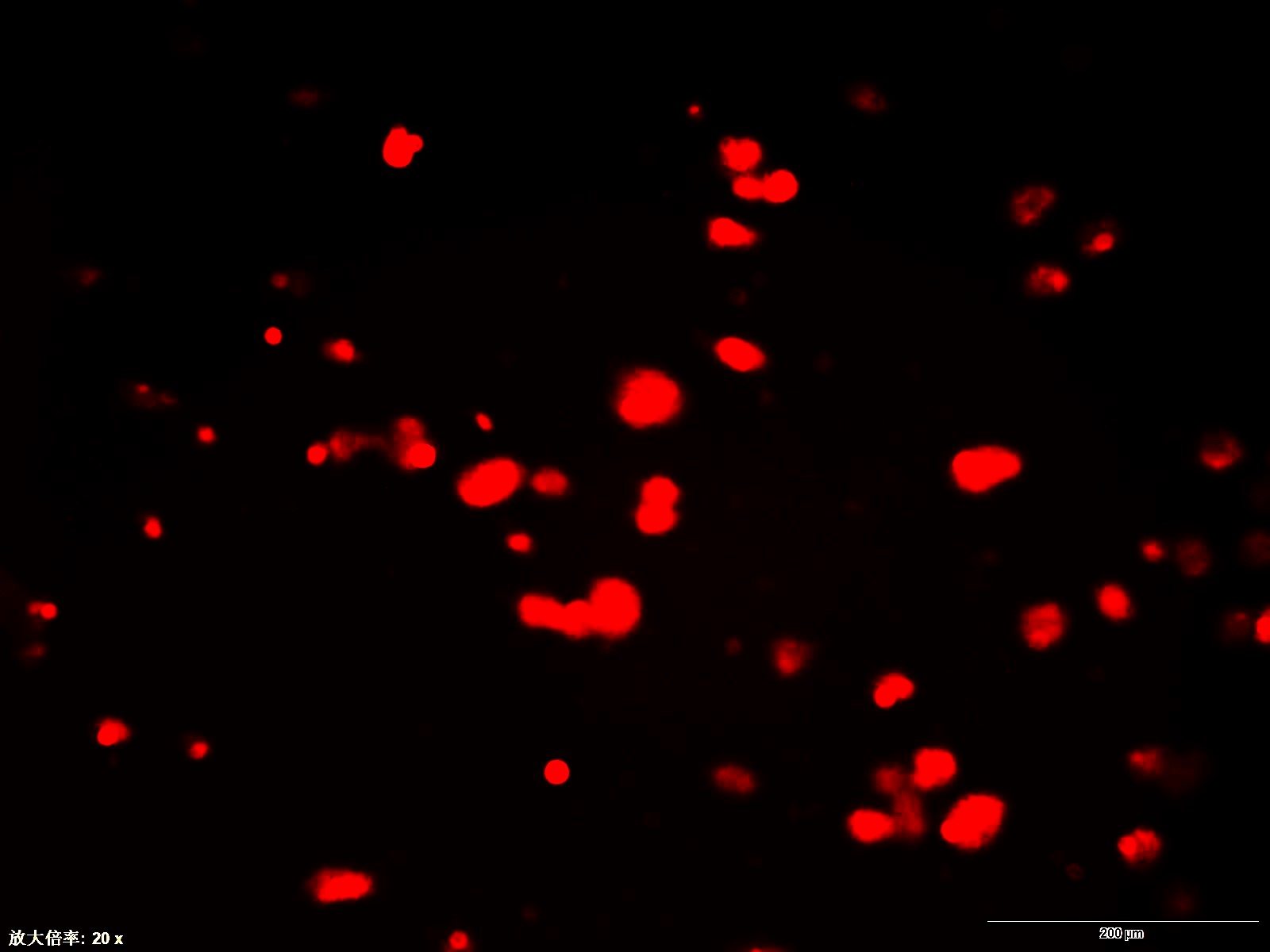

Supplement: Supplementary file 3 [file Data_Sheet_1.ZIP › cellular uptake-BMDC/S1-Trimer/图像_21864.jpg]

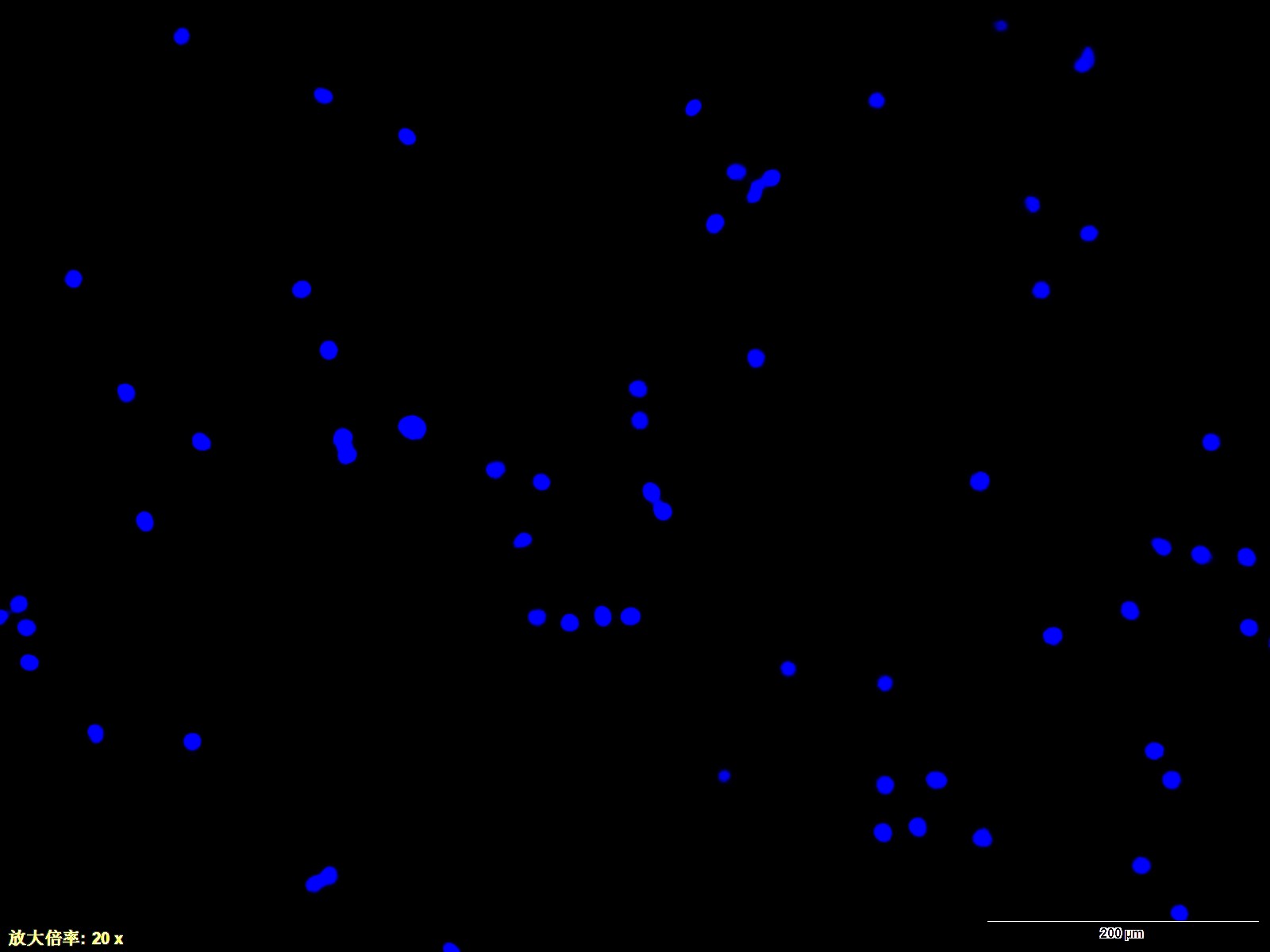

Supplement: Supplementary file 3 [file Data_Sheet_1.ZIP › cellular uptake-BMDC/S1-Trimer/图像_21865.jpg]

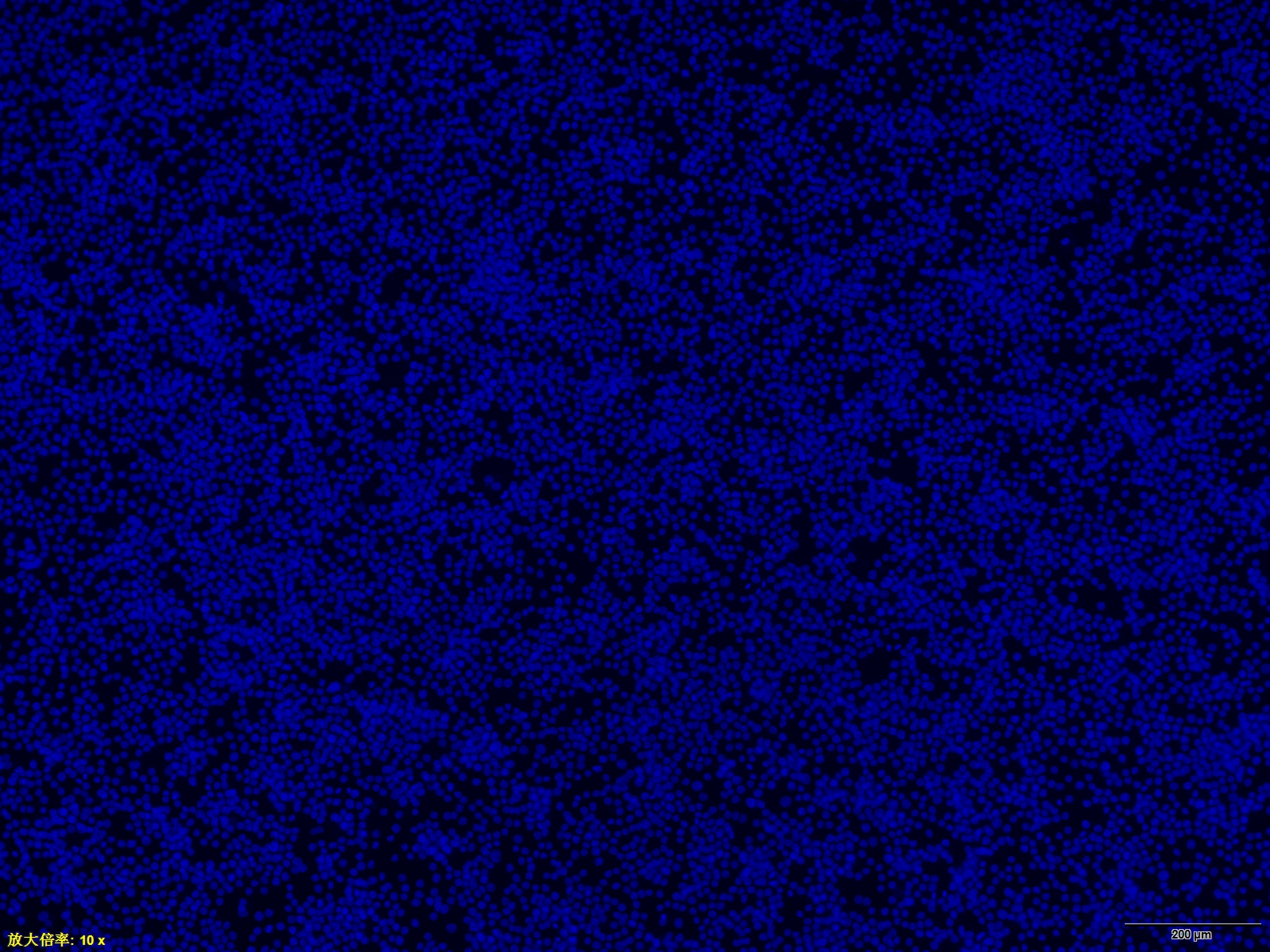

Supplement: Supplementary file 4 [file Data_Sheet_2.ZIP › cellular uptake-RAW.264.7/COE-Monomer/D.jpg]

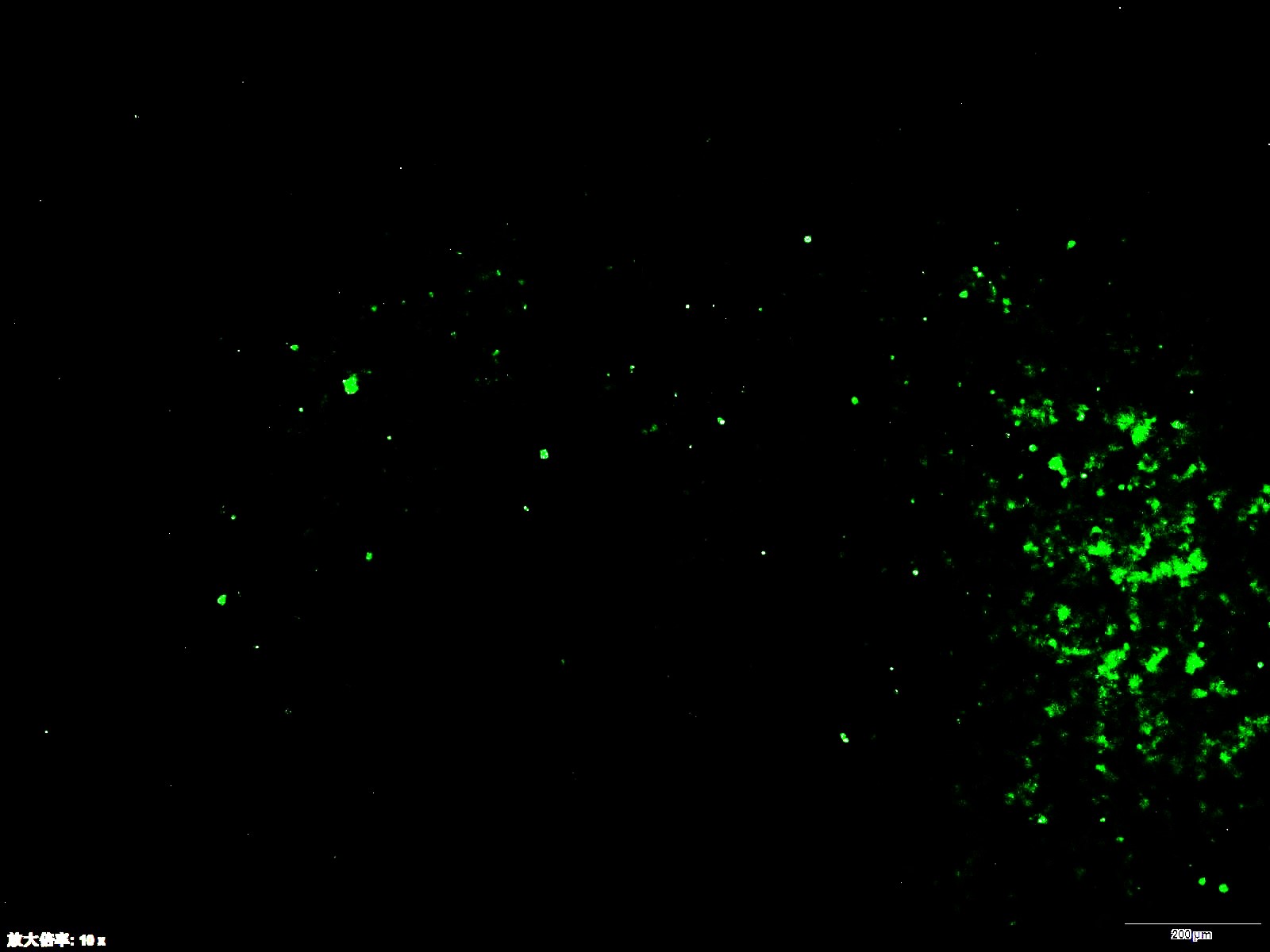

Supplement: Supplementary file 4 [file Data_Sheet_2.ZIP › cellular uptake-RAW.264.7/COE-Monomer/F.jpg]

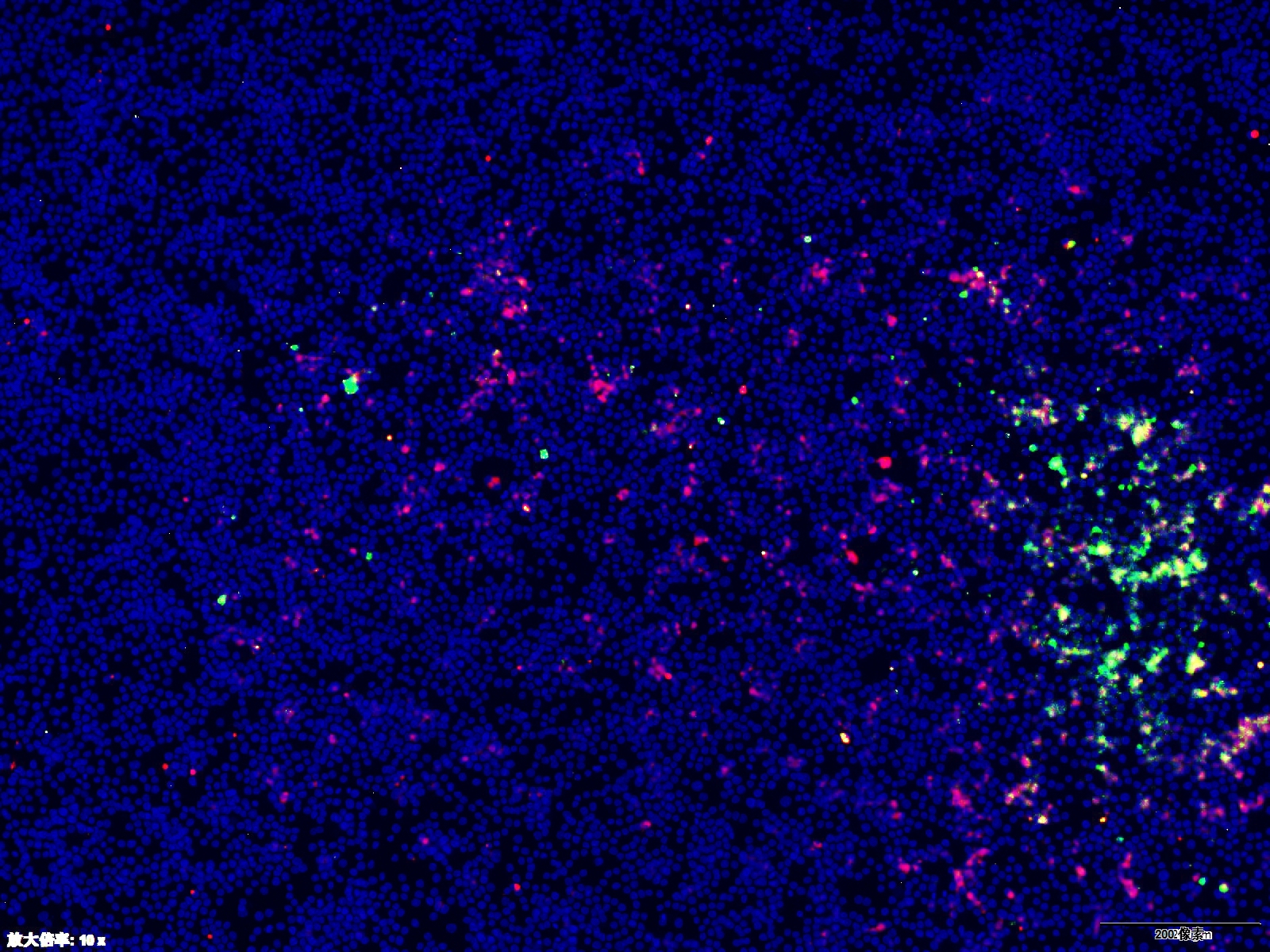

Supplement: Supplementary file 4 [file Data_Sheet_2.ZIP › cellular uptake-RAW.264.7/COE-Monomer/M.jpg]

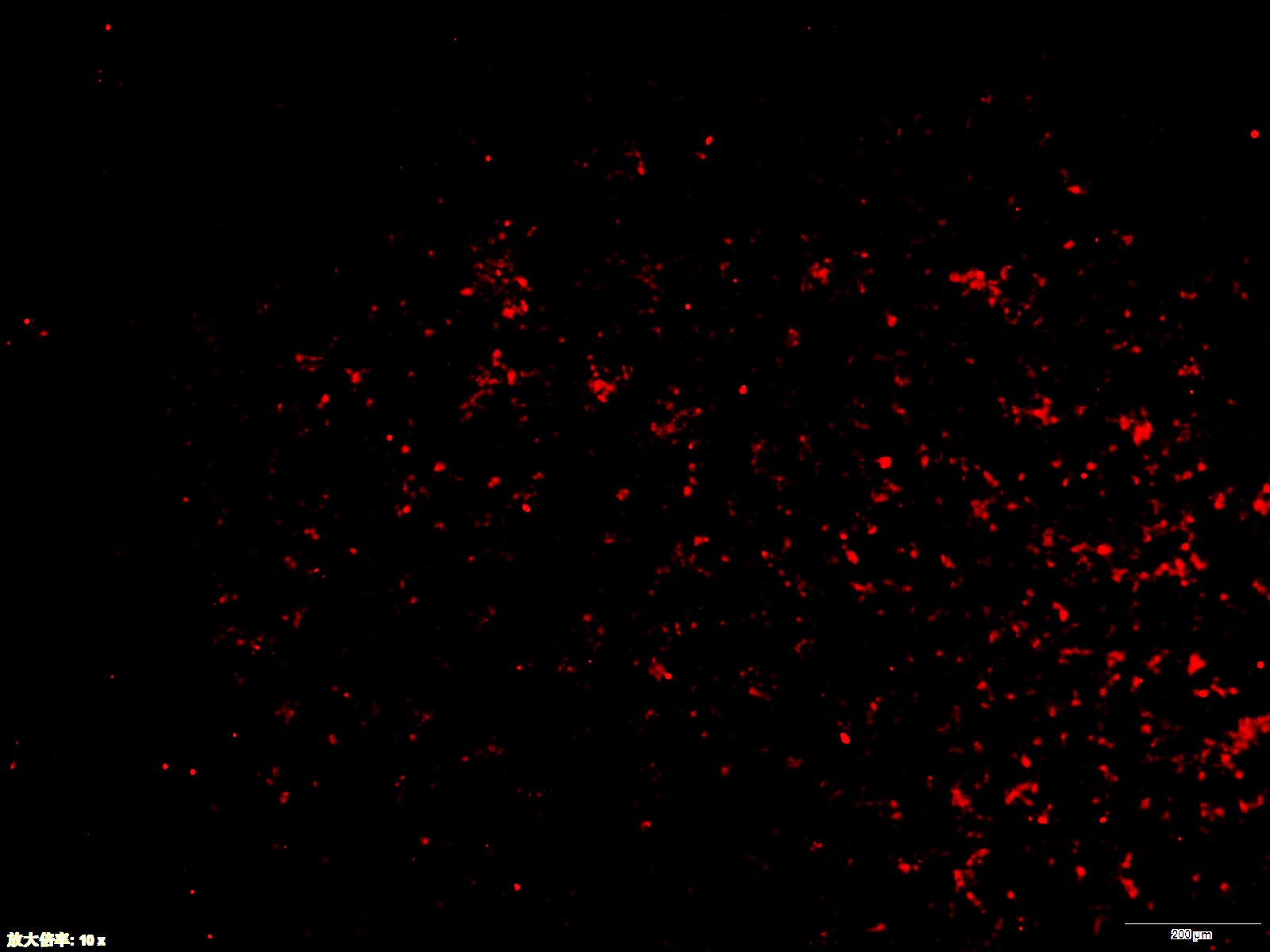

Supplement: Supplementary file 4 [file Data_Sheet_2.ZIP › cellular uptake-RAW.264.7/COE-Monomer/T.jpg]

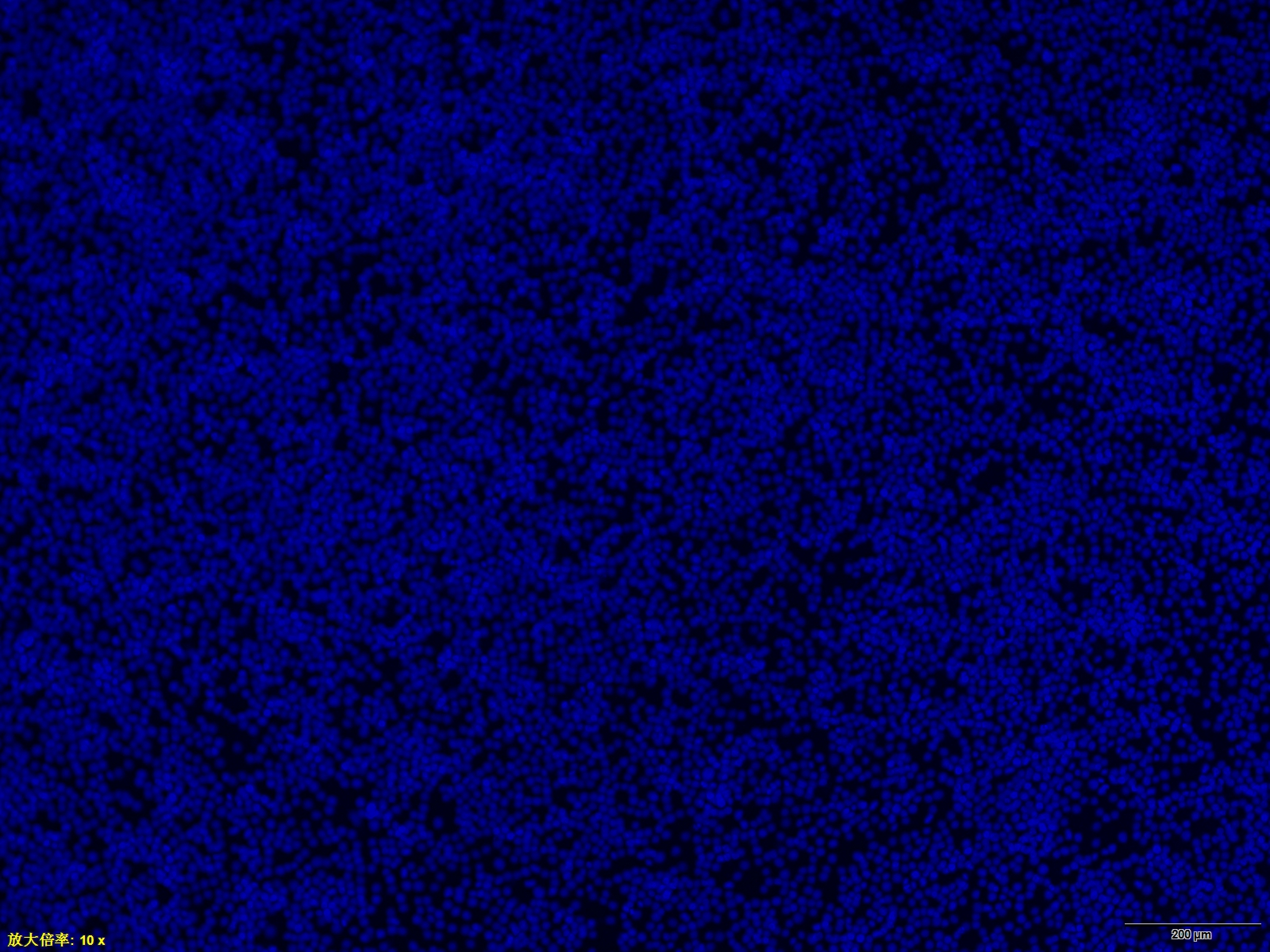

Supplement: Supplementary file 4 [file Data_Sheet_2.ZIP › cellular uptake-RAW.264.7/COE-Trimer/D.jpg]

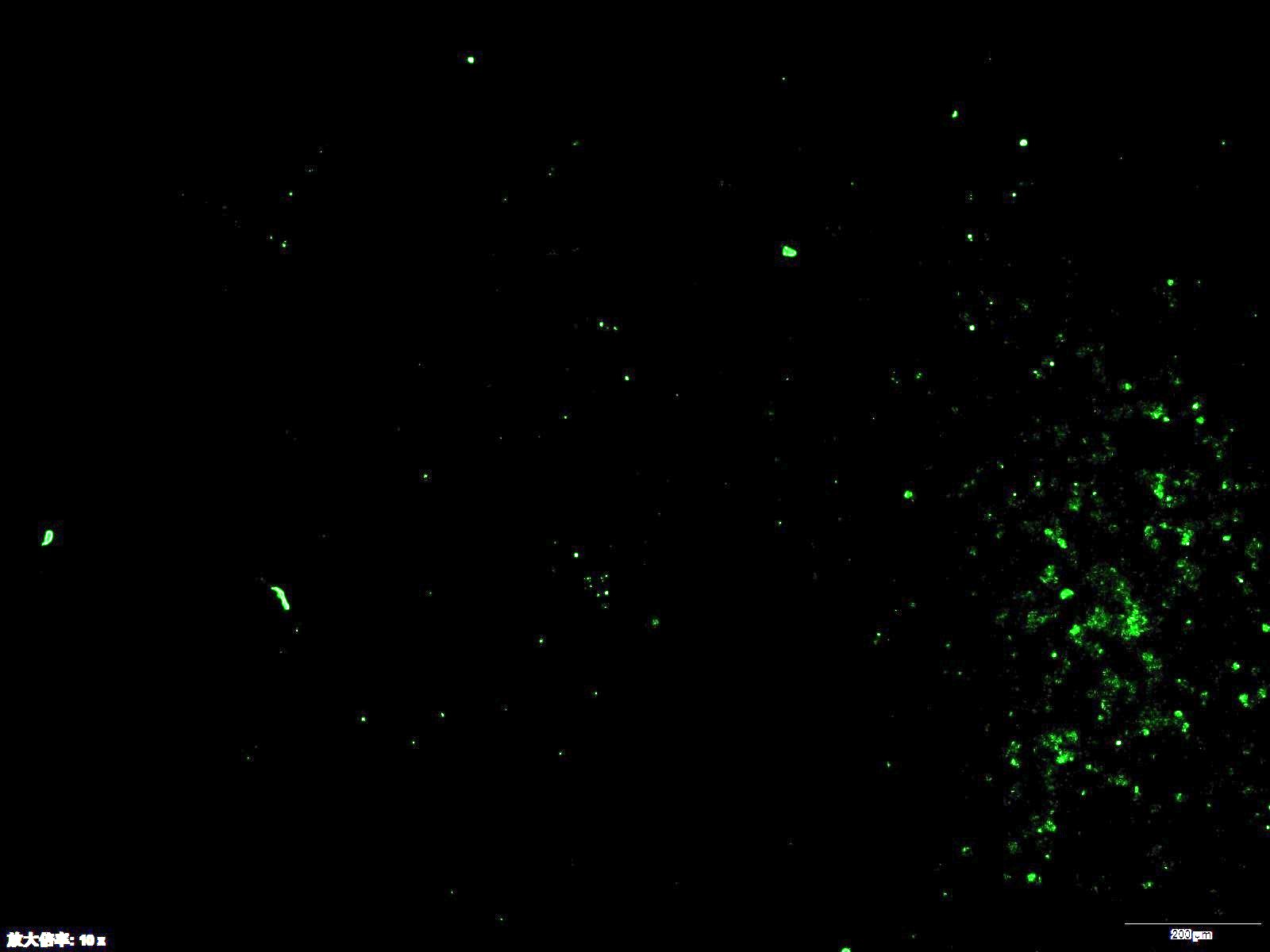

Supplement: Supplementary file 4 [file Data_Sheet_2.ZIP › cellular uptake-RAW.264.7/COE-Trimer/F.jpg]

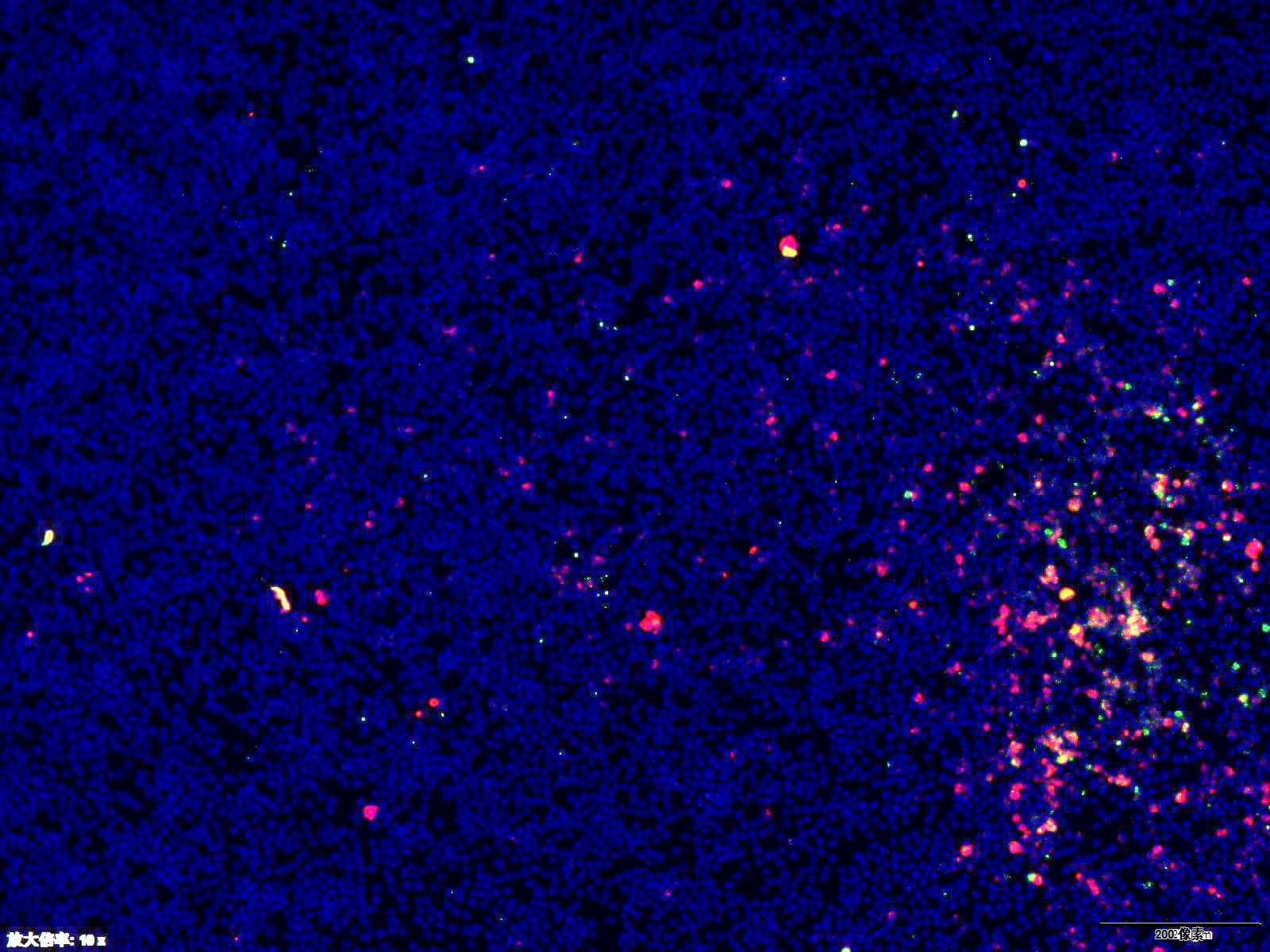

Supplement: Supplementary file 4 [file Data_Sheet_2.ZIP › cellular uptake-RAW.264.7/COE-Trimer/M.jpg]

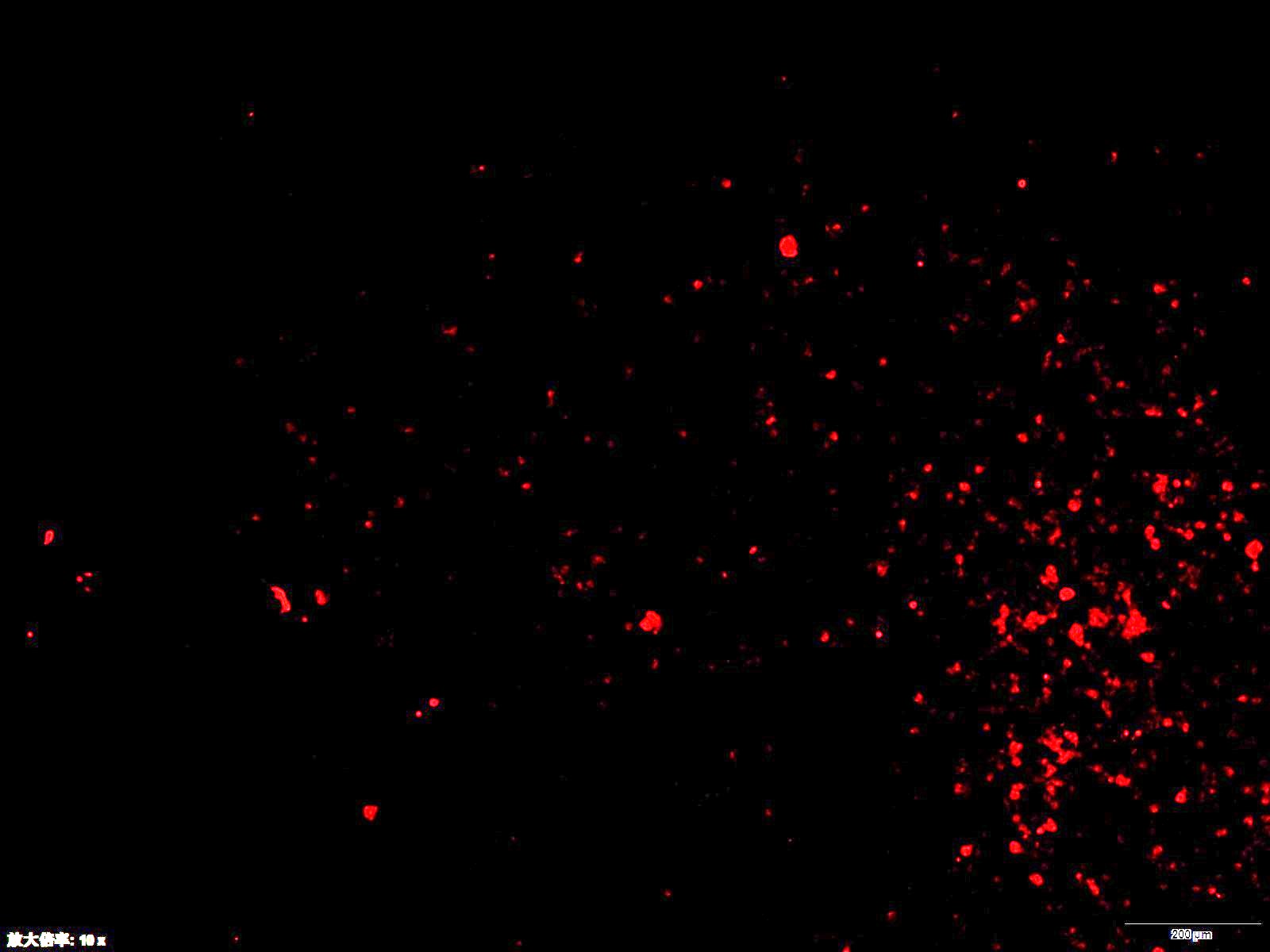

Supplement: Supplementary file 4 [file Data_Sheet_2.ZIP › cellular uptake-RAW.264.7/COE-Trimer/T.jpg]

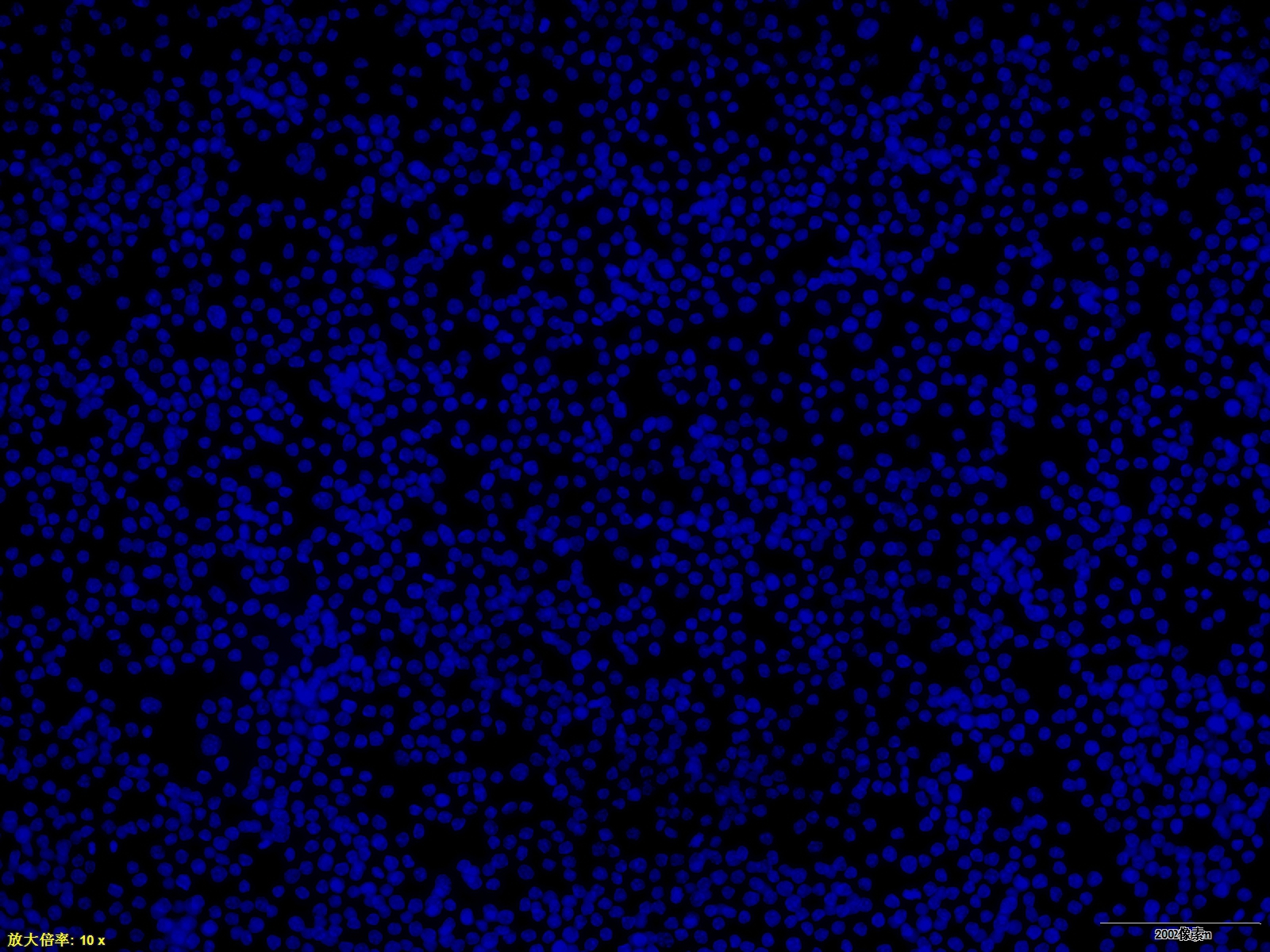

Supplement: Supplementary file 4 [file Data_Sheet_2.ZIP › cellular uptake-RAW.264.7/RBD-Monomer/D.jpg]

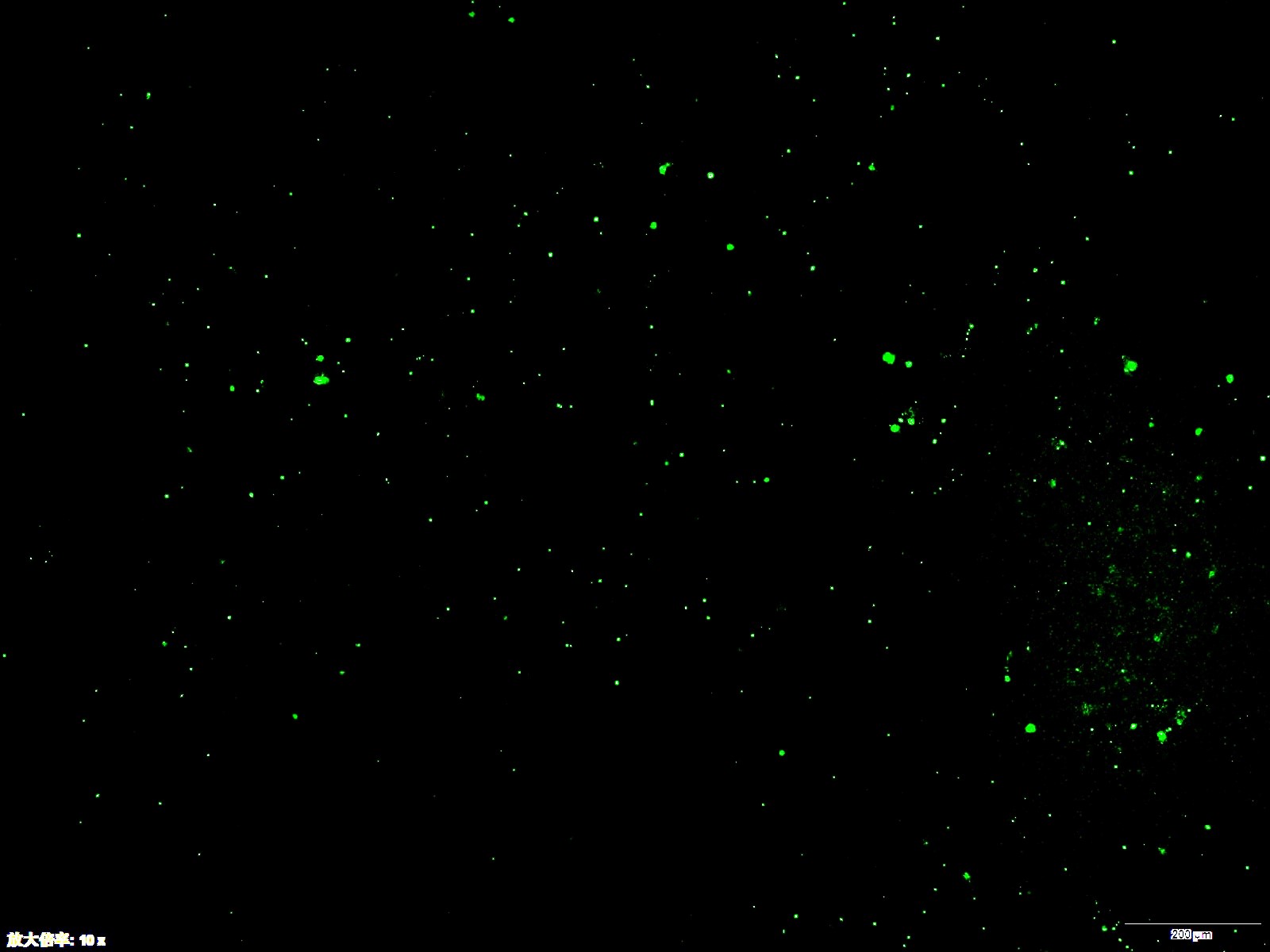

Supplement: Supplementary file 4 [file Data_Sheet_2.ZIP › cellular uptake-RAW.264.7/RBD-Monomer/F.jpg]

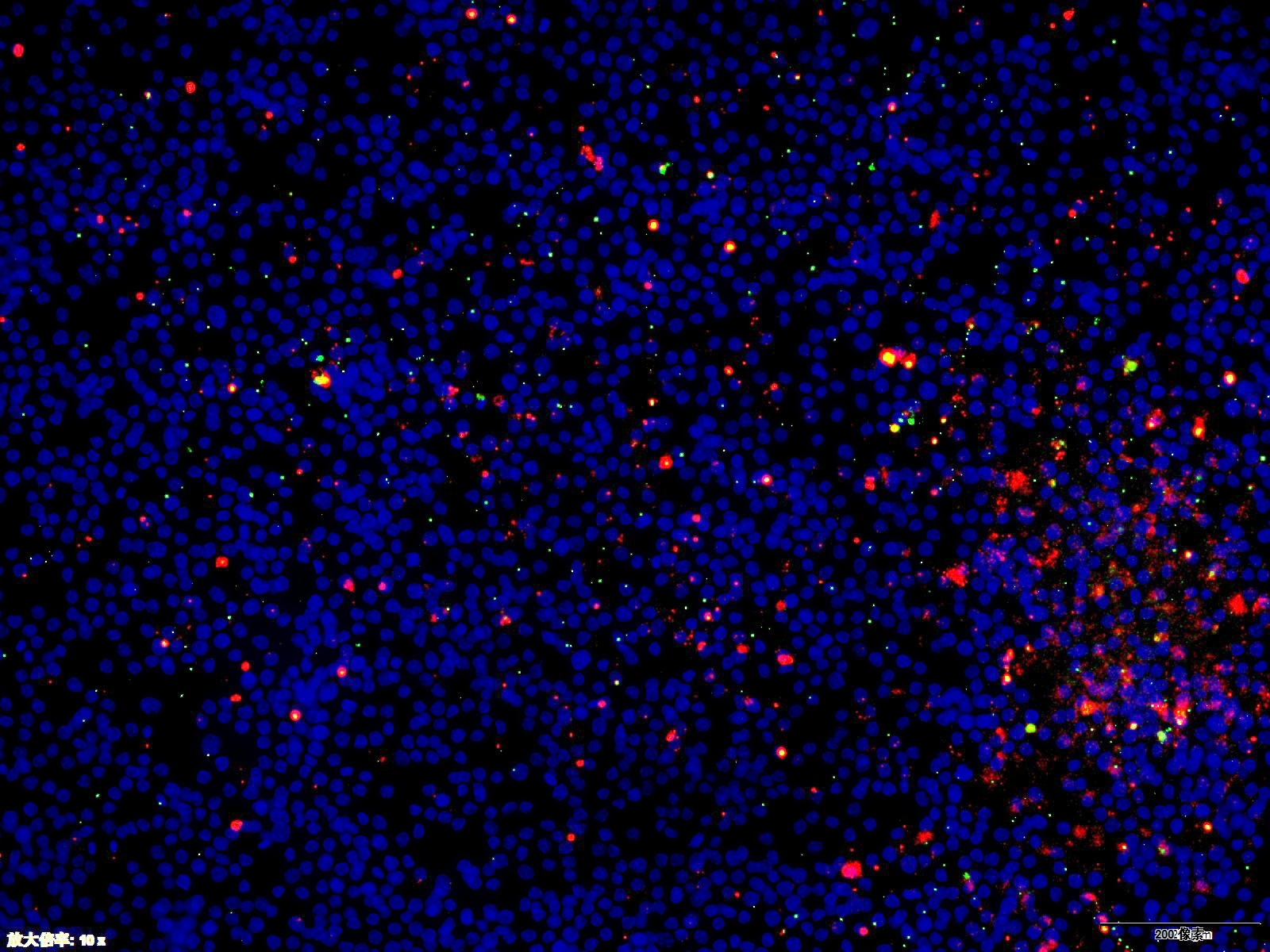

Supplement: Supplementary file 4 [file Data_Sheet_2.ZIP › cellular uptake-RAW.264.7/RBD-Monomer/M.jpg]

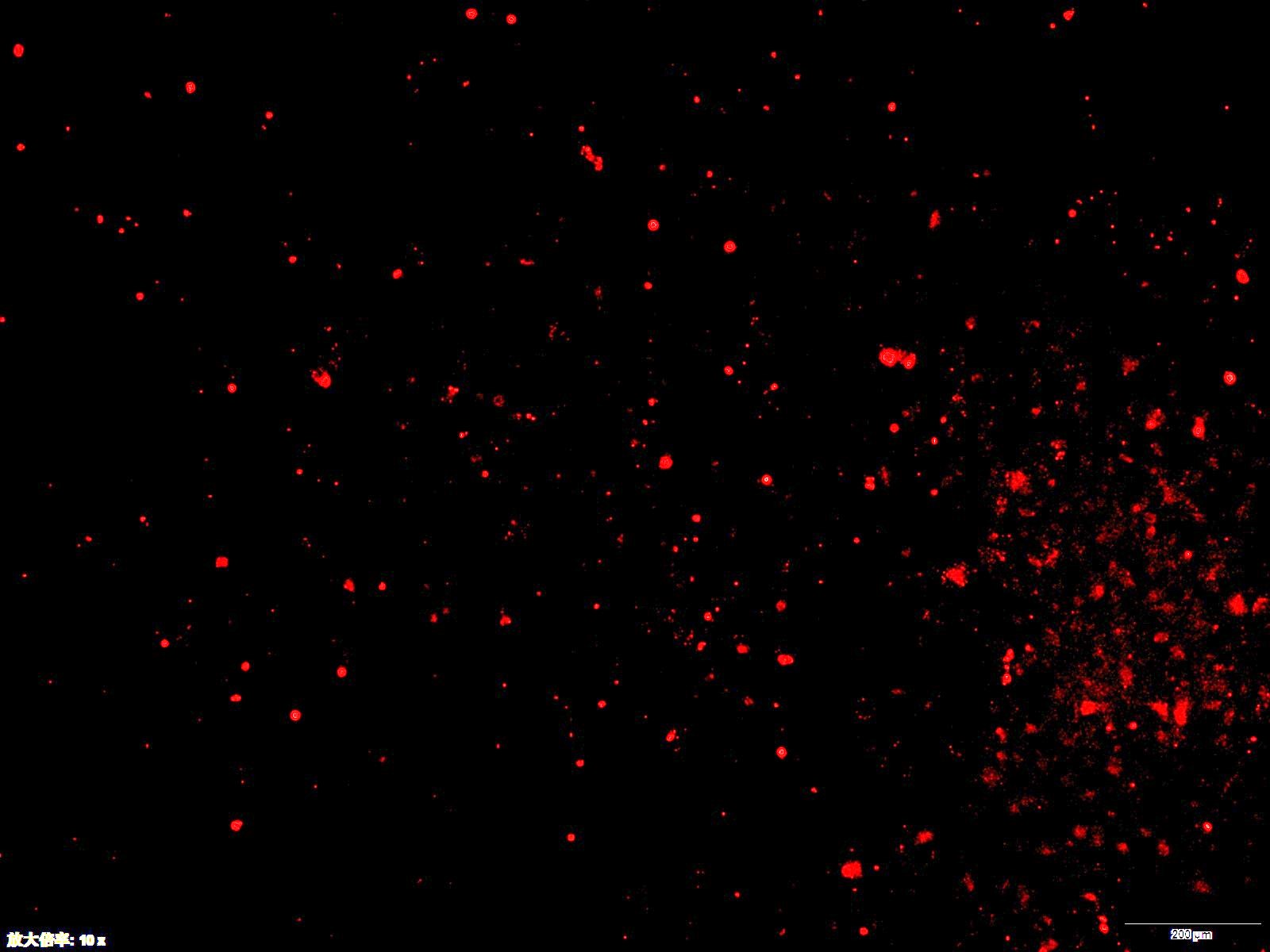

Supplement: Supplementary file 4 [file Data_Sheet_2.ZIP › cellular uptake-RAW.264.7/RBD-Monomer/T.jpg]

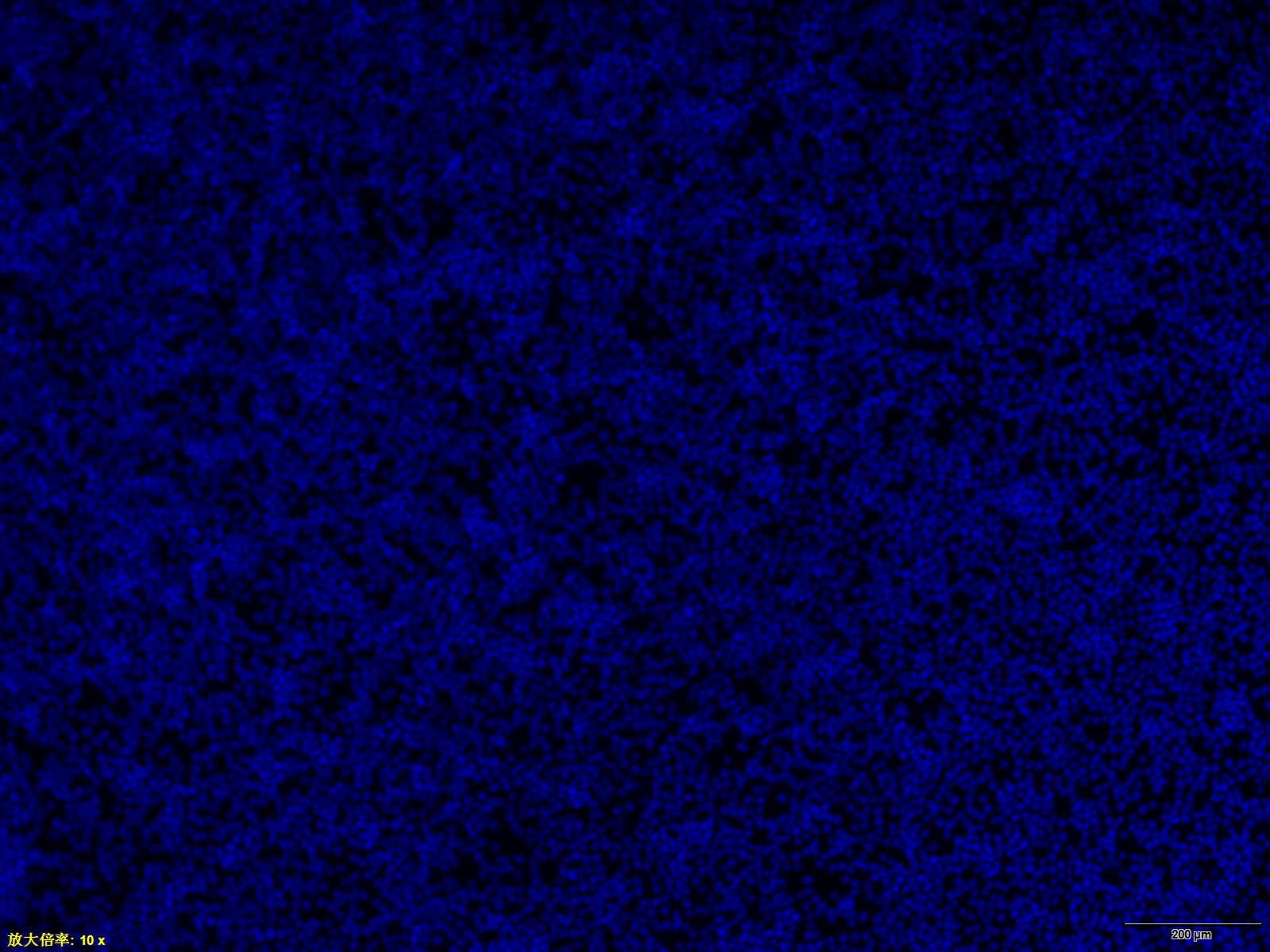

Supplement: Supplementary file 4 [file Data_Sheet_2.ZIP › cellular uptake-RAW.264.7/RBD-Trimer/D.jpg]

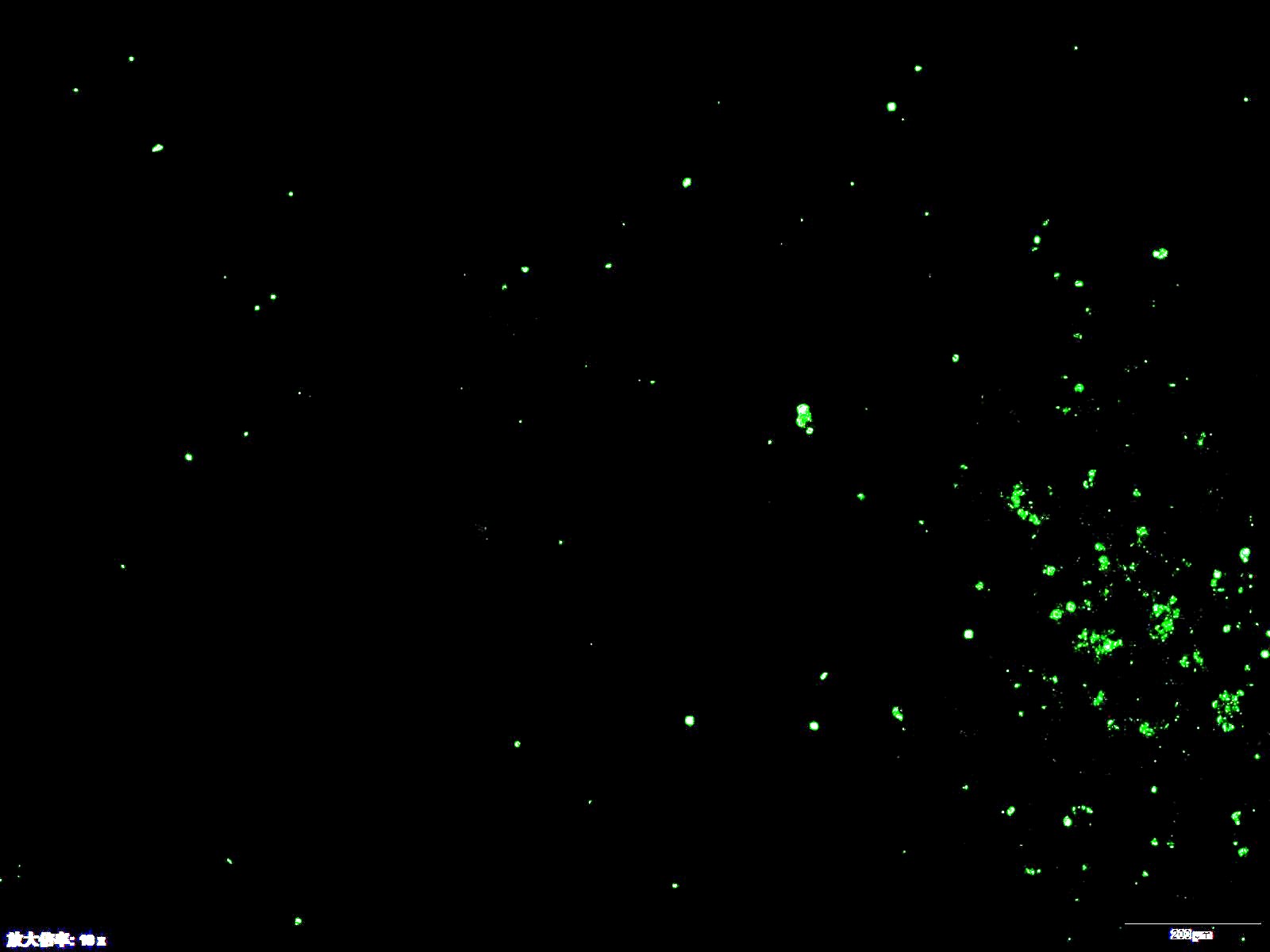

Supplement: Supplementary file 4 [file Data_Sheet_2.ZIP › cellular uptake-RAW.264.7/RBD-Trimer/F.jpg]

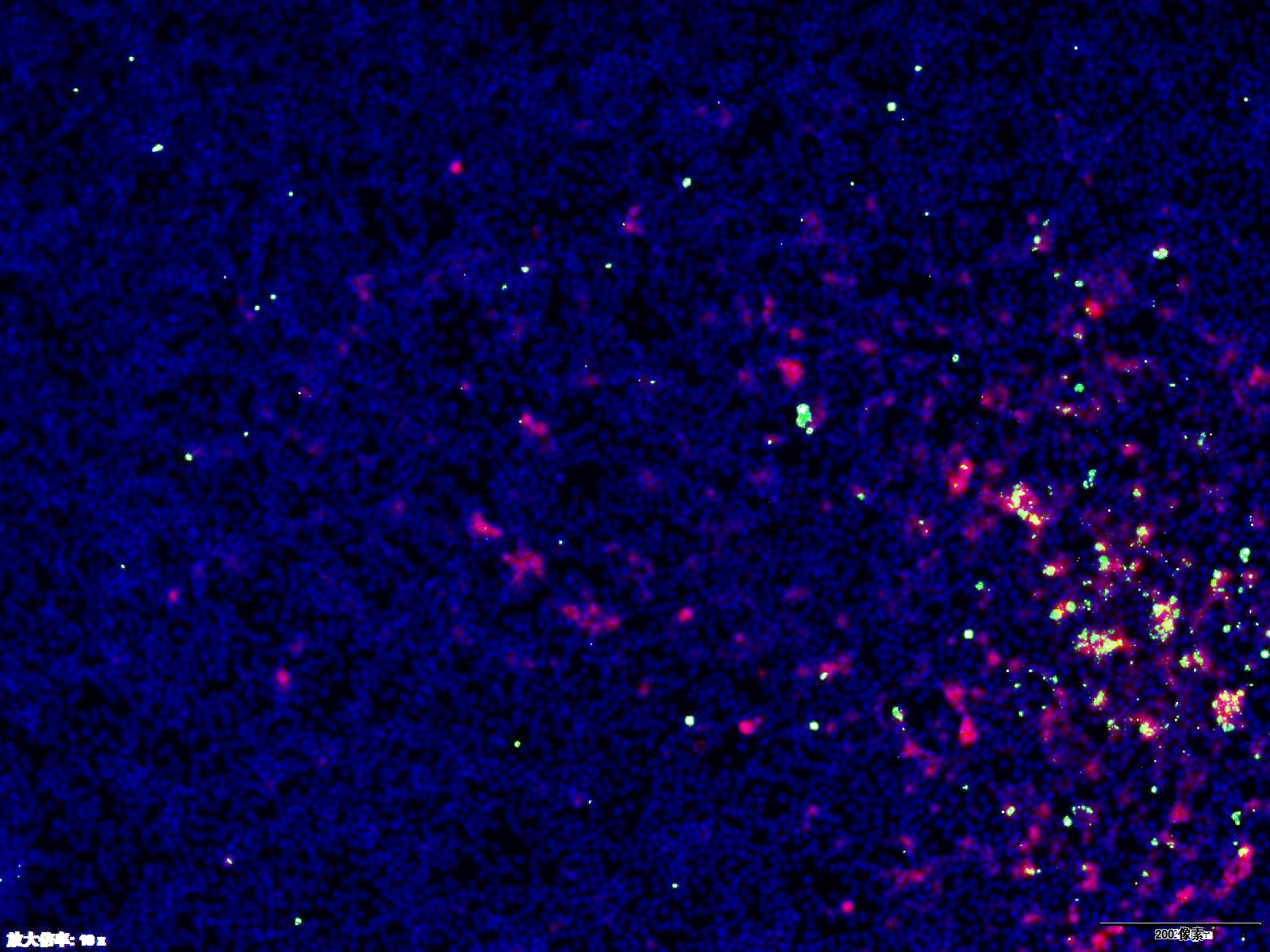

Supplement: Supplementary file 4 [file Data_Sheet_2.ZIP › cellular uptake-RAW.264.7/RBD-Trimer/M.jpg]

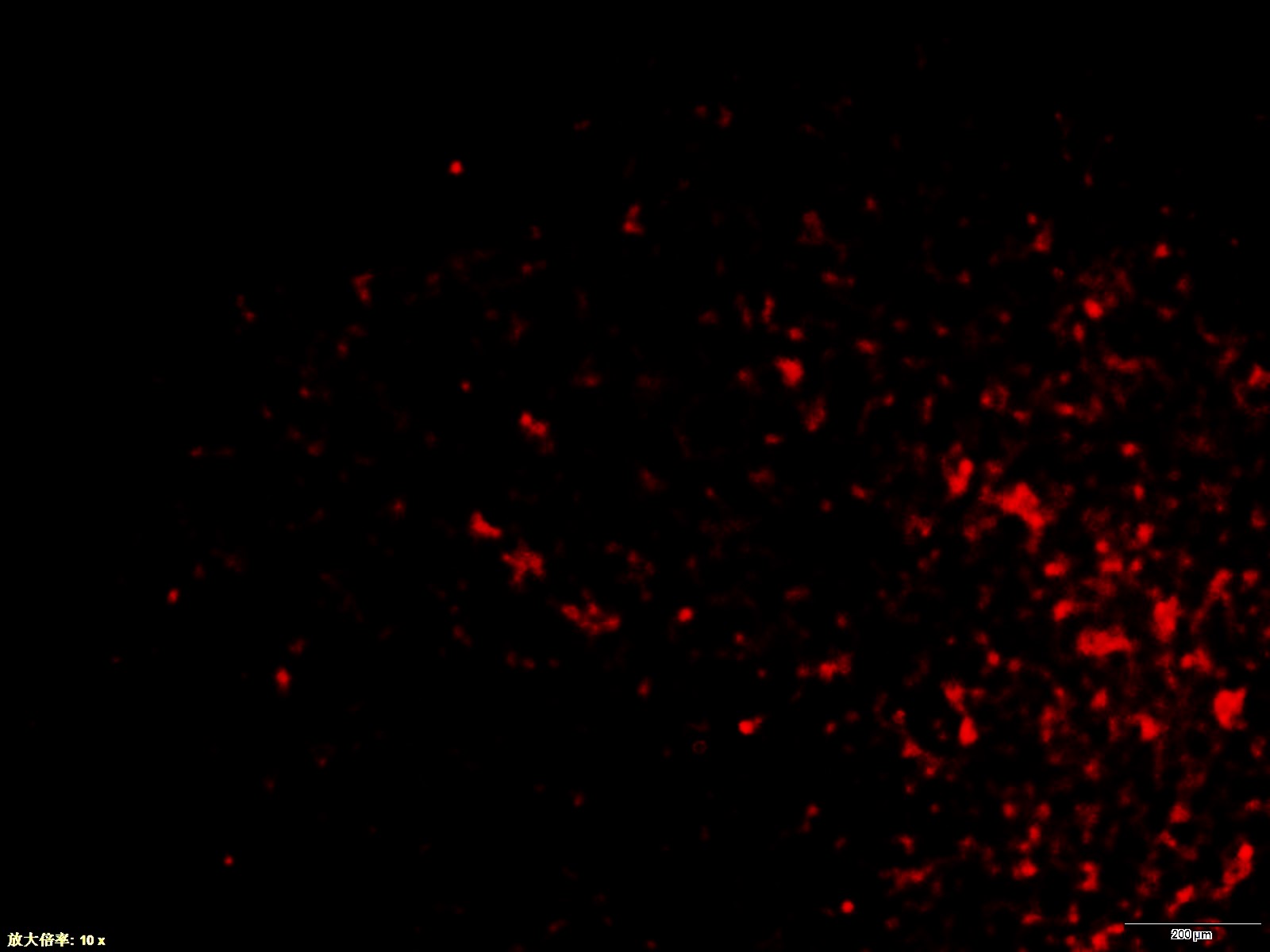

Supplement: Supplementary file 4 [file Data_Sheet_2.ZIP › cellular uptake-RAW.264.7/RBD-Trimer/T.jpg]

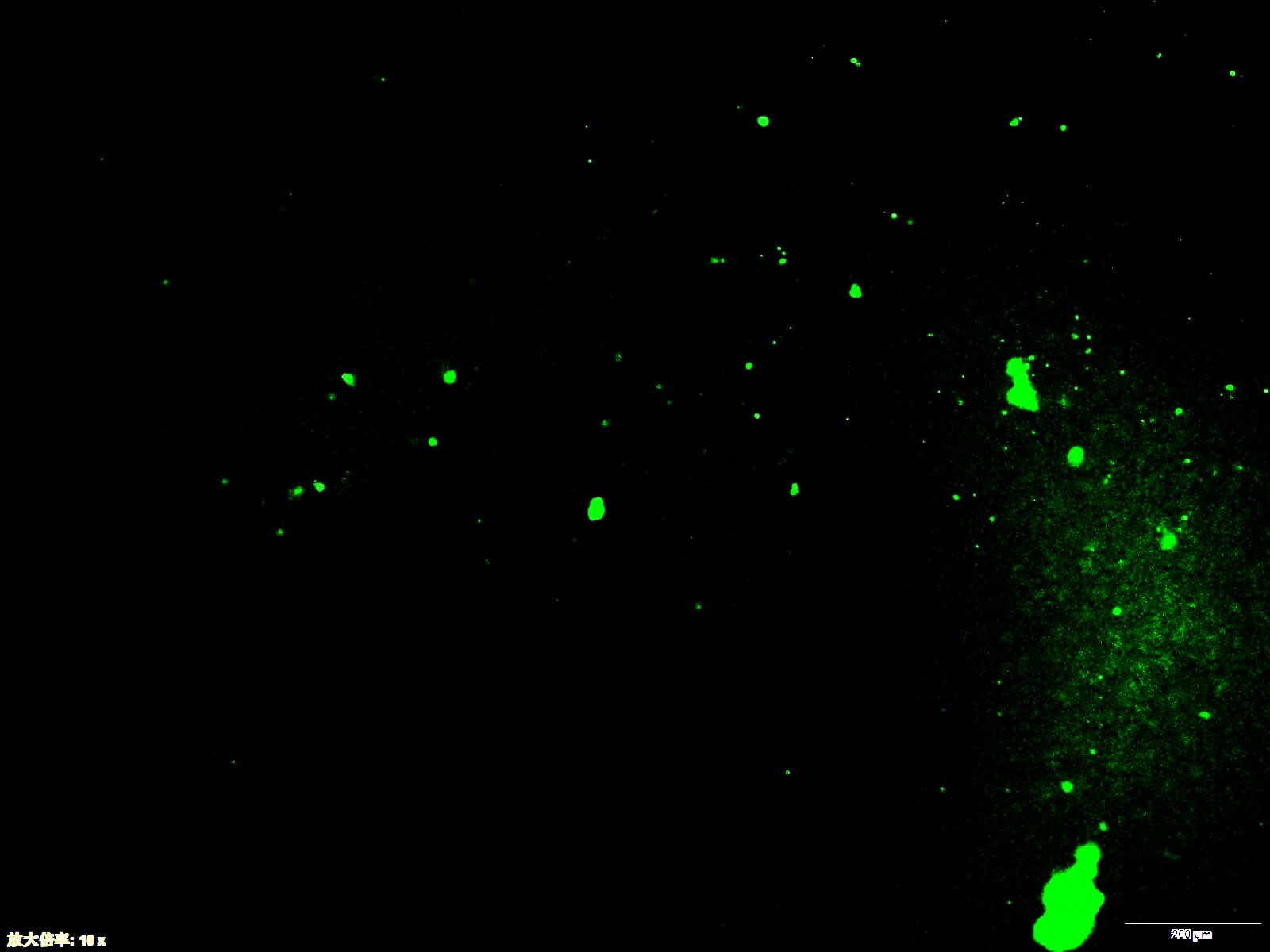

Supplement: Supplementary file 4 [file Data_Sheet_2.ZIP › cellular uptake-RAW.264.7/S1-Monomer/F.jpg]

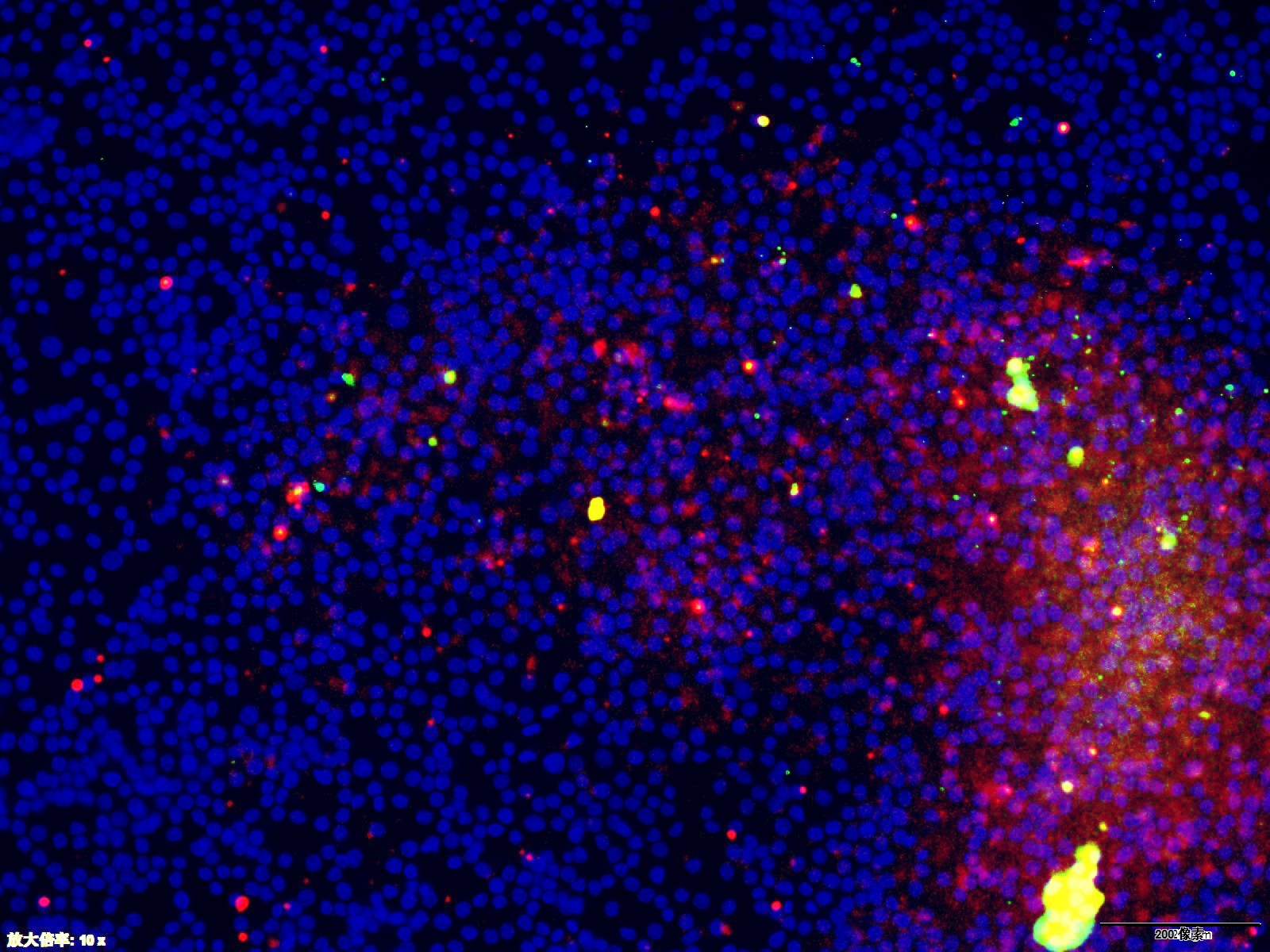

Supplement: Supplementary file 4 [file Data_Sheet_2.ZIP › cellular uptake-RAW.264.7/S1-Monomer/M.jpg]

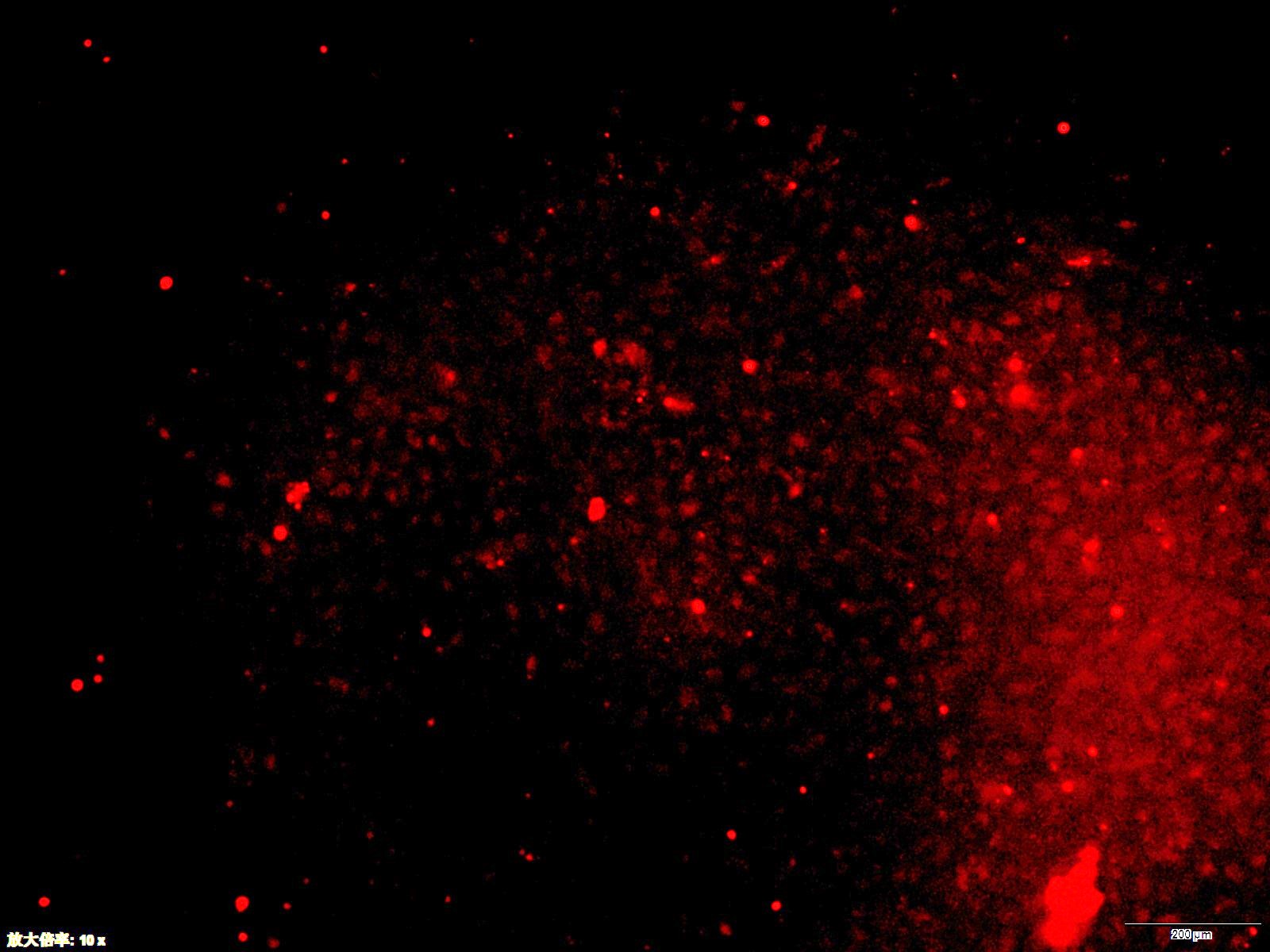

Supplement: Supplementary file 4 [file Data_Sheet_2.ZIP › cellular uptake-RAW.264.7/S1-Monomer/T.jpg]

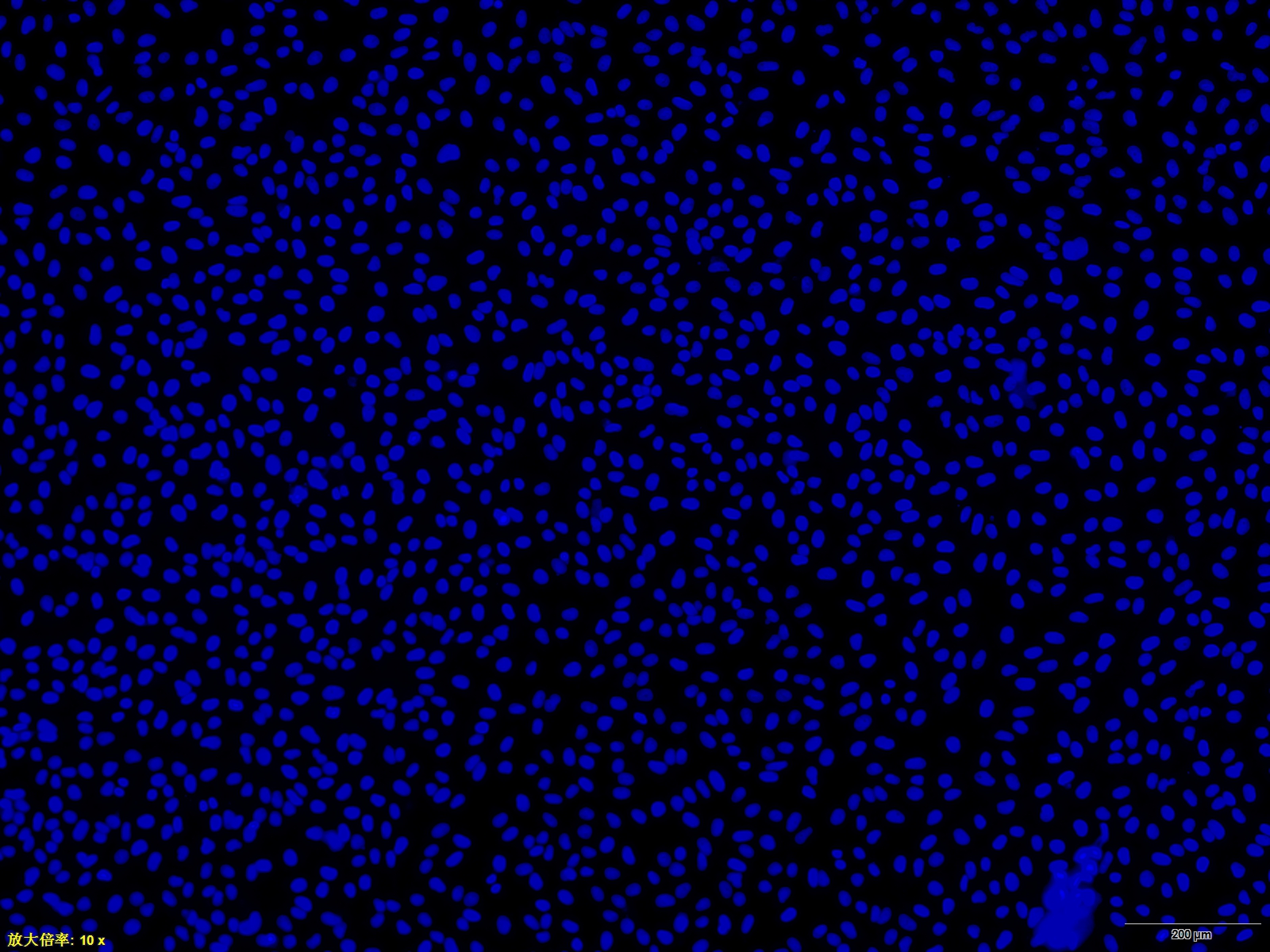

Supplement: Supplementary file 4 [file Data_Sheet_2.ZIP › cellular uptake-RAW.264.7/S1-Monomer/图像_21530.jpg]

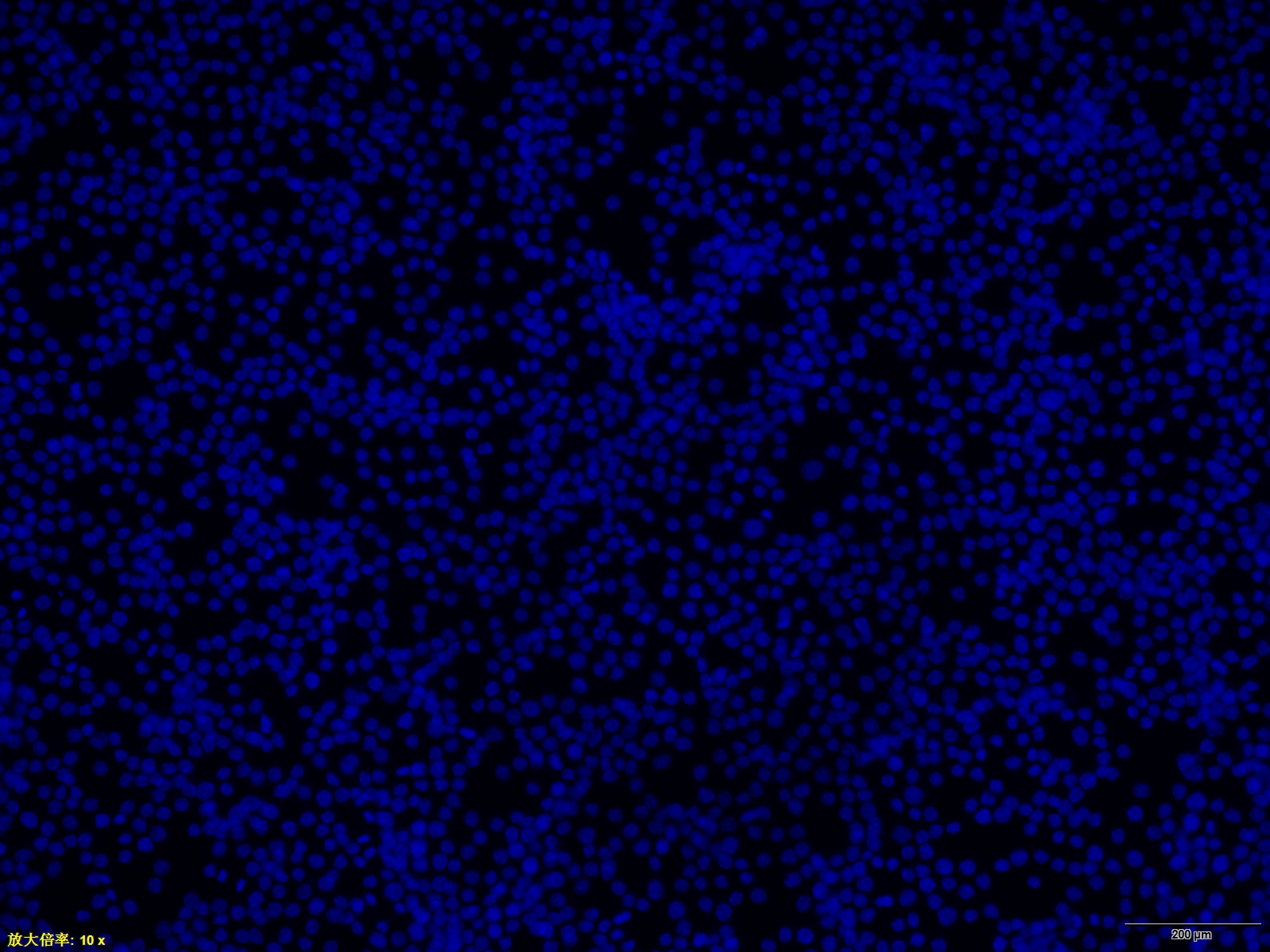

Supplement: Supplementary file 4 [file Data_Sheet_2.ZIP › cellular uptake-RAW.264.7/S1-Trimer/D.jpg]

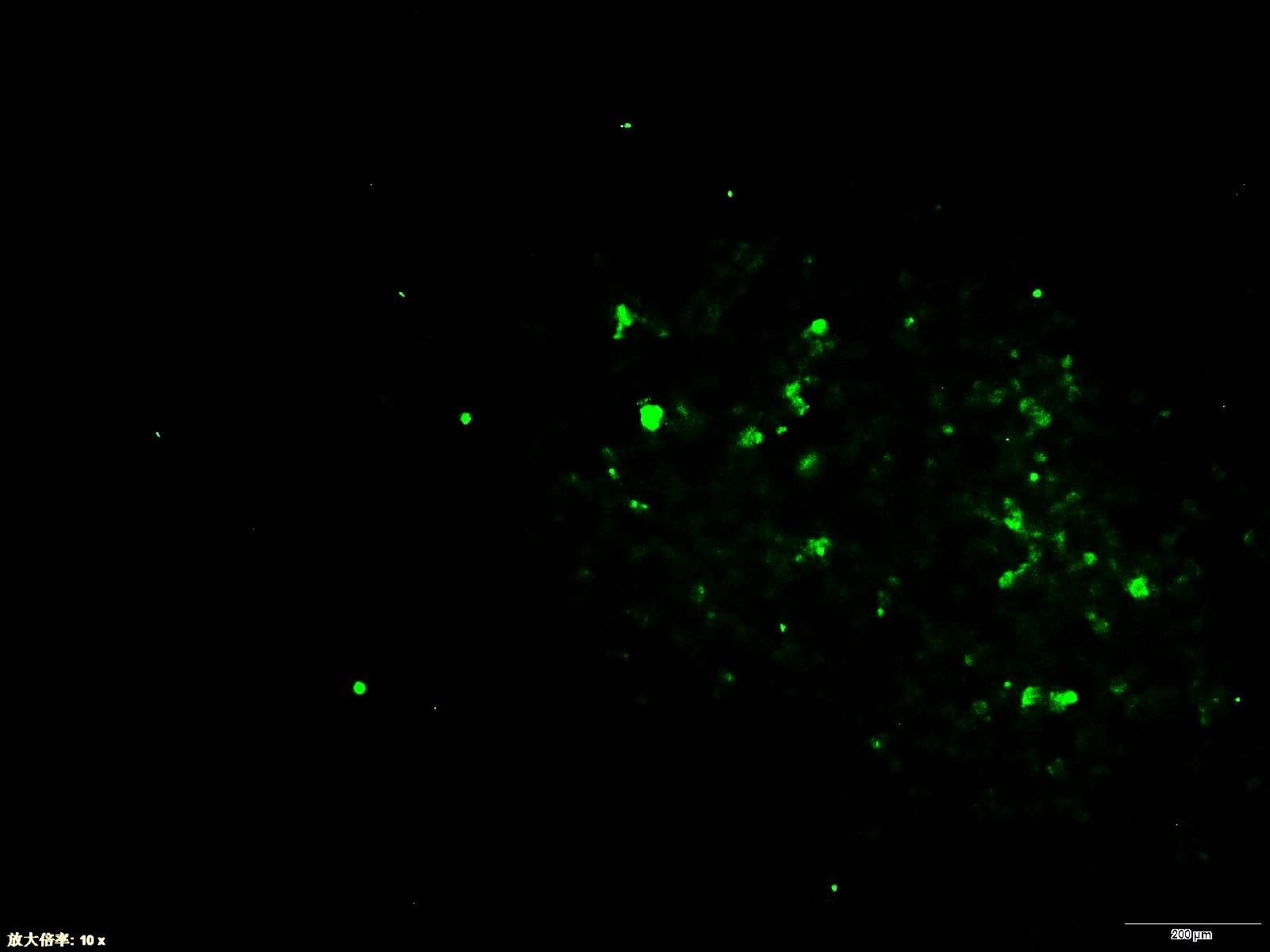

Supplement: Supplementary file 4 [file Data_Sheet_2.ZIP › cellular uptake-RAW.264.7/S1-Trimer/F.jpg]

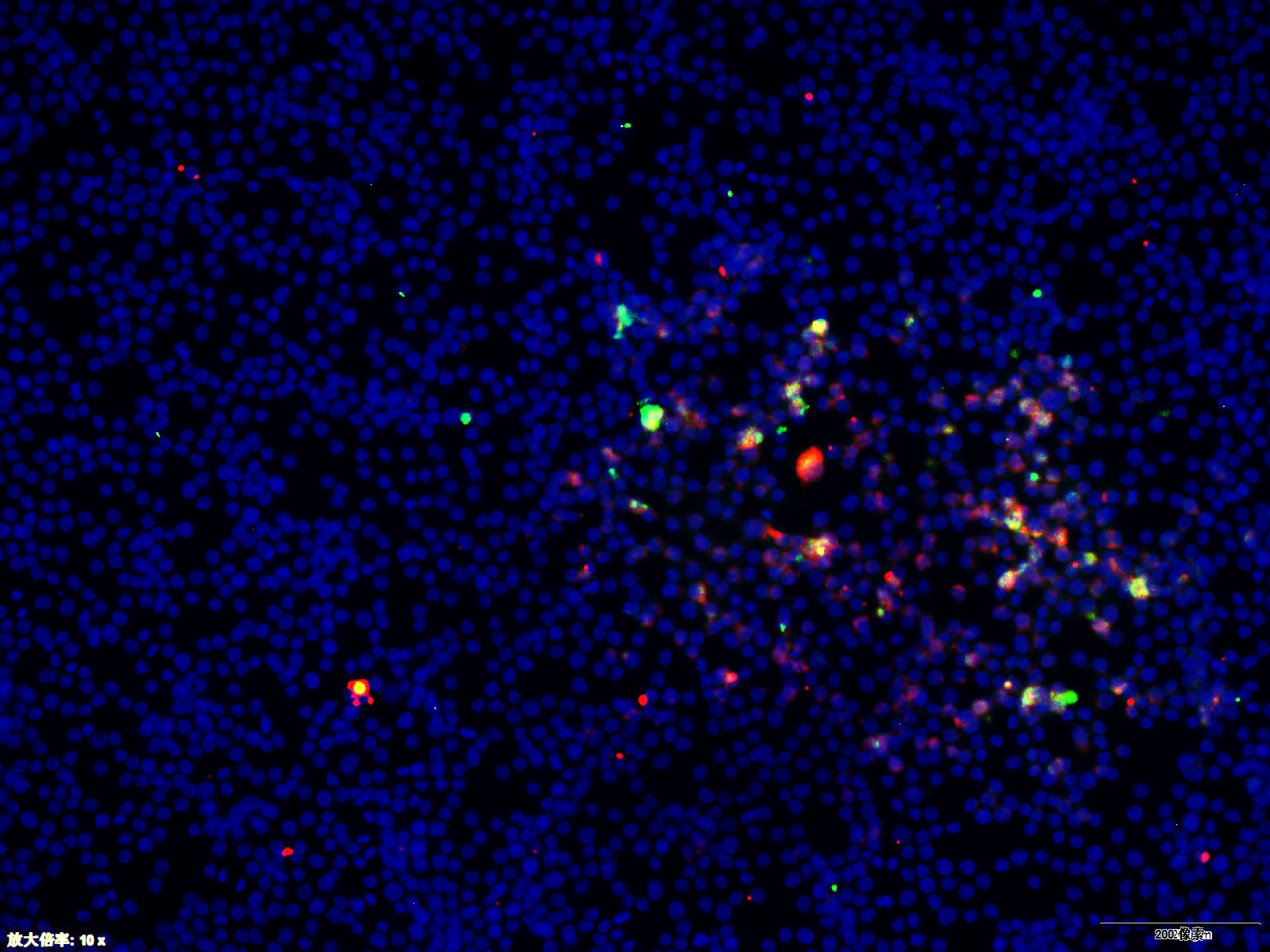

Supplement: Supplementary file 4 [file Data_Sheet_2.ZIP › cellular uptake-RAW.264.7/S1-Trimer/M.jpg]

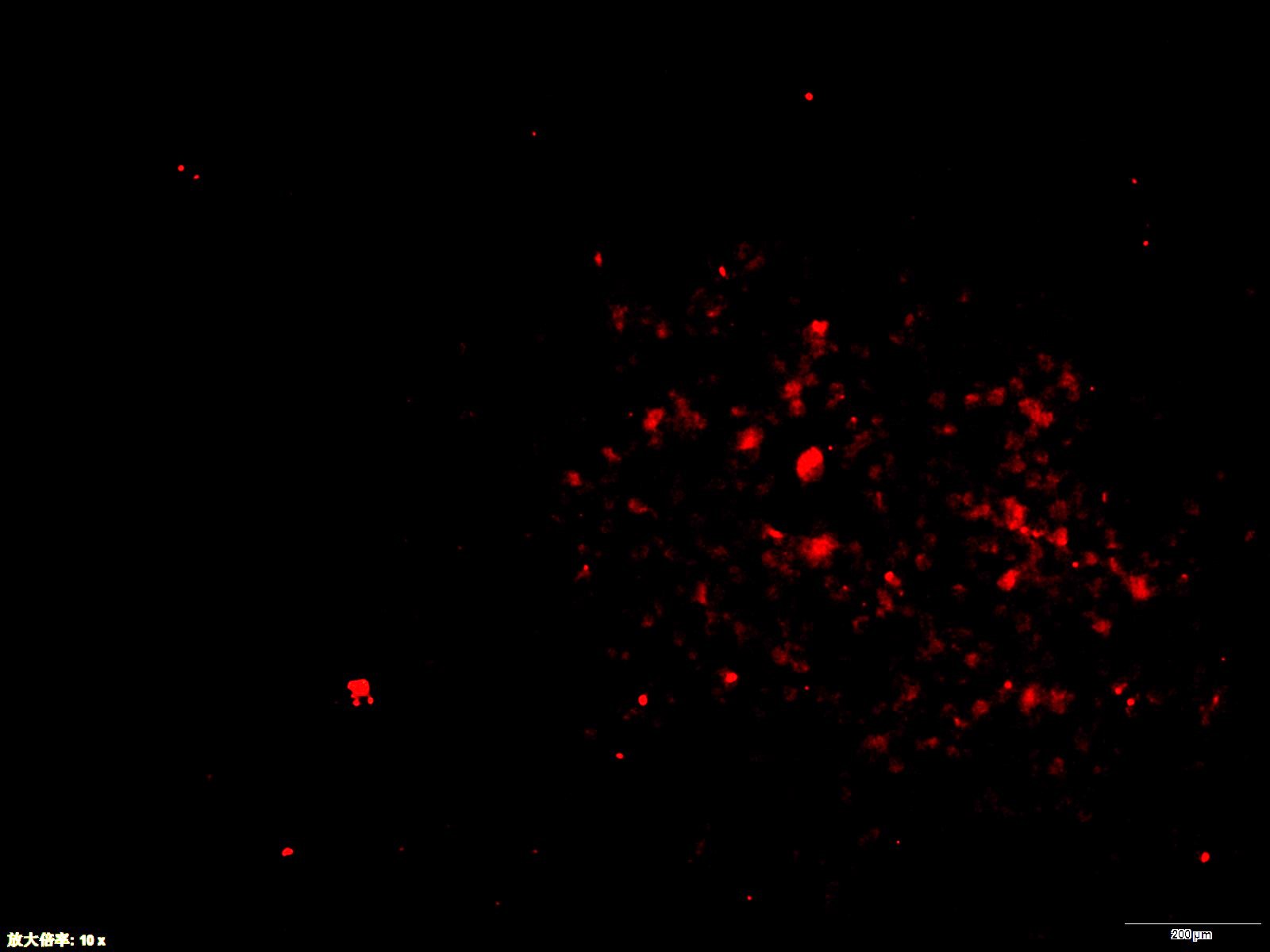

Supplement: Supplementary file 4 [file Data_Sheet_2.ZIP › cellular uptake-RAW.264.7/S1-Trimer/T.jpg]

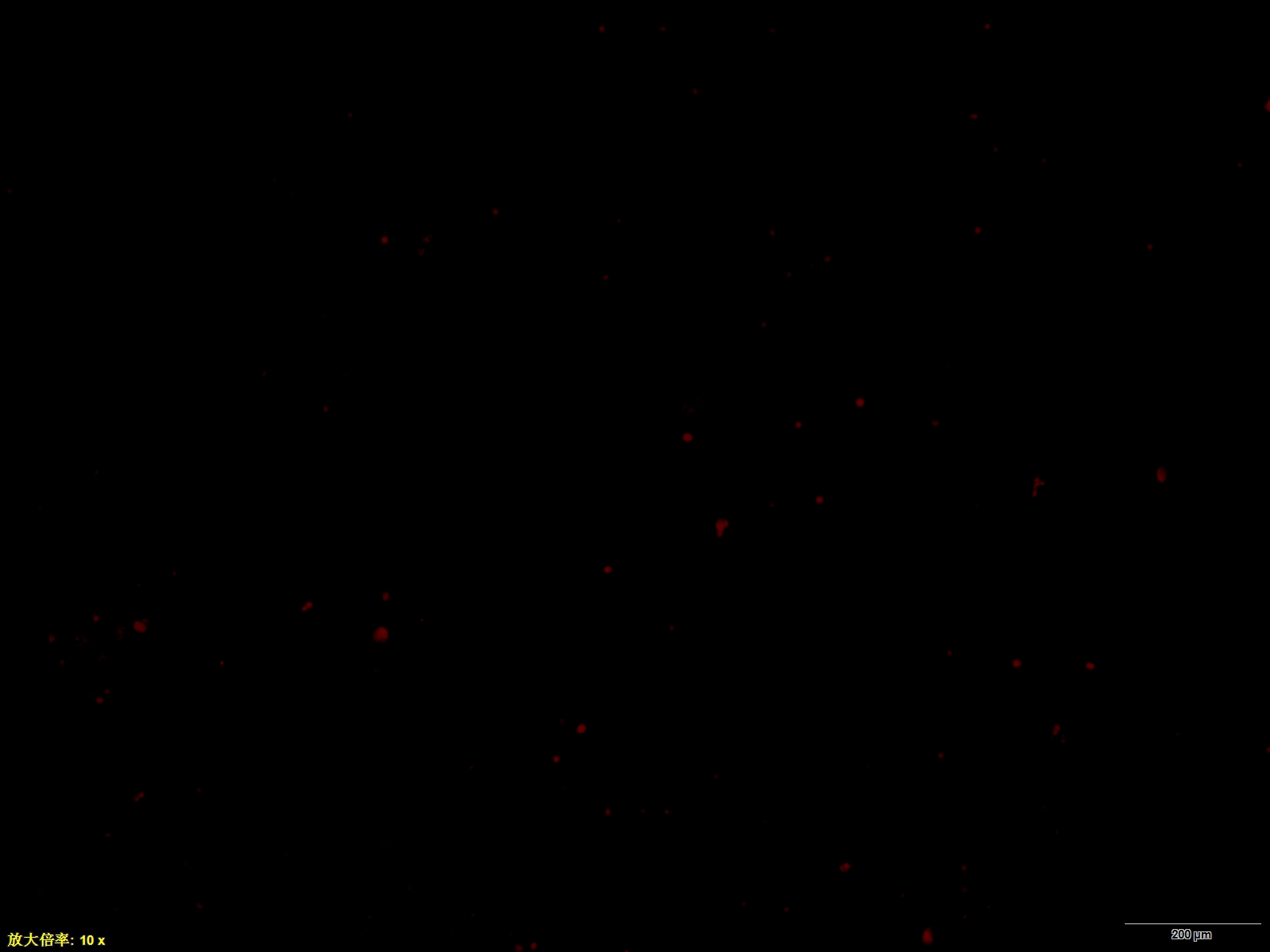

Supplement: Supplementary file 5 [file Data_Sheet_3.ZIP › cellular uptake-Vero/COE-Monomer/D.jpg]

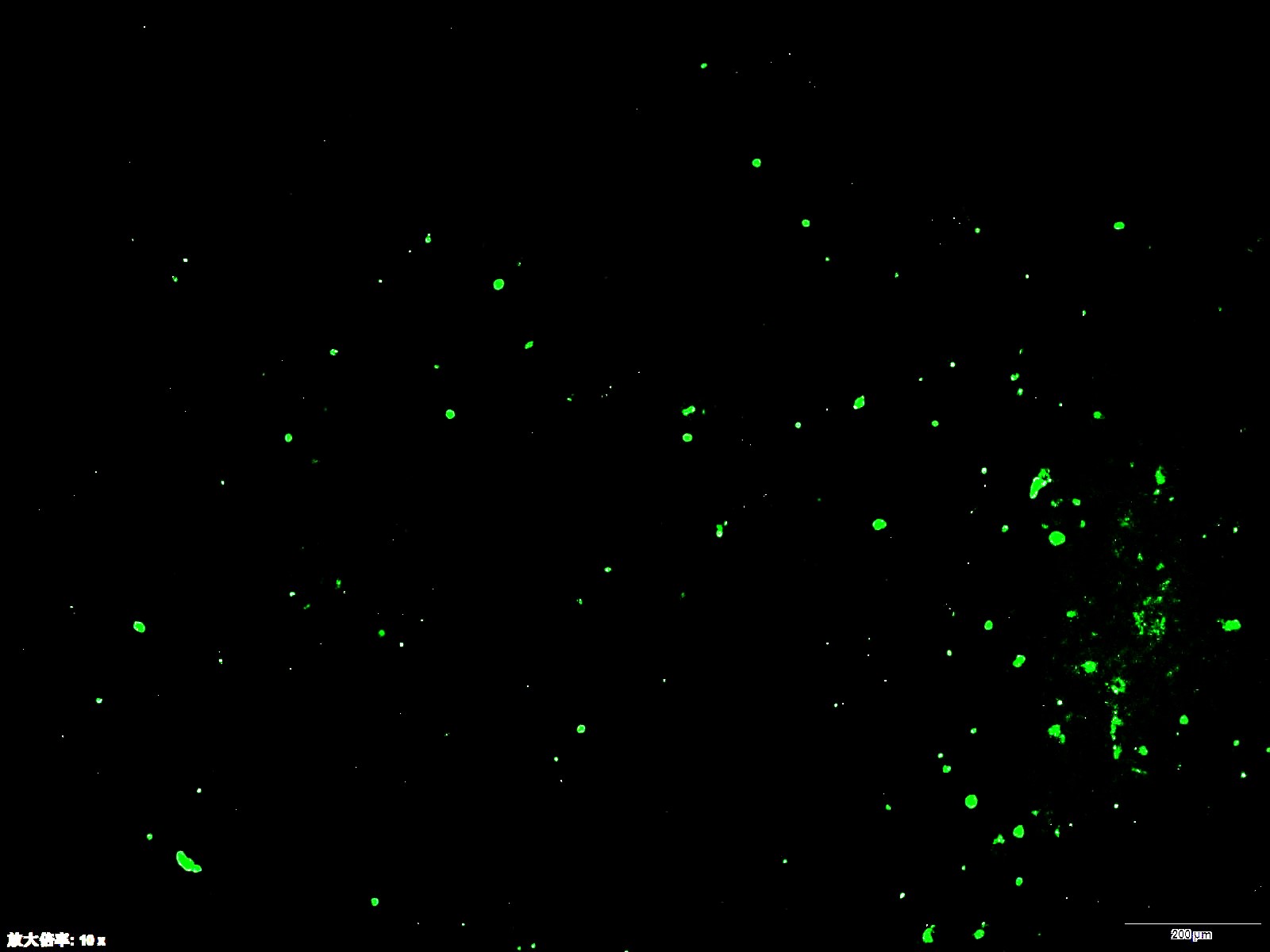

Supplement: Supplementary file 5 [file Data_Sheet_3.ZIP › cellular uptake-Vero/COE-Monomer/F.jpg]

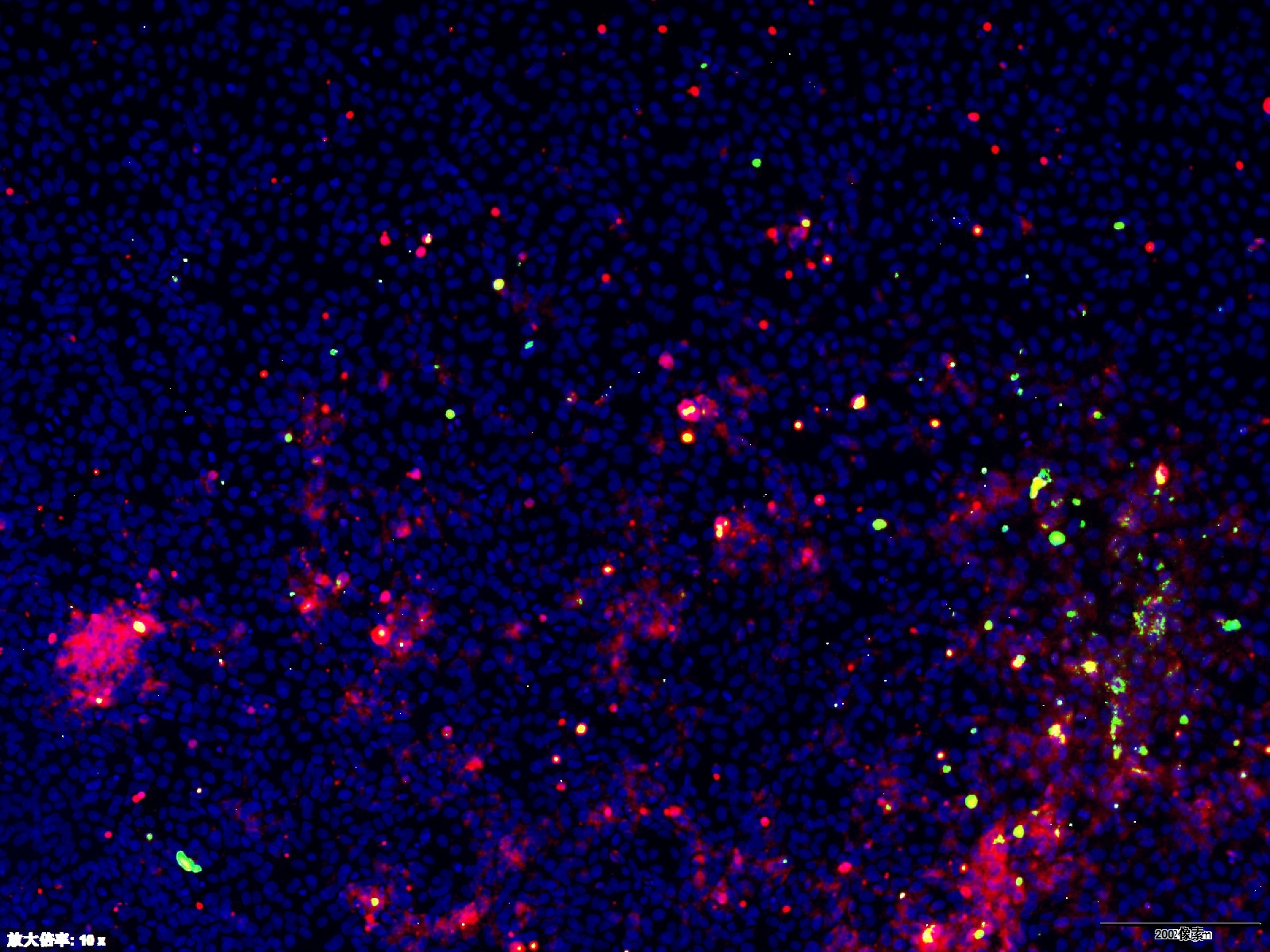

Supplement: Supplementary file 5 [file Data_Sheet_3.ZIP › cellular uptake-Vero/COE-Monomer/M.jpg]

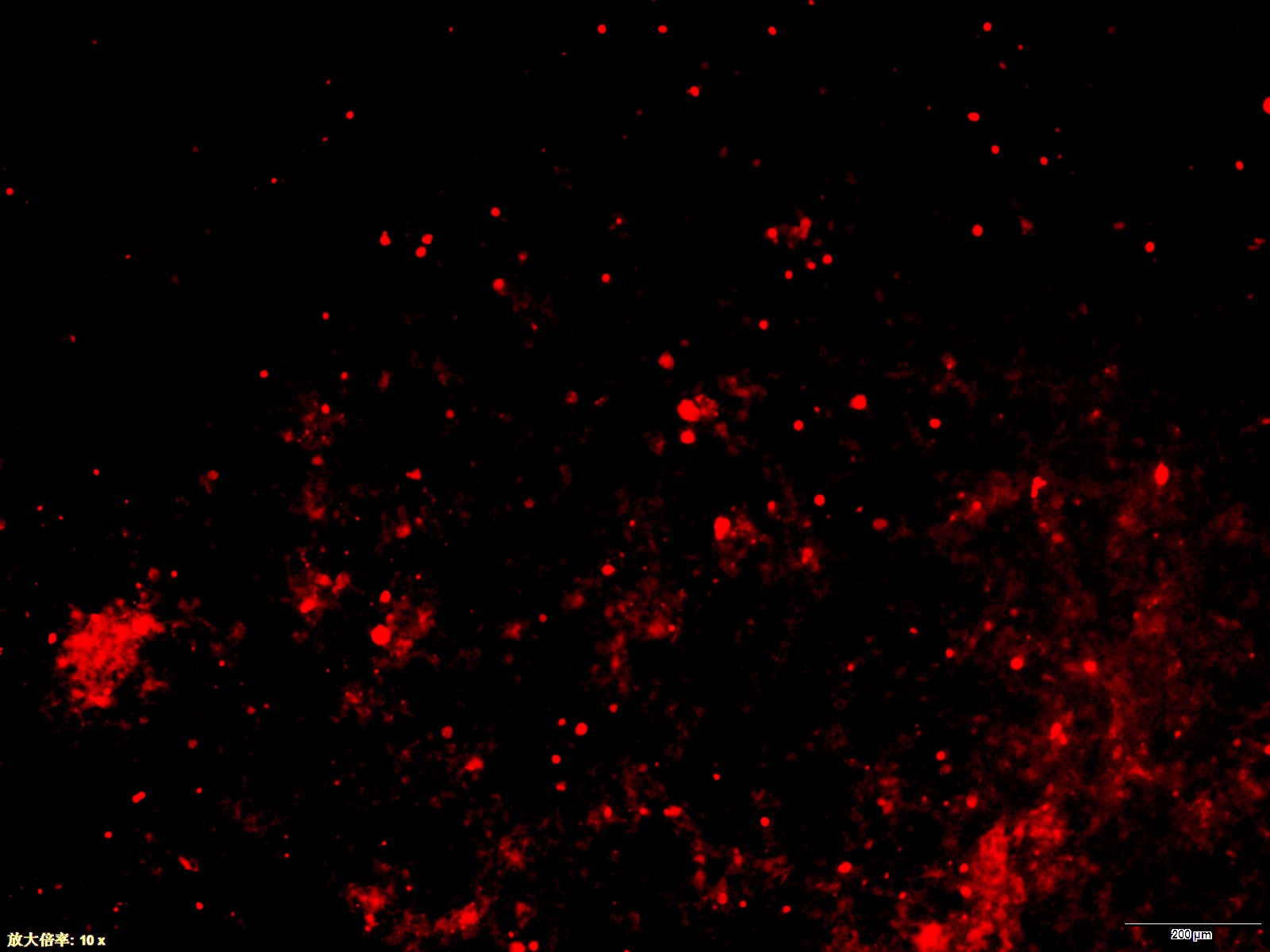

Supplement: Supplementary file 5 [file Data_Sheet_3.ZIP › cellular uptake-Vero/COE-Monomer/T.jpg]

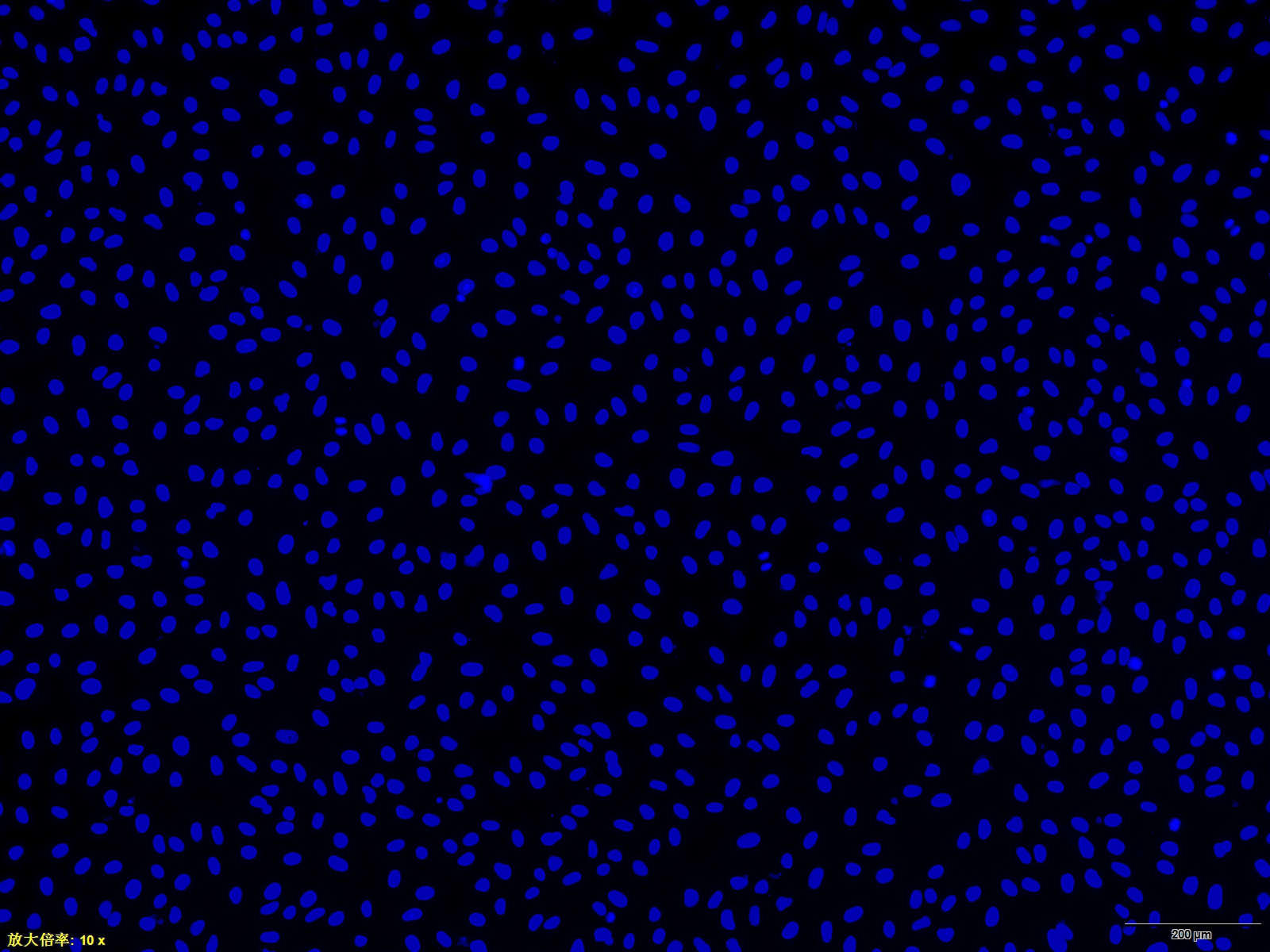

Supplement: Supplementary file 5 [file Data_Sheet_3.ZIP › cellular uptake-Vero/COE-Trimer/D.jpg]

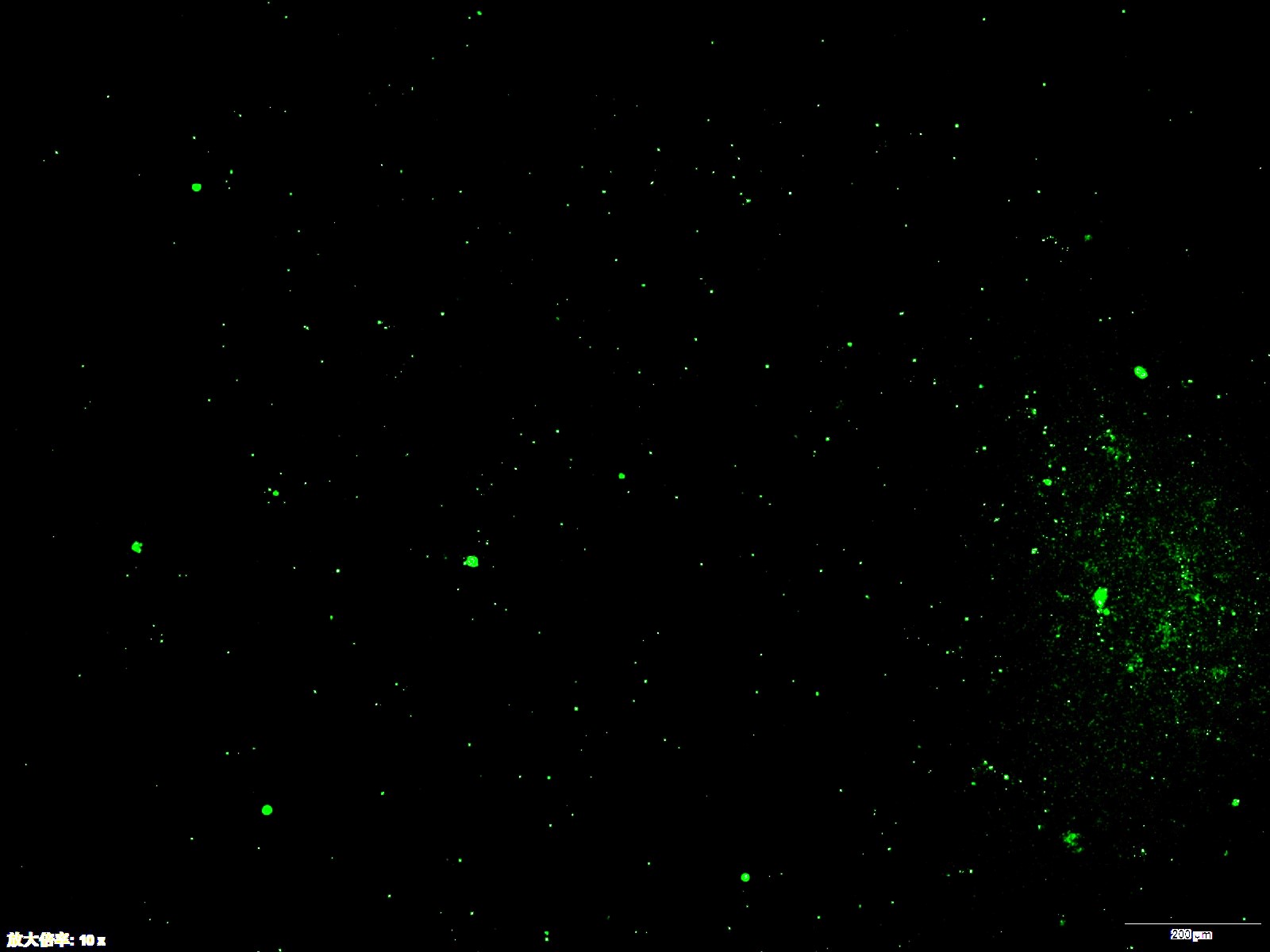

Supplement: Supplementary file 5 [file Data_Sheet_3.ZIP › cellular uptake-Vero/COE-Trimer/F.jpg]

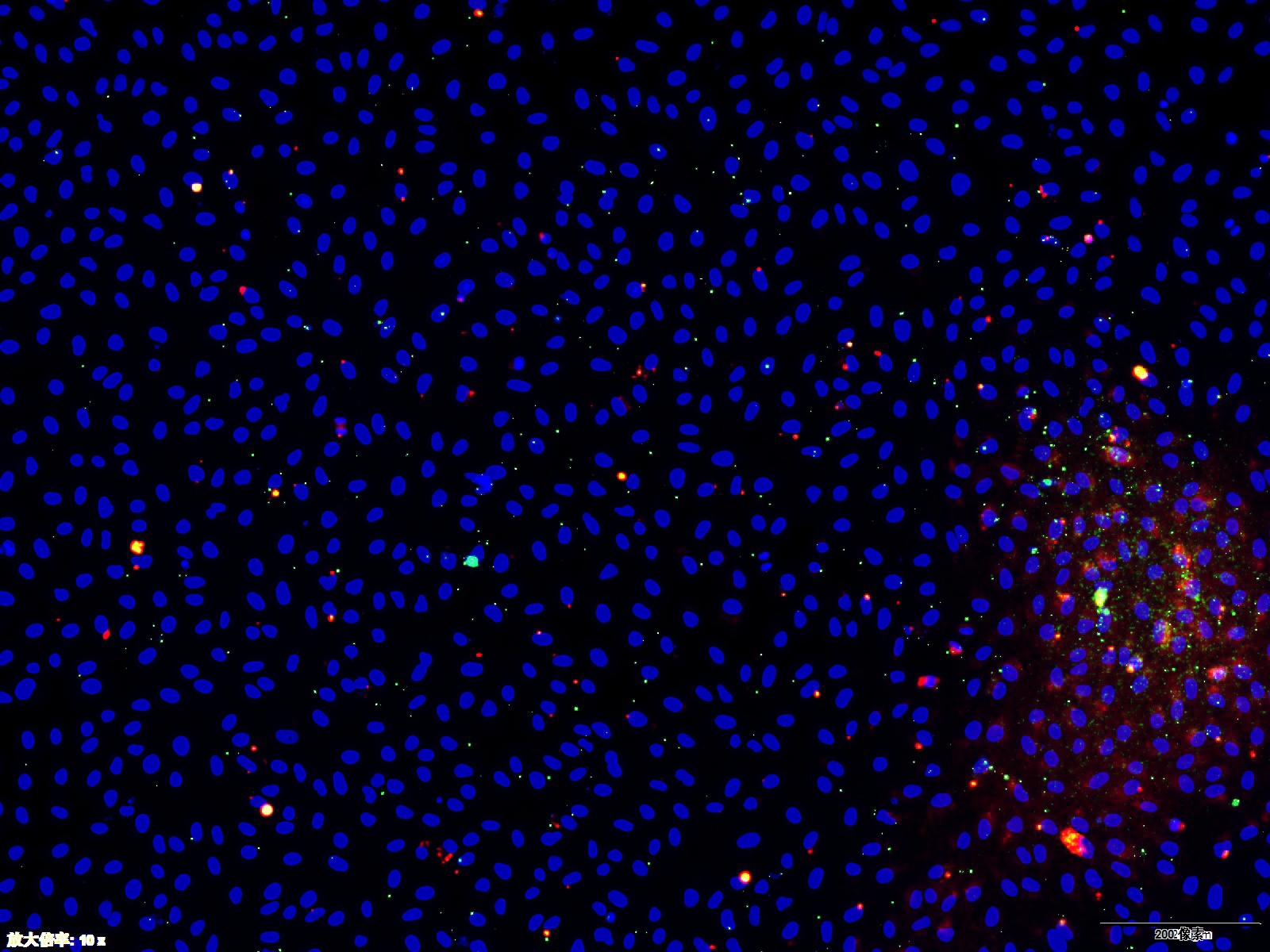

Supplement: Supplementary file 5 [file Data_Sheet_3.ZIP › cellular uptake-Vero/COE-Trimer/M.jpg]

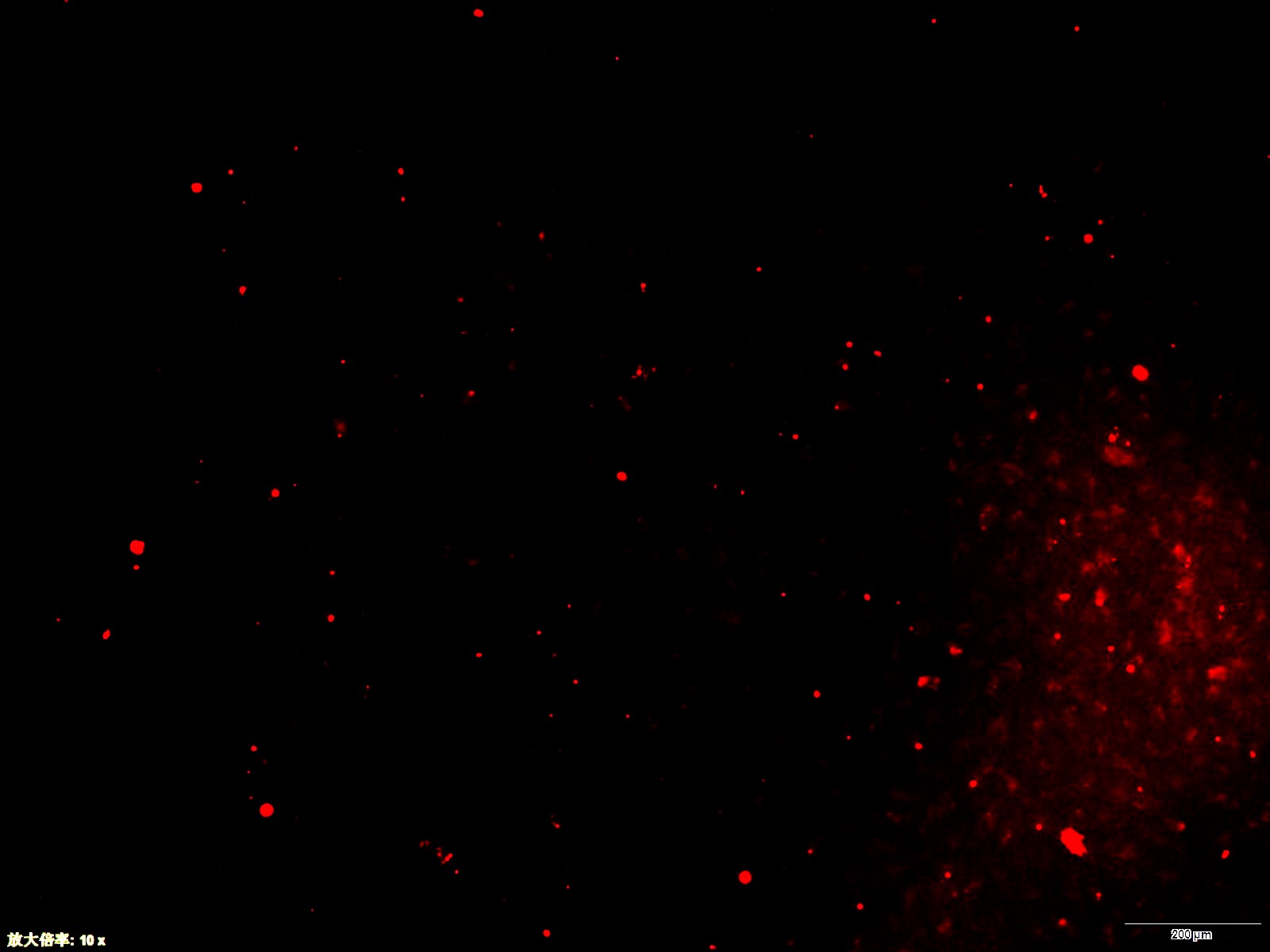

Supplement: Supplementary file 5 [file Data_Sheet_3.ZIP › cellular uptake-Vero/COE-Trimer/T.jpg]

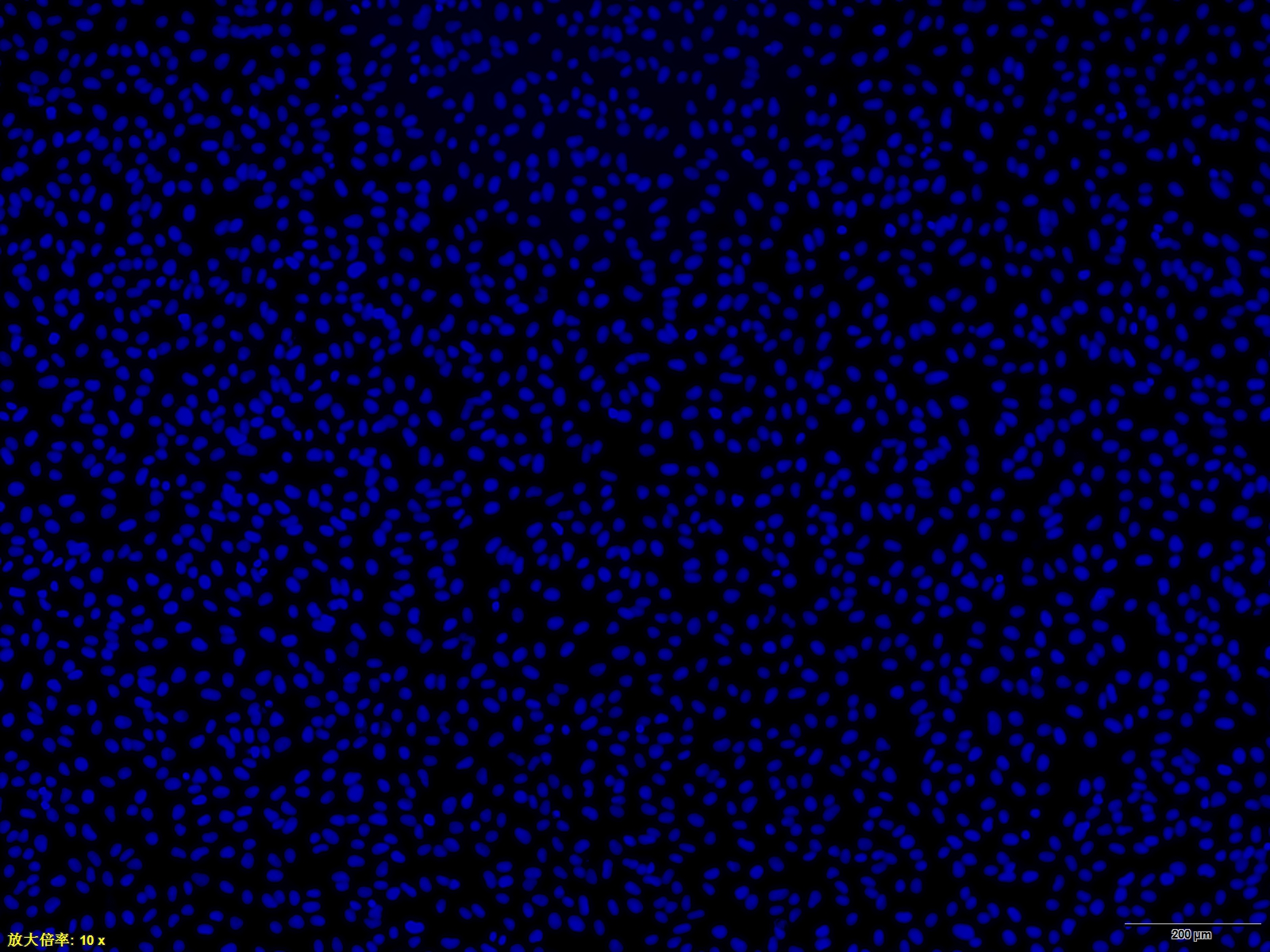

Supplement: Supplementary file 5 [file Data_Sheet_3.ZIP › cellular uptake-Vero/RBD-Monomer/D.jpg]

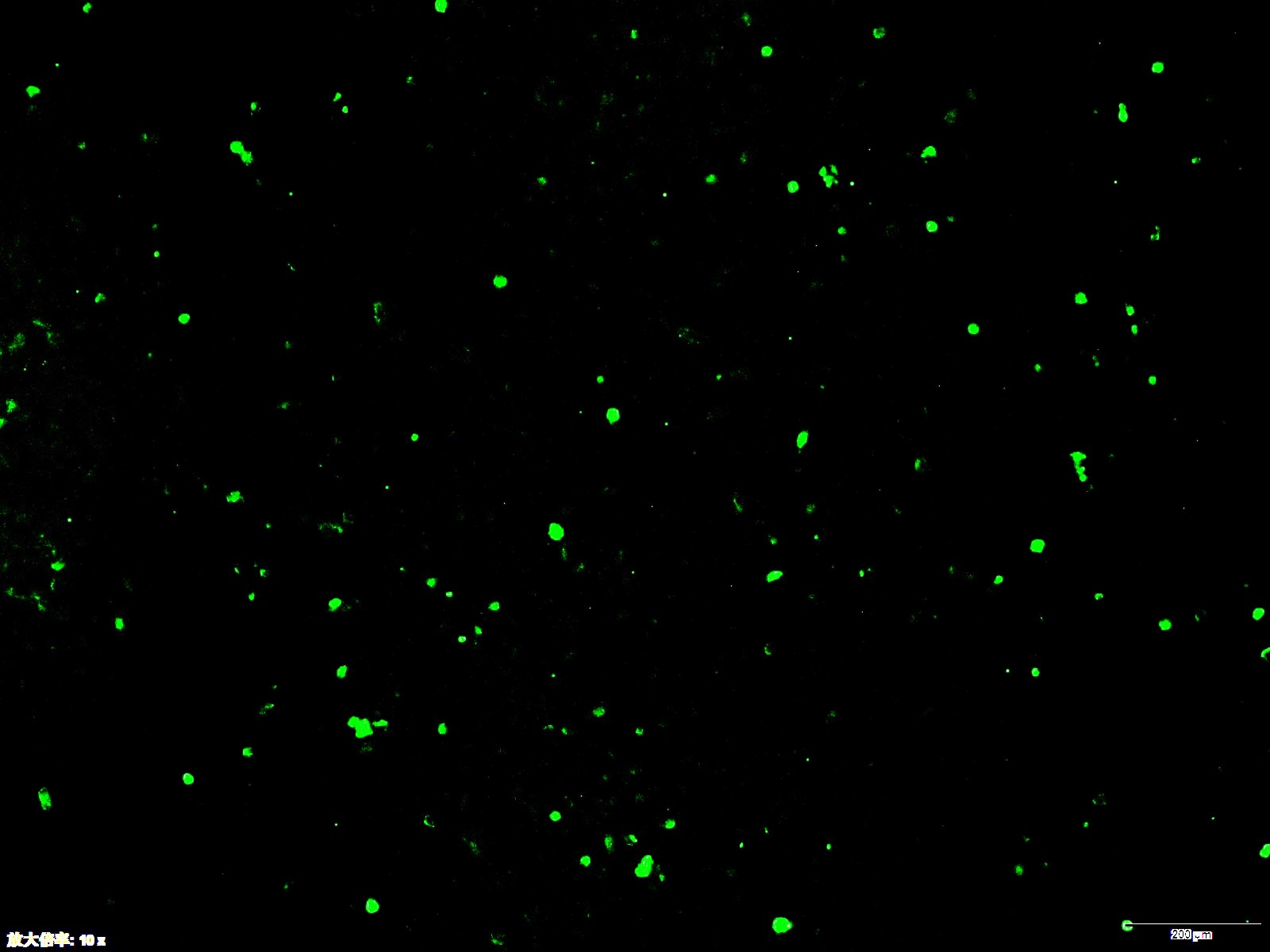

Supplement: Supplementary file 5 [file Data_Sheet_3.ZIP › cellular uptake-Vero/RBD-Monomer/F.jpg]

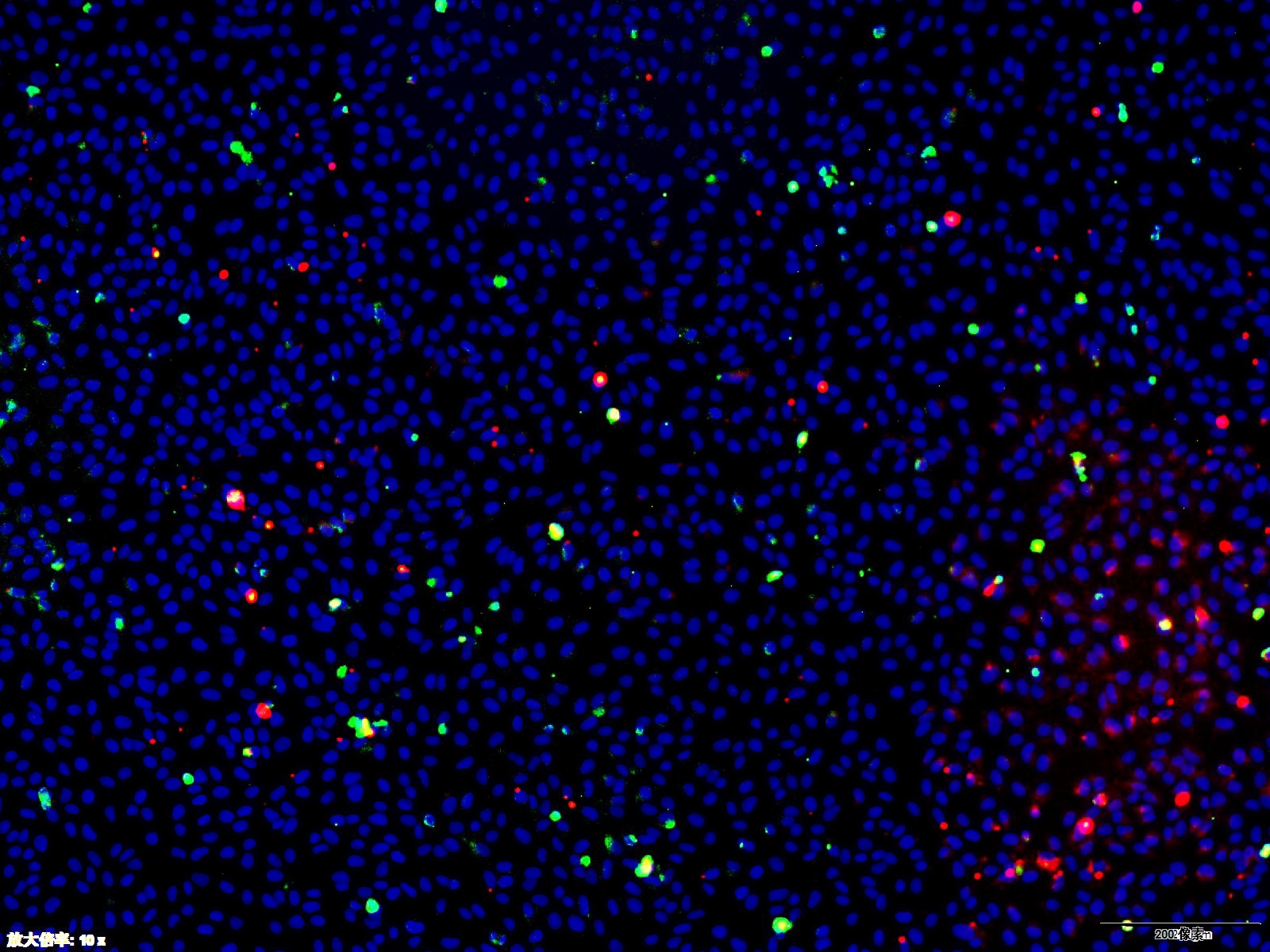

Supplement: Supplementary file 5 [file Data_Sheet_3.ZIP › cellular uptake-Vero/RBD-Monomer/M.jpg]

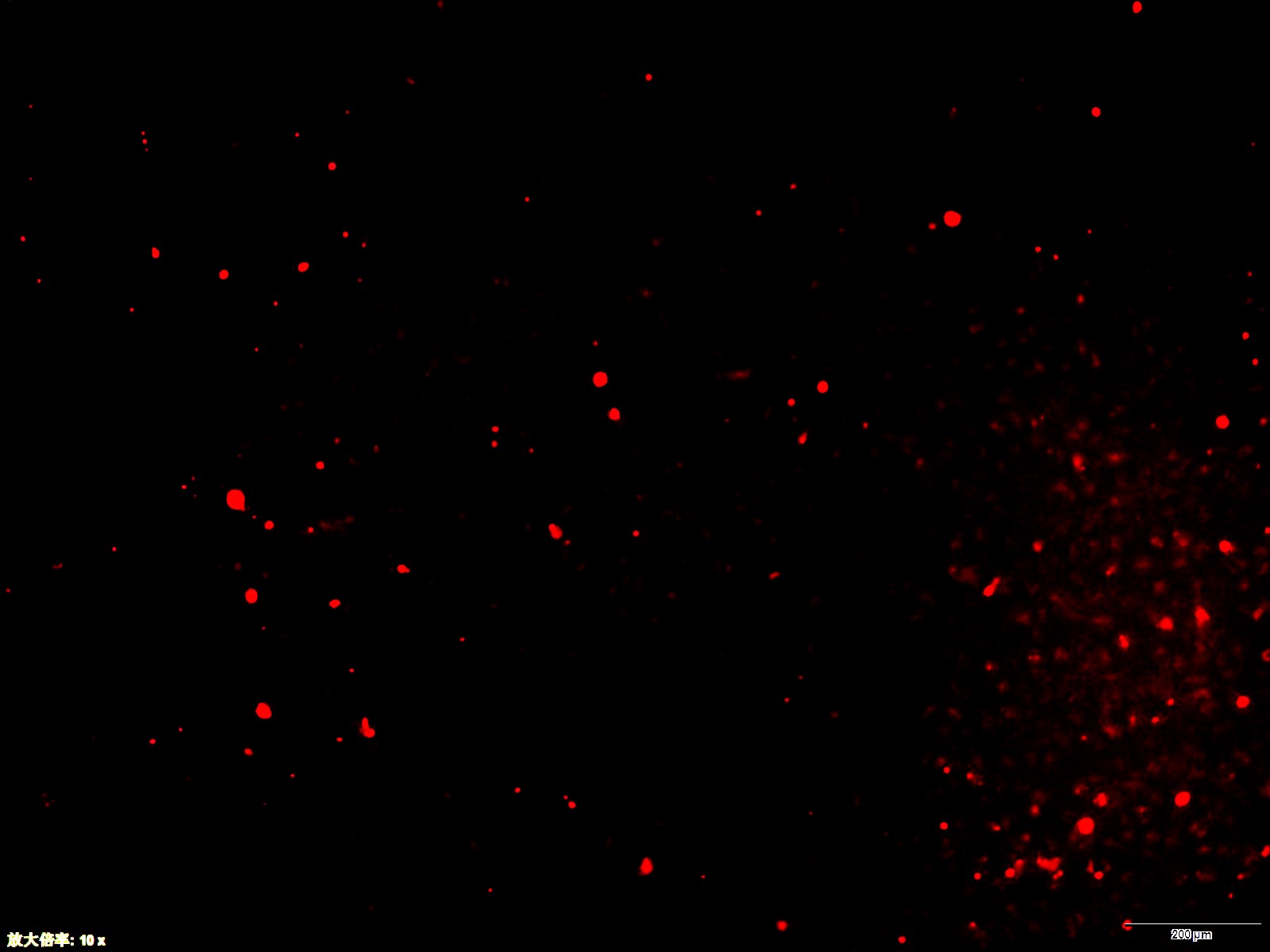

Supplement: Supplementary file 5 [file Data_Sheet_3.ZIP › cellular uptake-Vero/RBD-Monomer/T.jpg]

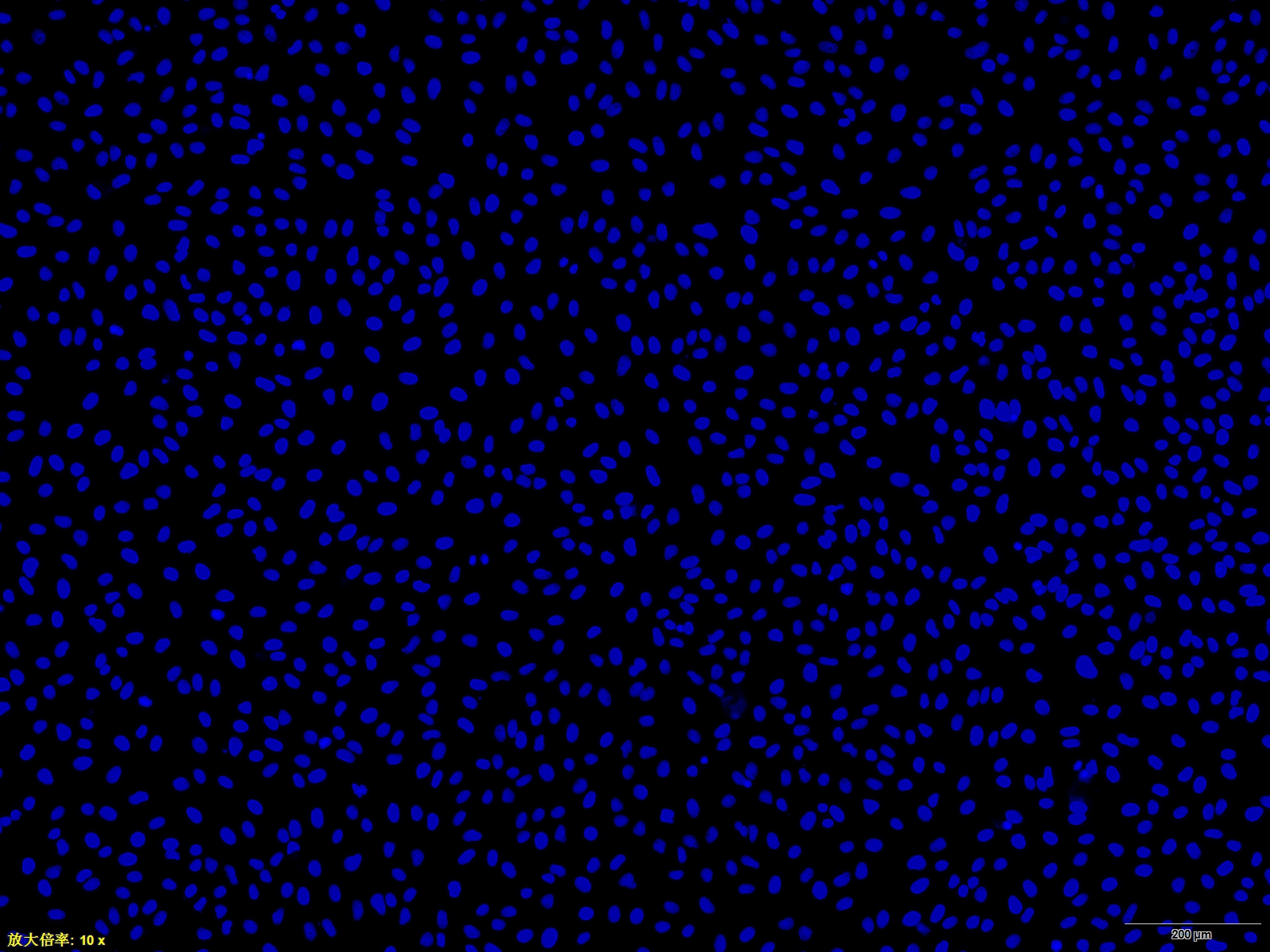

Supplement: Supplementary file 5 [file Data_Sheet_3.ZIP › cellular uptake-Vero/RBD-Trimer/D.jpg]

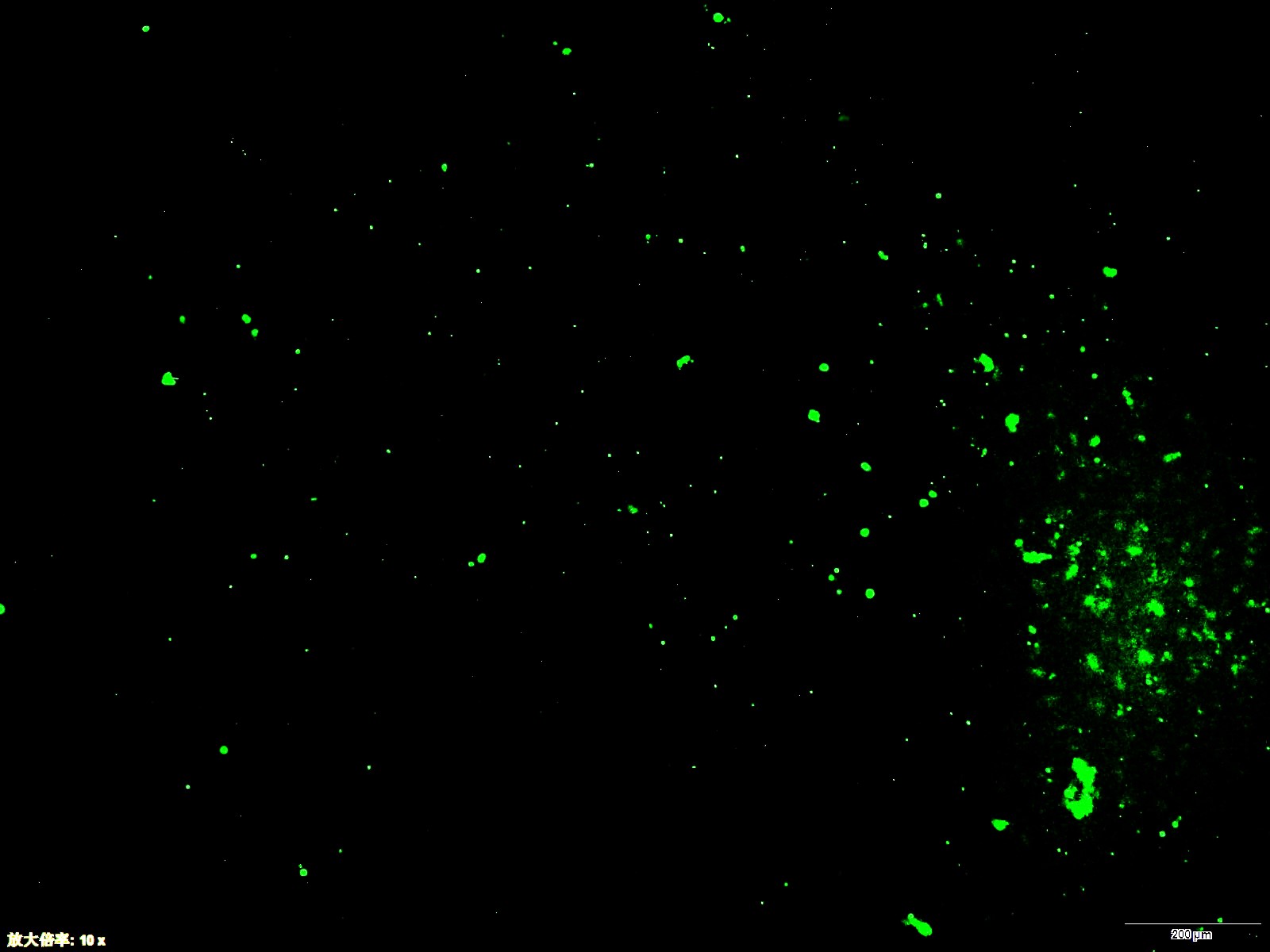

Supplement: Supplementary file 5 [file Data_Sheet_3.ZIP › cellular uptake-Vero/RBD-Trimer/F.jpg]

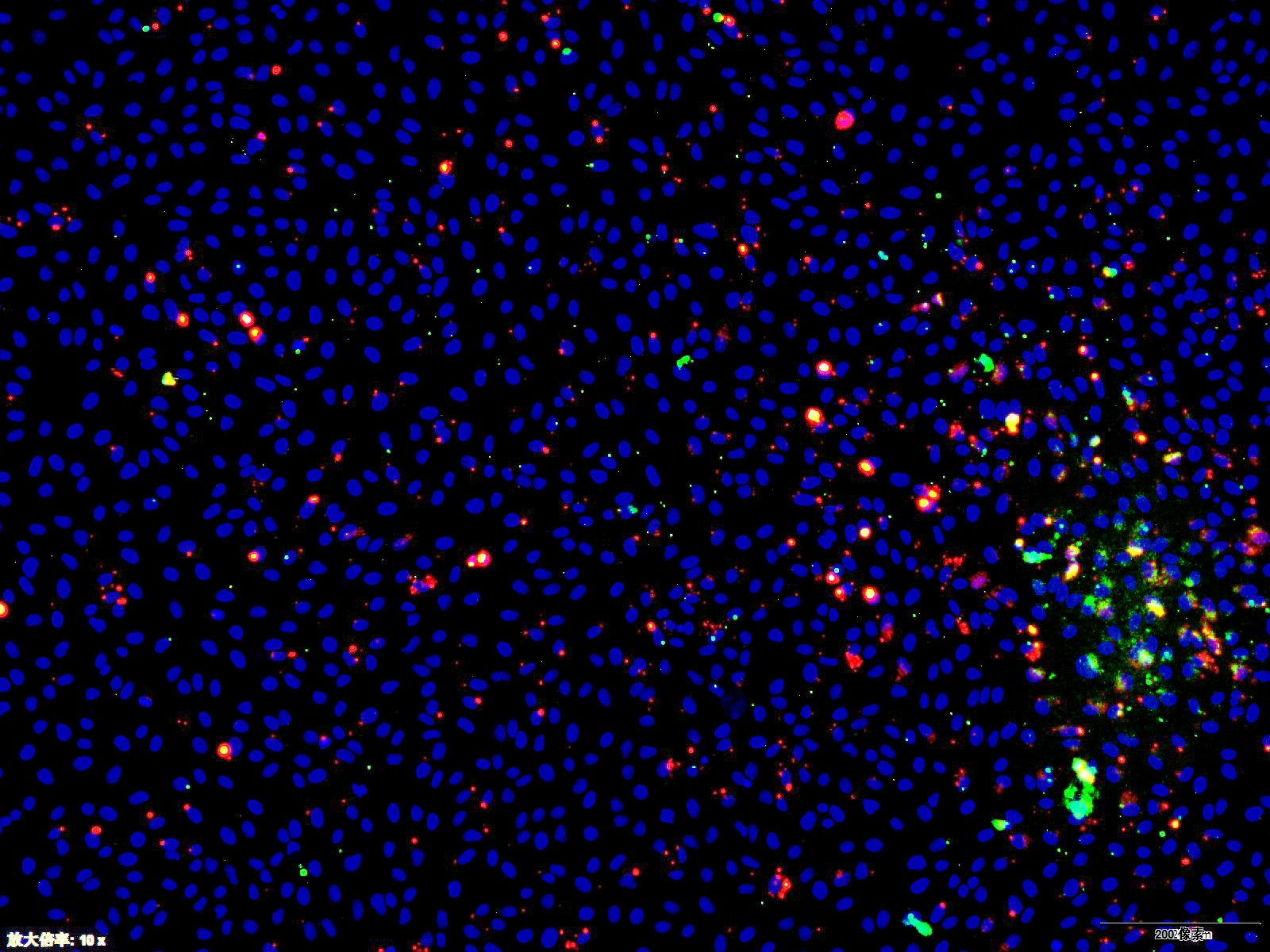

Supplement: Supplementary file 5 [file Data_Sheet_3.ZIP › cellular uptake-Vero/RBD-Trimer/M.jpg]

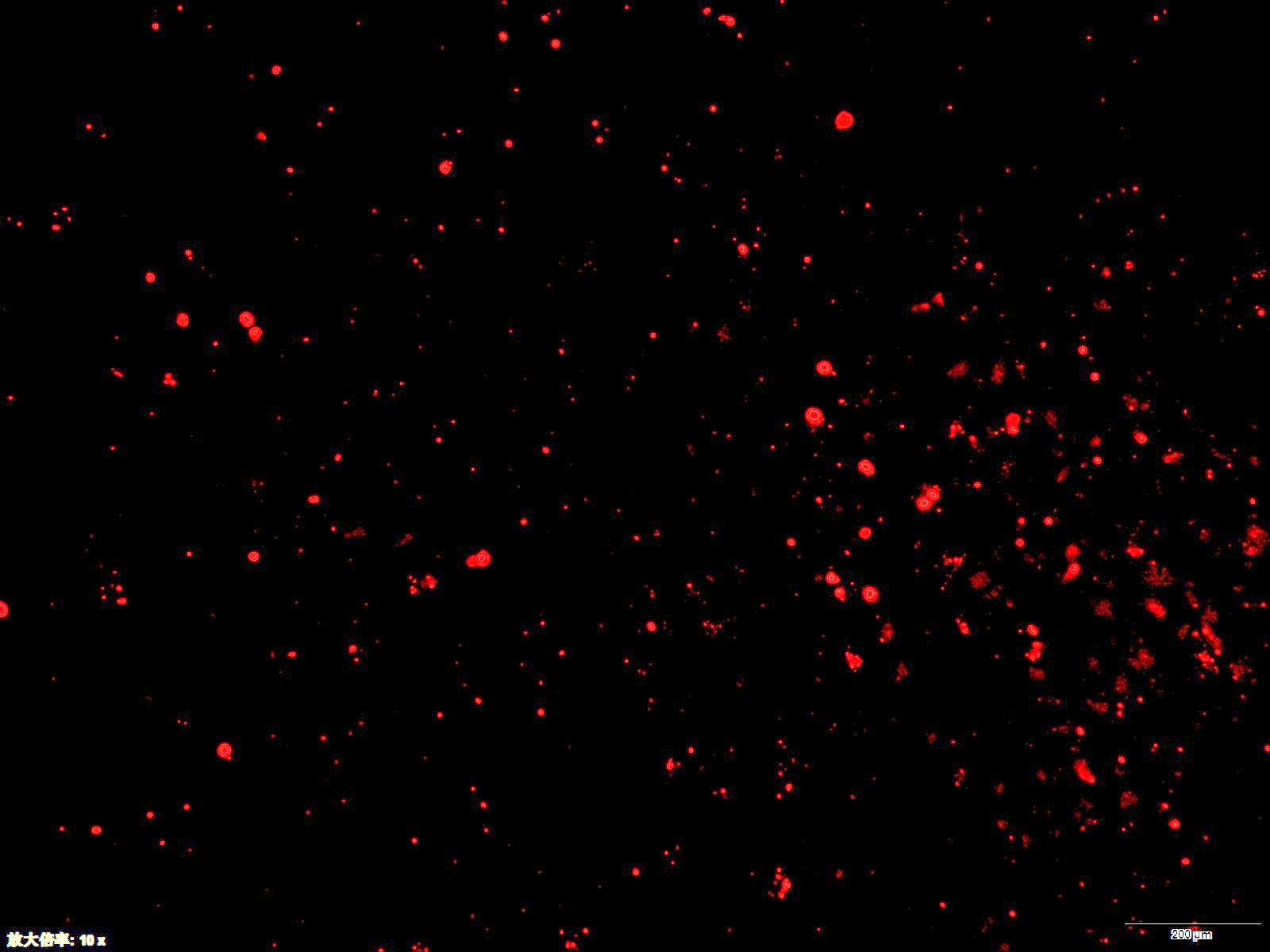

Supplement: Supplementary file 5 [file Data_Sheet_3.ZIP › cellular uptake-Vero/RBD-Trimer/T.jpg]

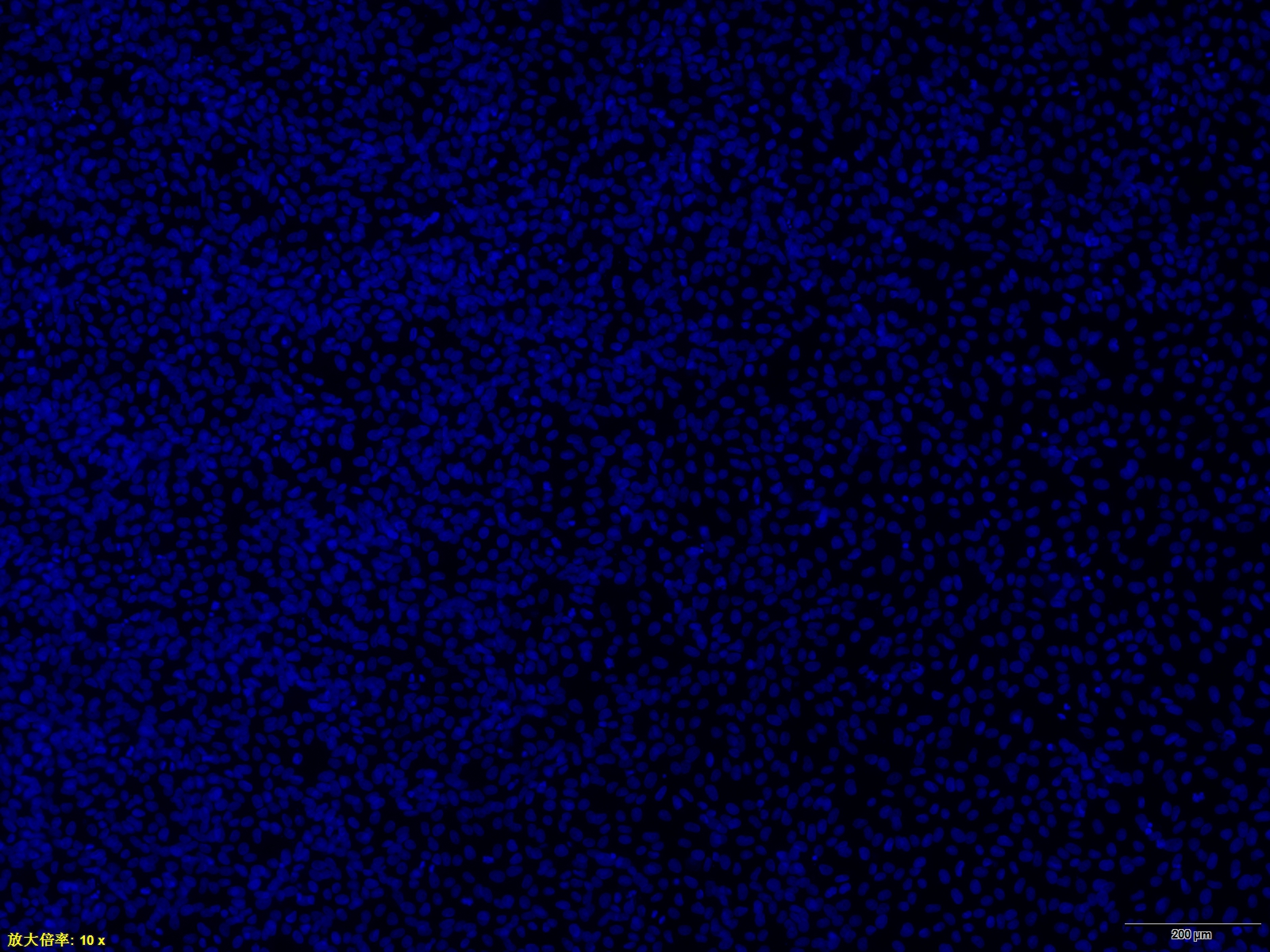

Supplement: Supplementary file 5 [file Data_Sheet_3.ZIP › cellular uptake-Vero/S1-Monomer/D.jpg]

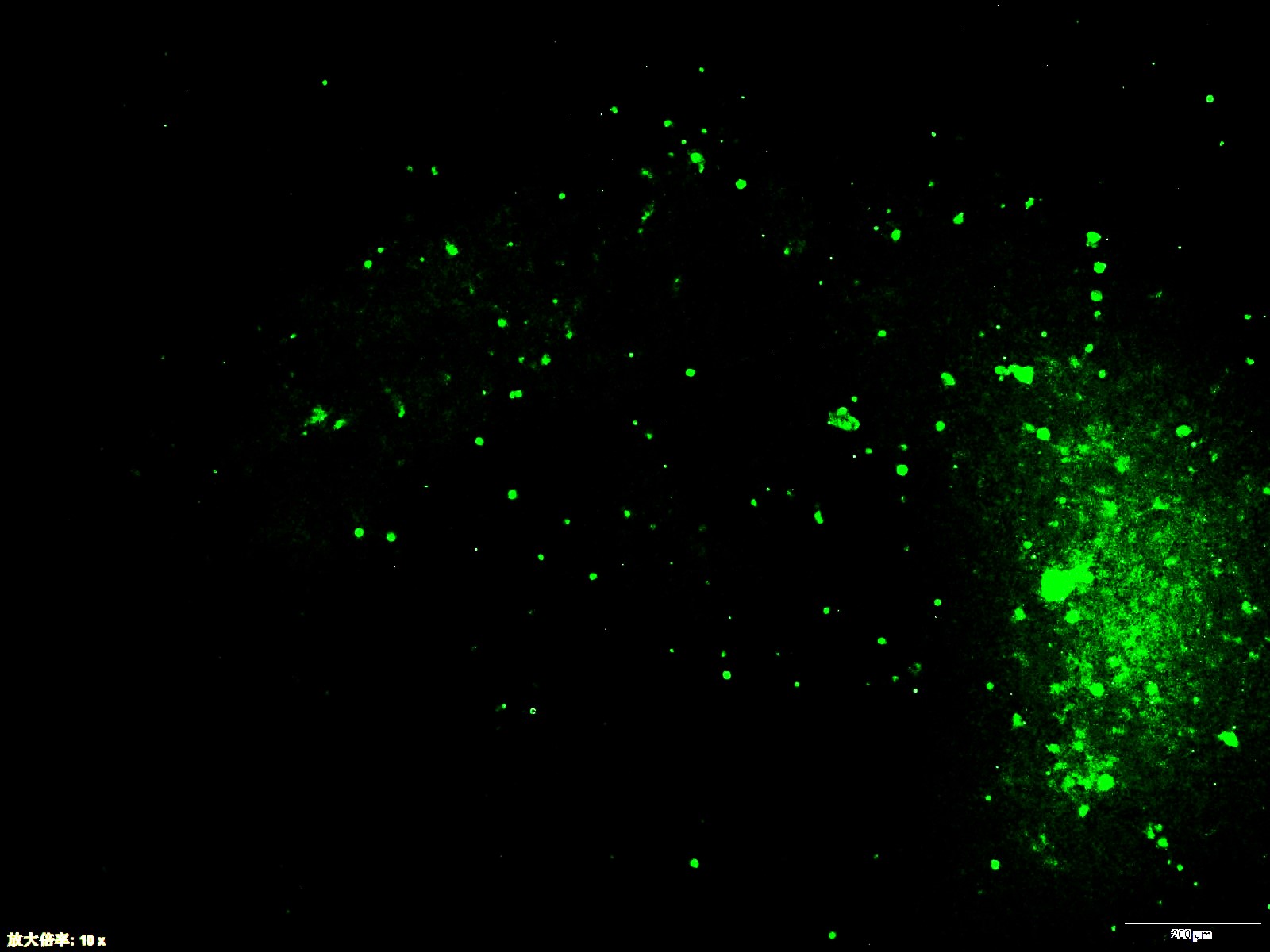

Supplement: Supplementary file 5 [file Data_Sheet_3.ZIP › cellular uptake-Vero/S1-Monomer/F.jpg]

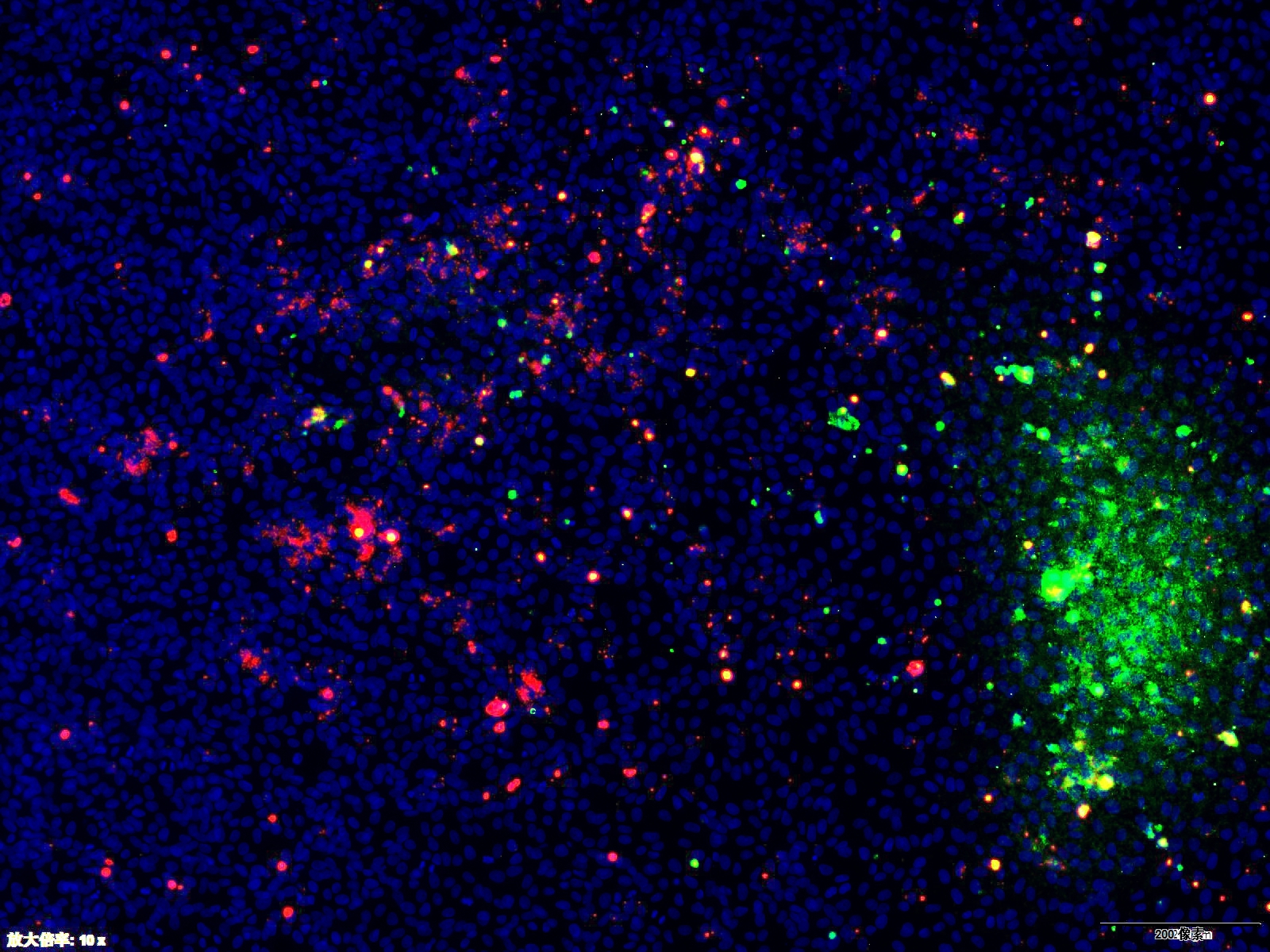

Supplement: Supplementary file 5 [file Data_Sheet_3.ZIP › cellular uptake-Vero/S1-Monomer/M.jpg]

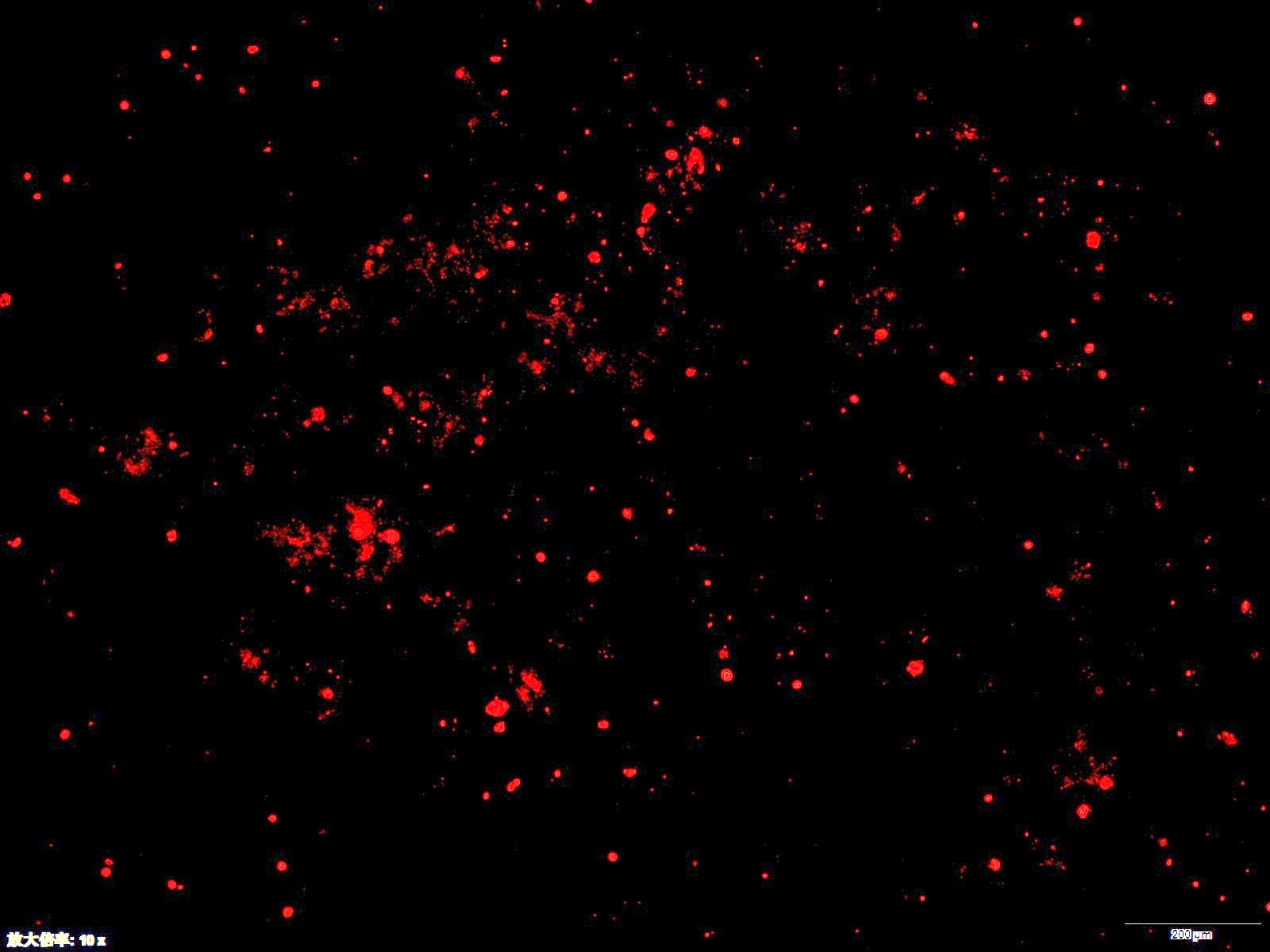

Supplement: Supplementary file 5 [file Data_Sheet_3.ZIP › cellular uptake-Vero/S1-Monomer/T.jpg]

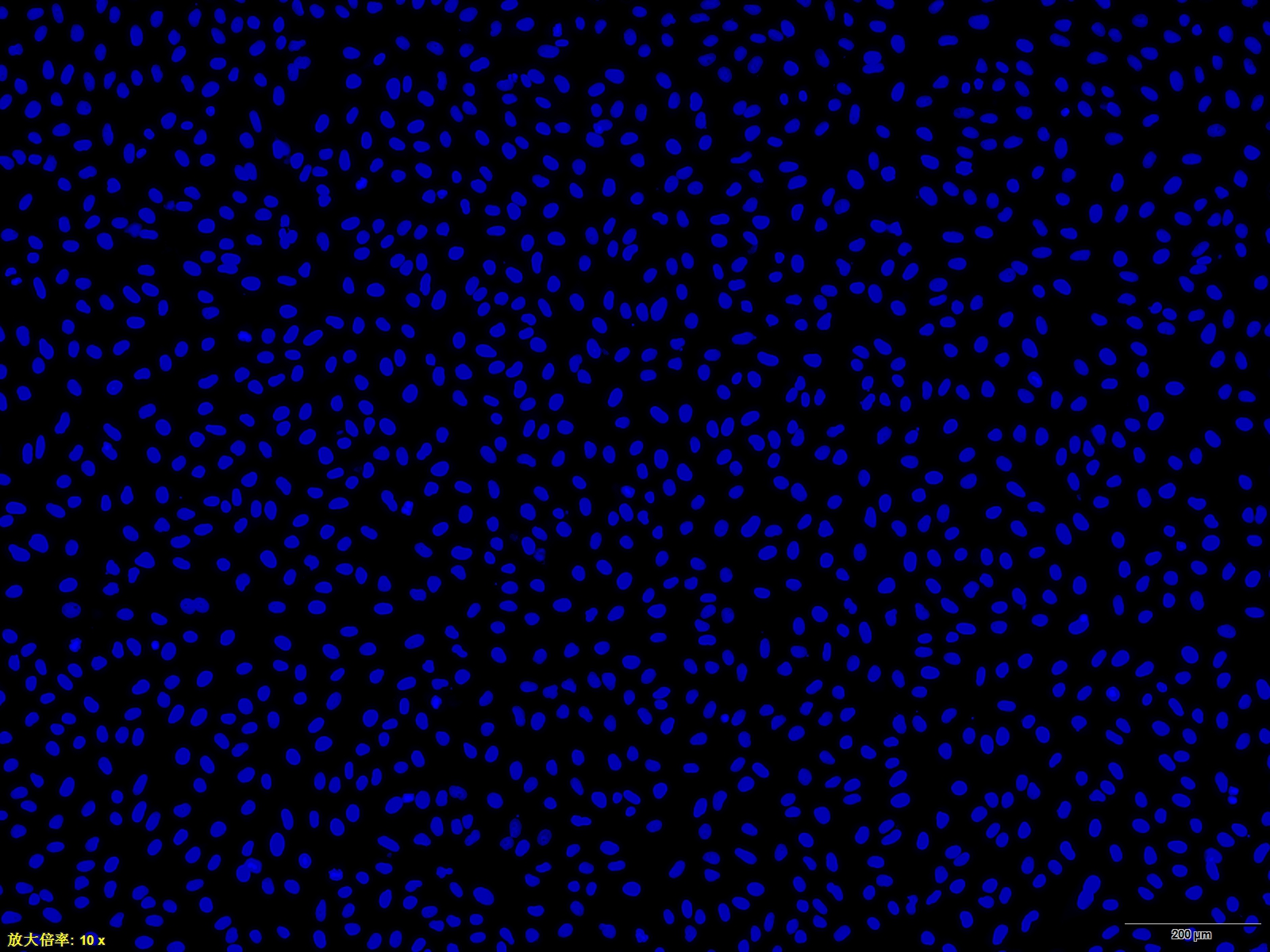

Supplement: Supplementary file 5 [file Data_Sheet_3.ZIP › cellular uptake-Vero/S1-Trimer/D.jpg]

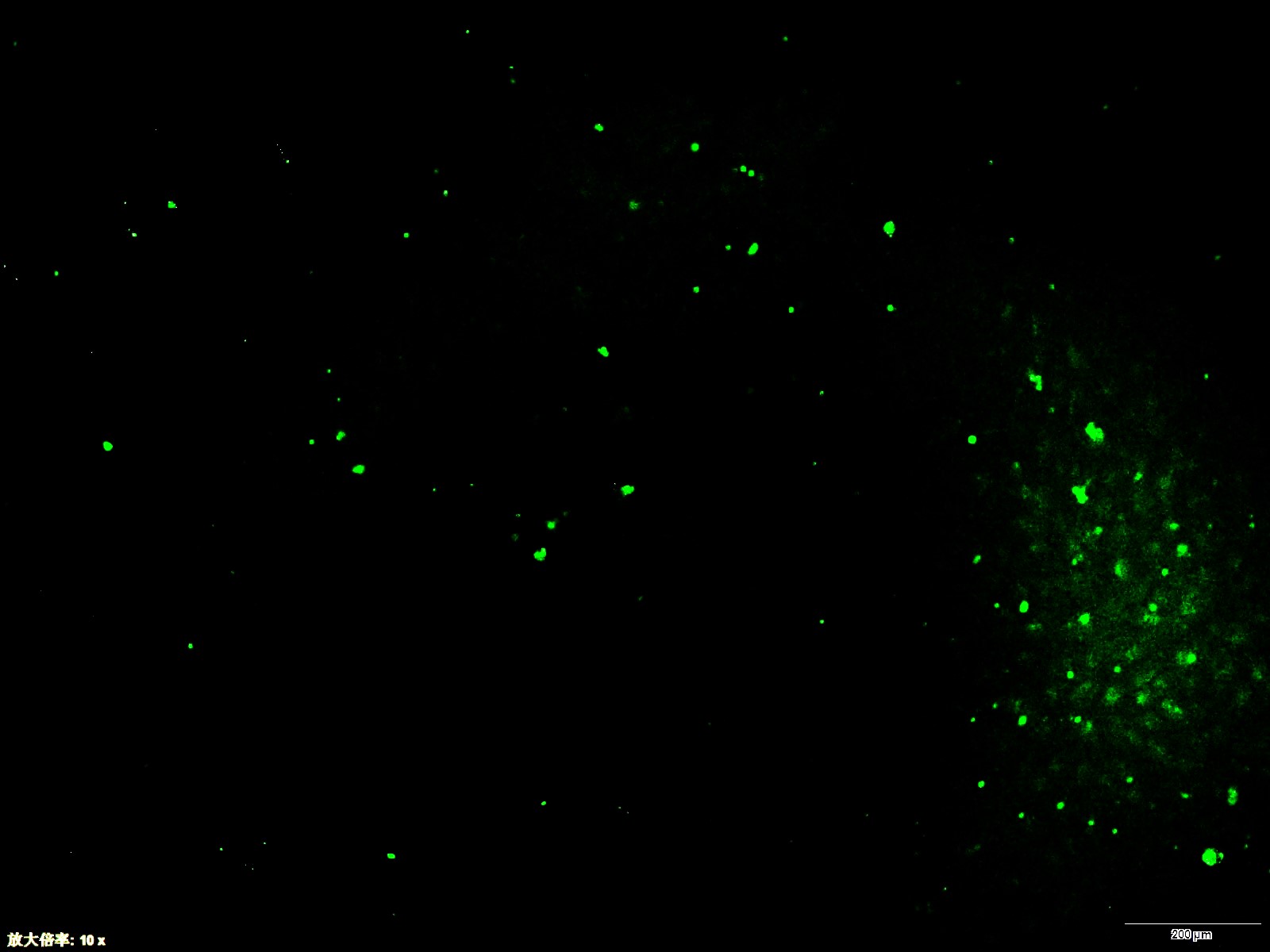

Supplement: Supplementary file 5 [file Data_Sheet_3.ZIP › cellular uptake-Vero/S1-Trimer/F.jpg]

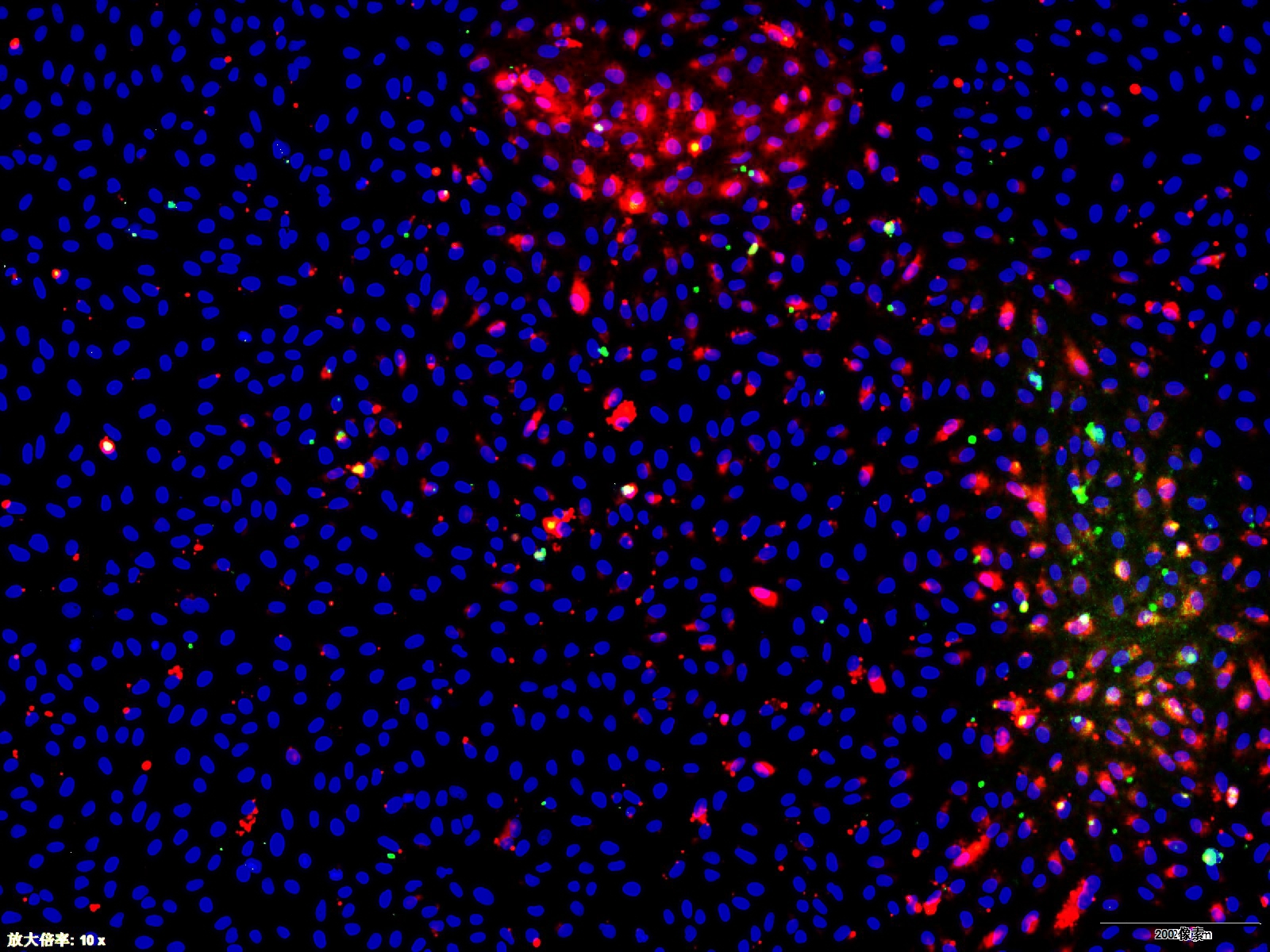

Supplement: Supplementary file 5 [file Data_Sheet_3.ZIP › cellular uptake-Vero/S1-Trimer/M.jpg]

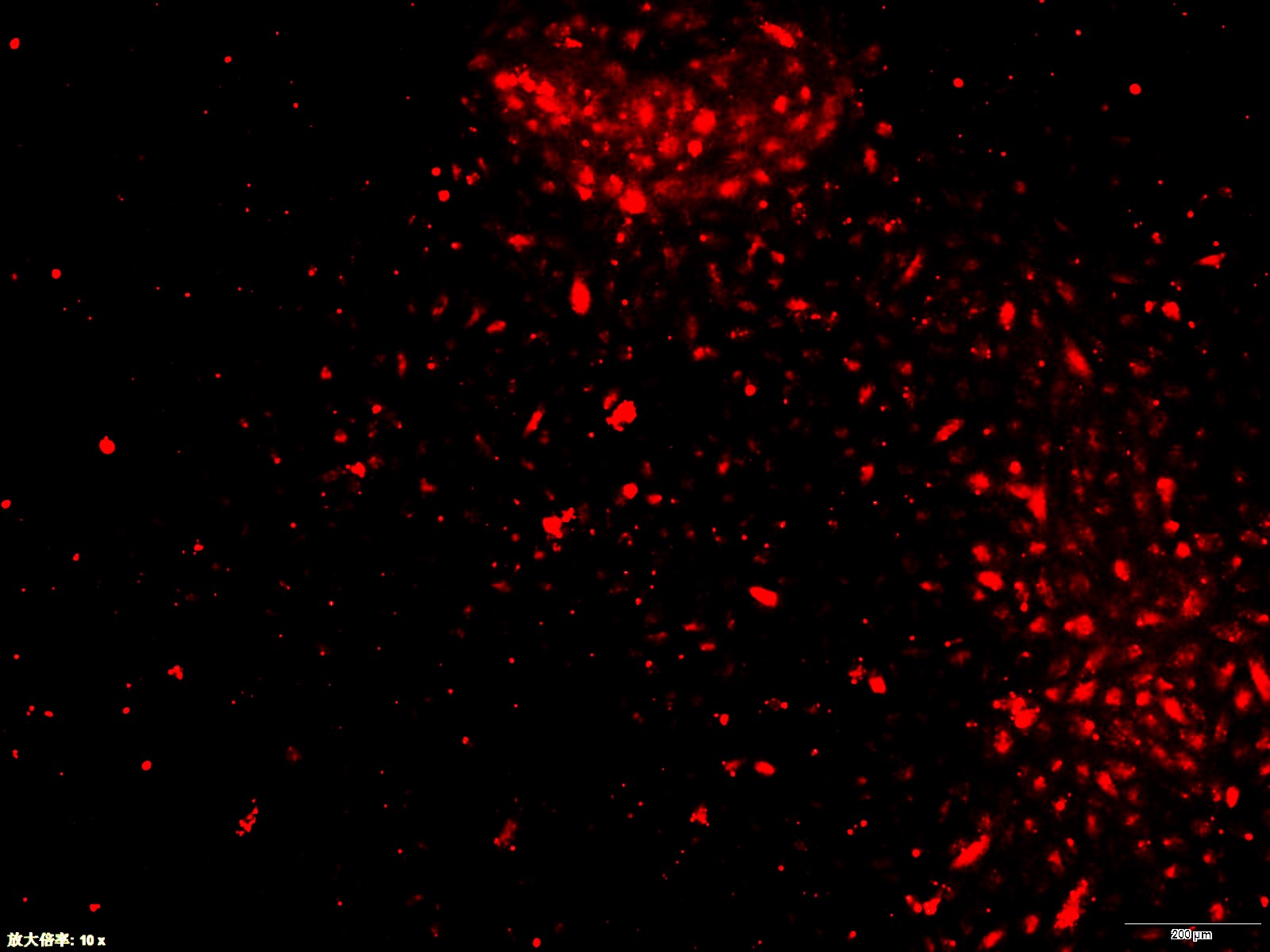

Supplement: Supplementary file 5 [file Data_Sheet_3.ZIP › cellular uptake-Vero/S1-Trimer/T.jpg]
