# Supplementary material for: Spike 1 trimer, a nanoparticle vaccine against porcine epidemic diarrhea virus induces protective immunity challenge in piglets
Source: Front Microbiol. 2024 Apr 8;15:1386136. doi: 10.3389/fmicb.2024.1386136 (PMC11033347; doi:10.3389/fmicb.2024.1386136)

Figure1. C

M COLIA1 RBD COE S1

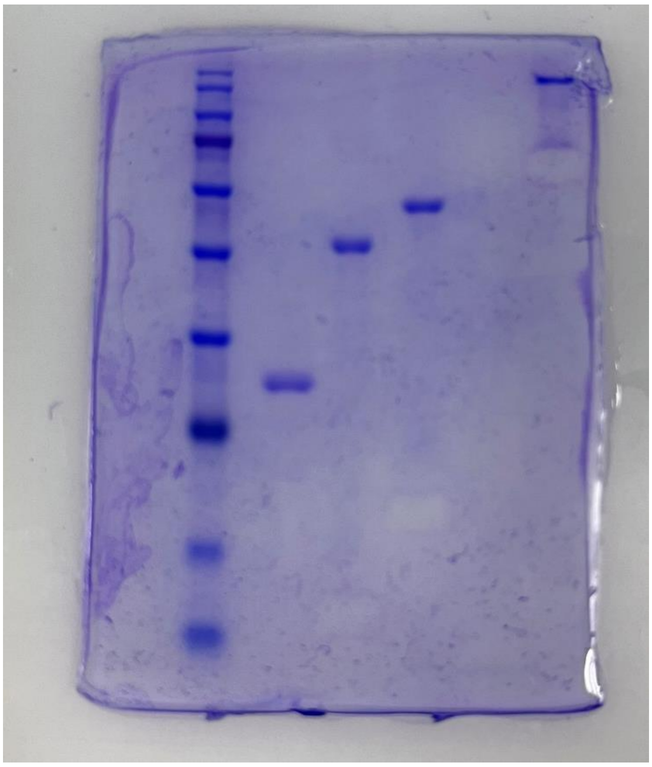

Figure 3. A

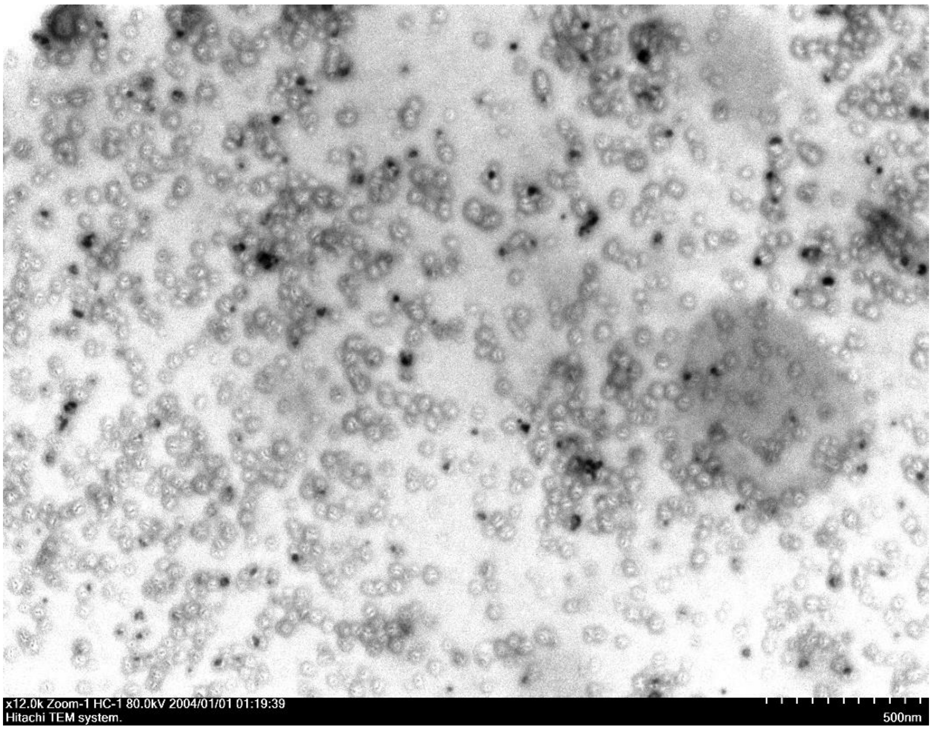

COLIA-Trimer

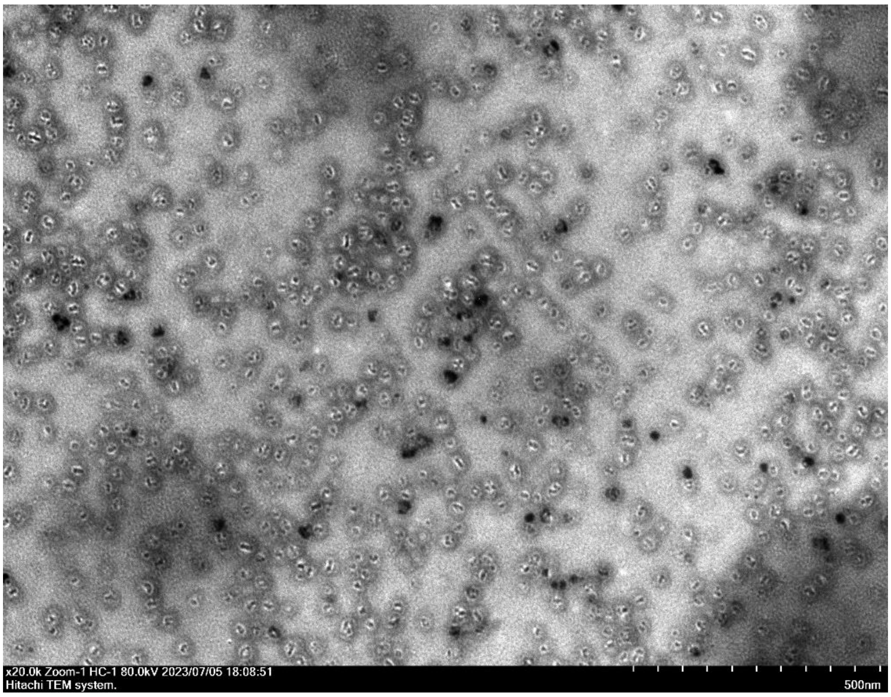

RBD-Trimer

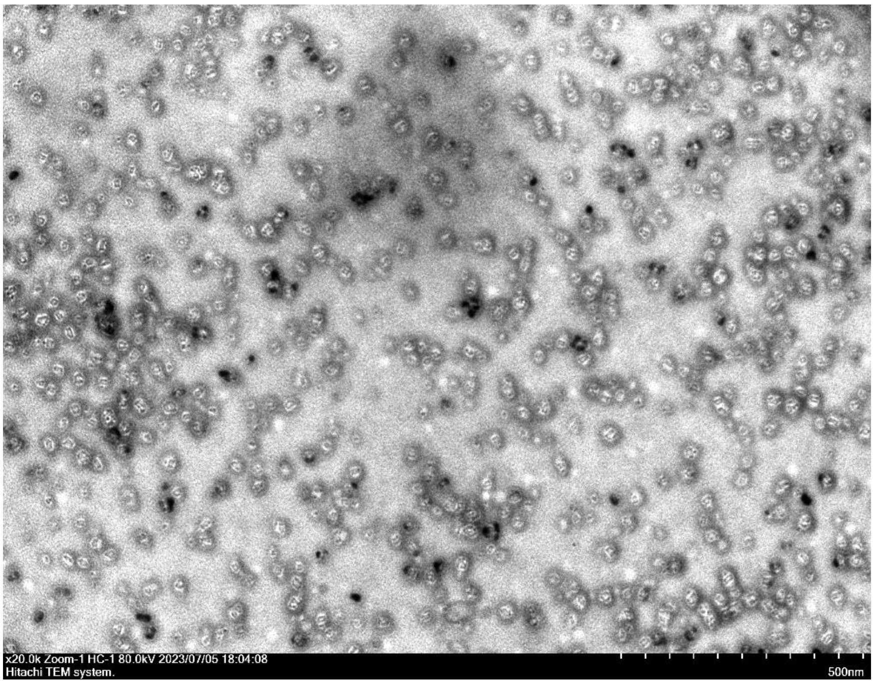

COE-Trimer

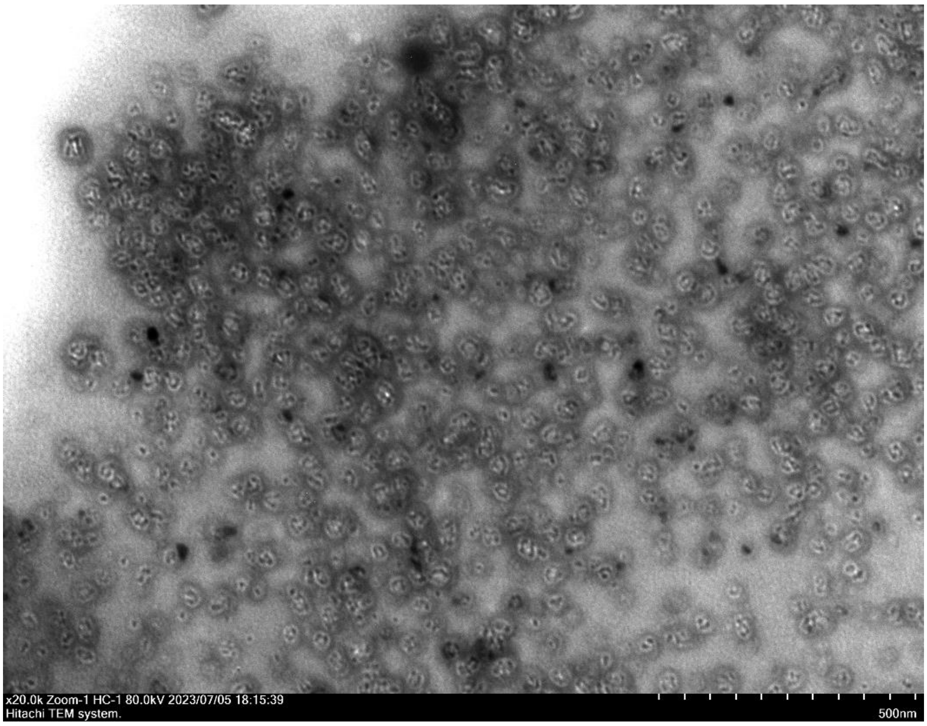

S1-Trimer

Figure 4.E

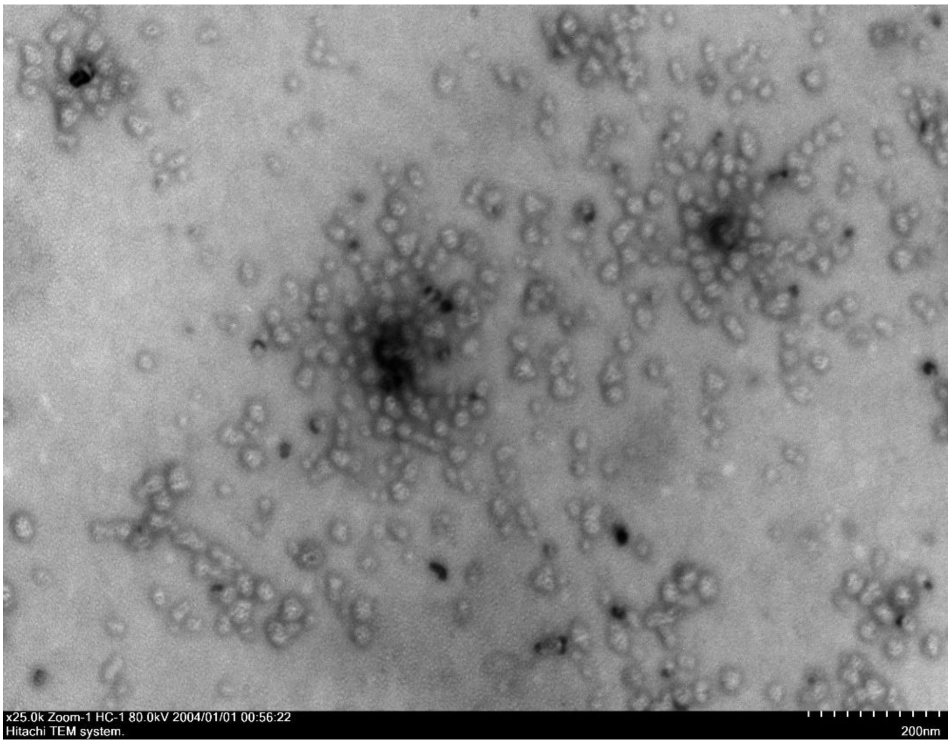

RBD-Trimer (0W)

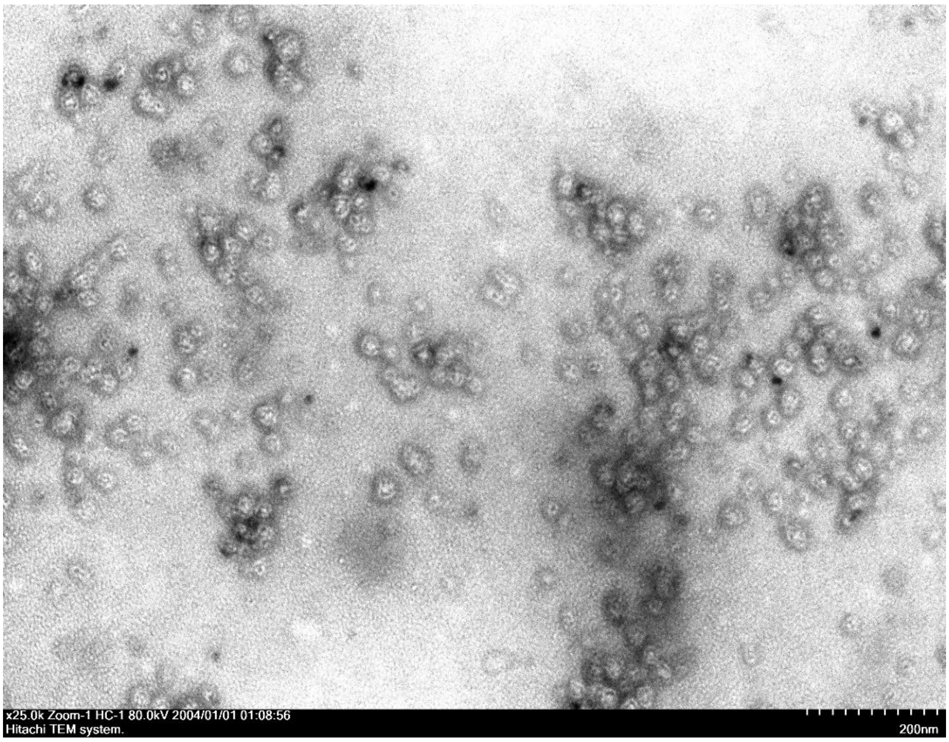

RBD-Trimer (3W)

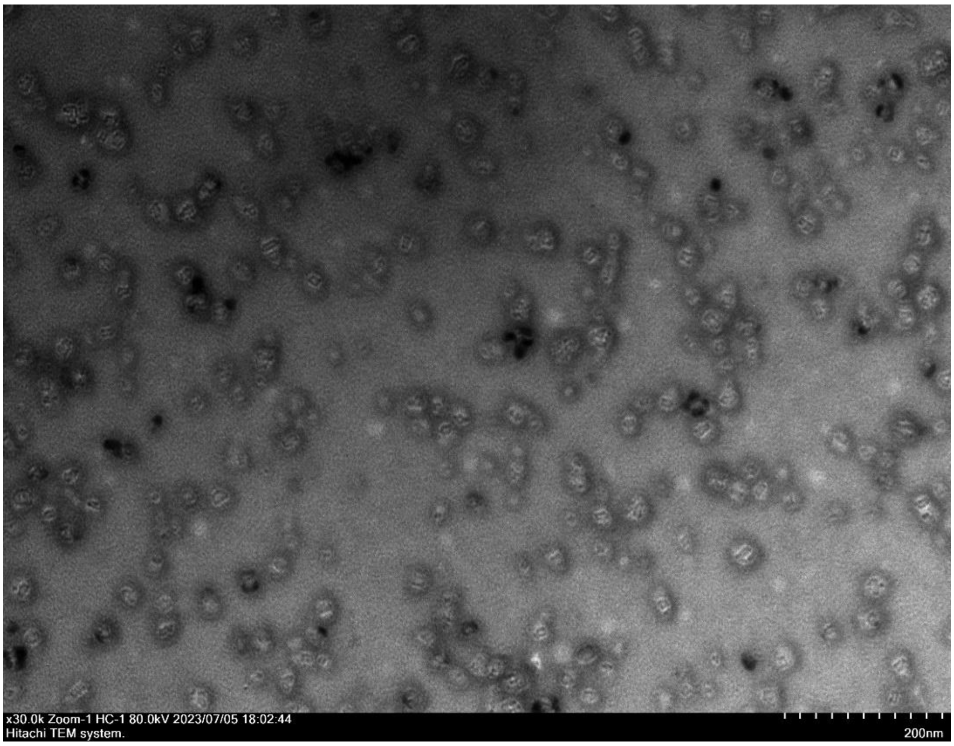

RBD-Trimer (6W)

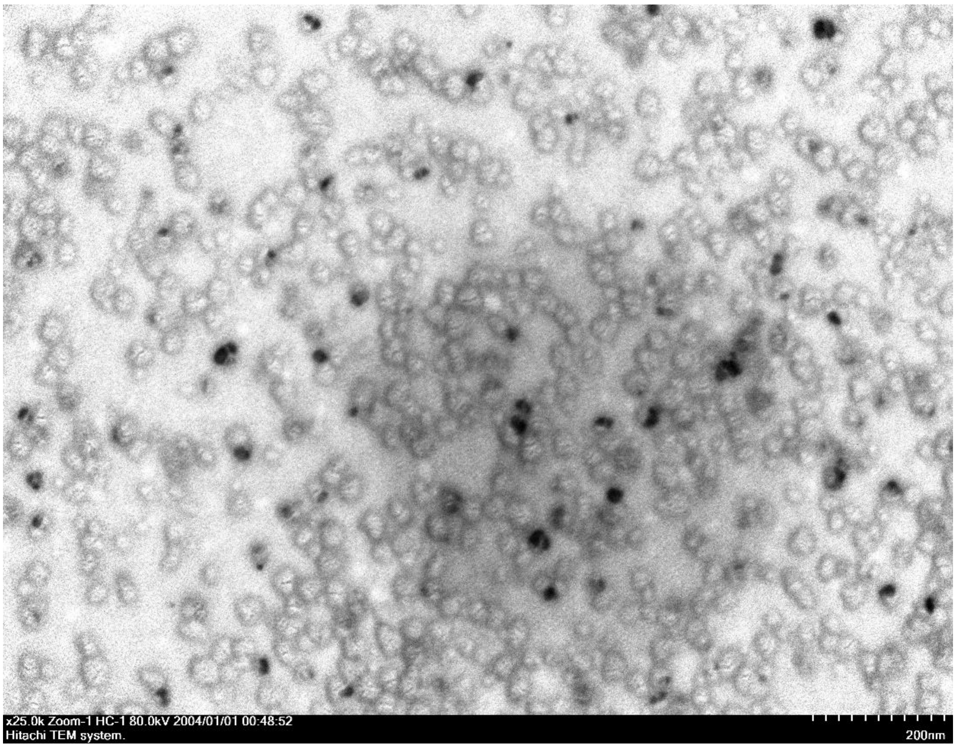

COE-Trimer (0w)

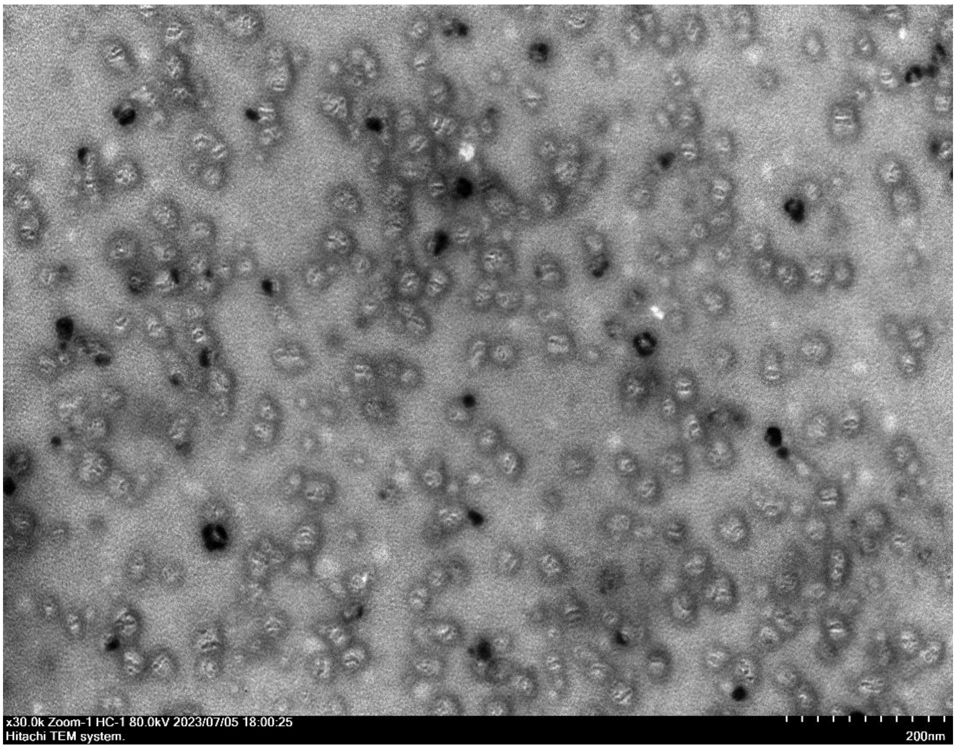

COE-Trimer (3W)

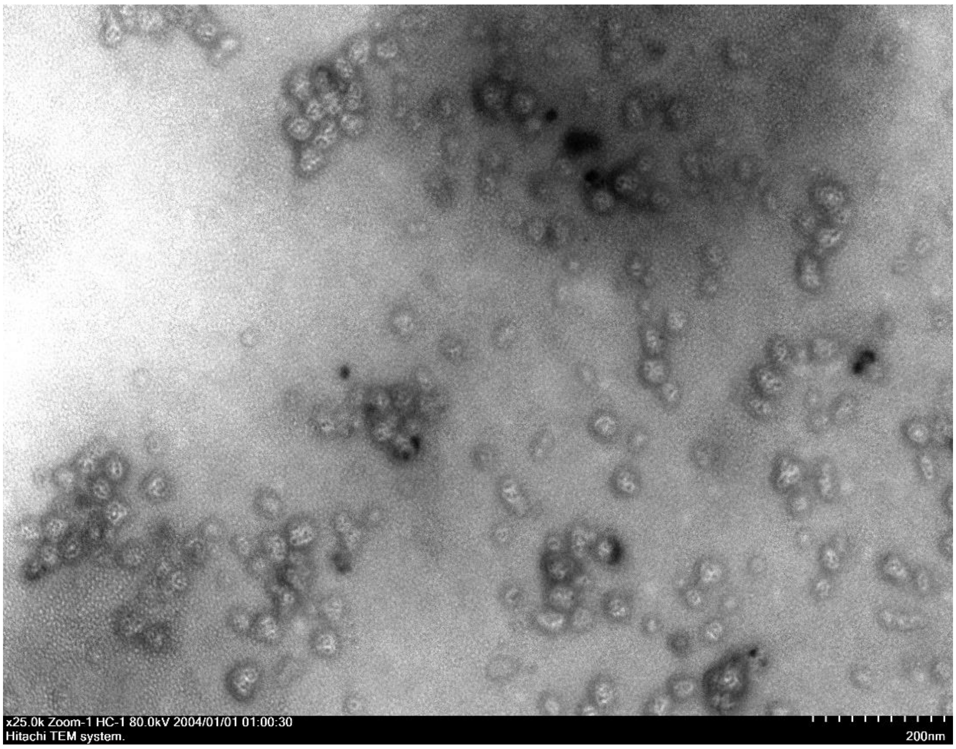

COE-Trimer (6W)

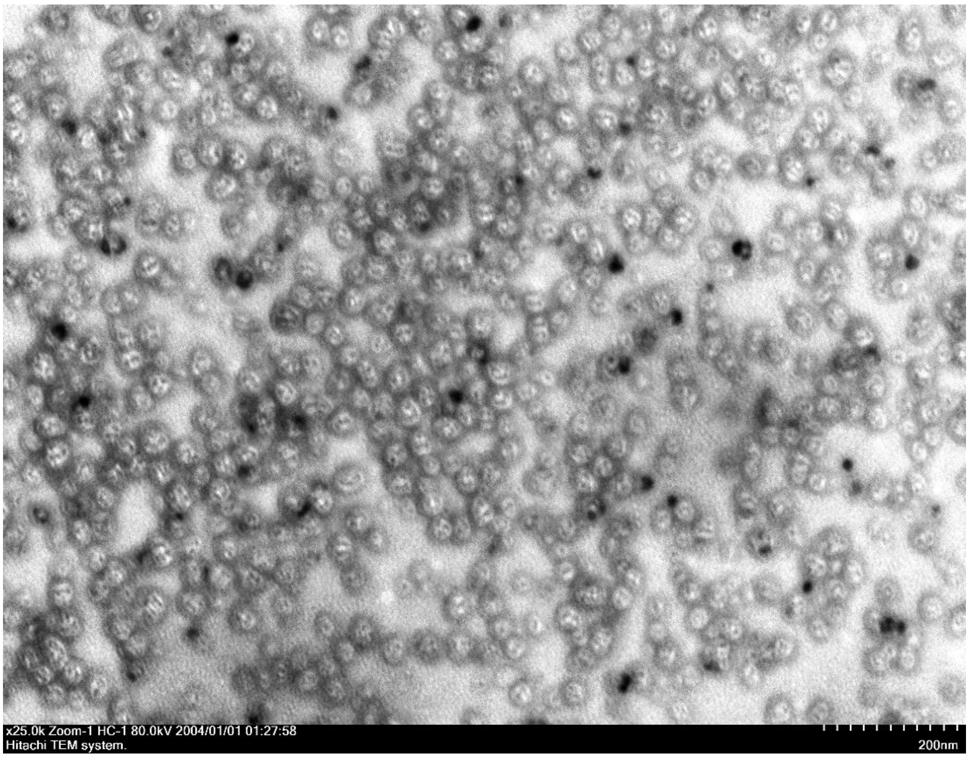

S1-Trimer (0w)

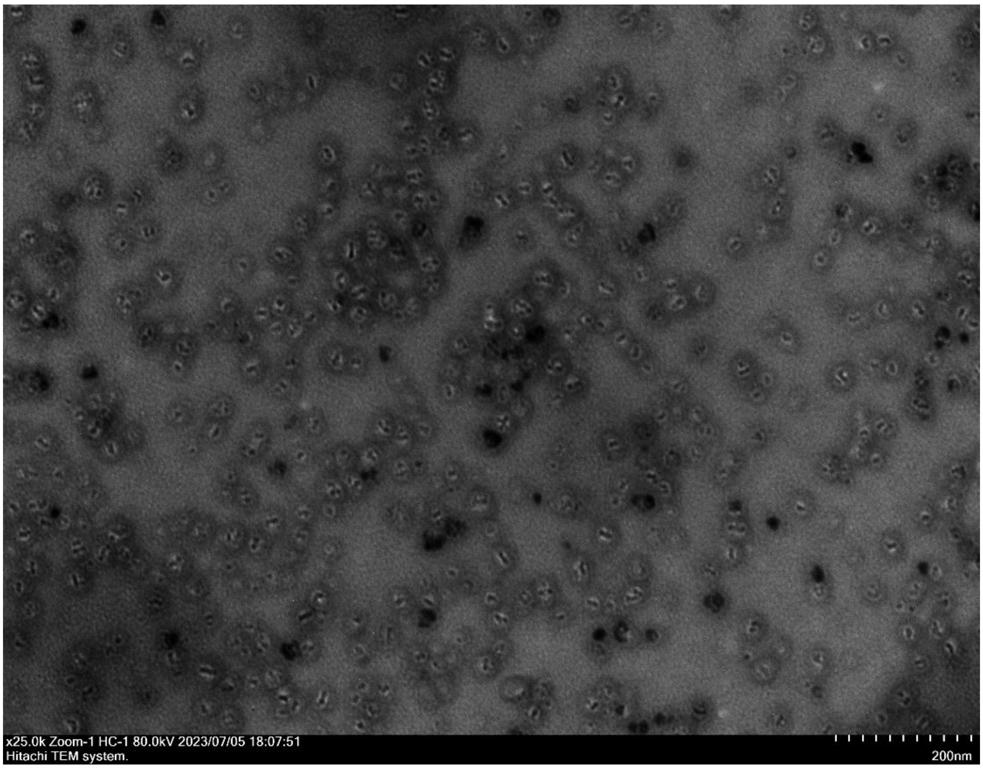

S1-Trimer (3w)

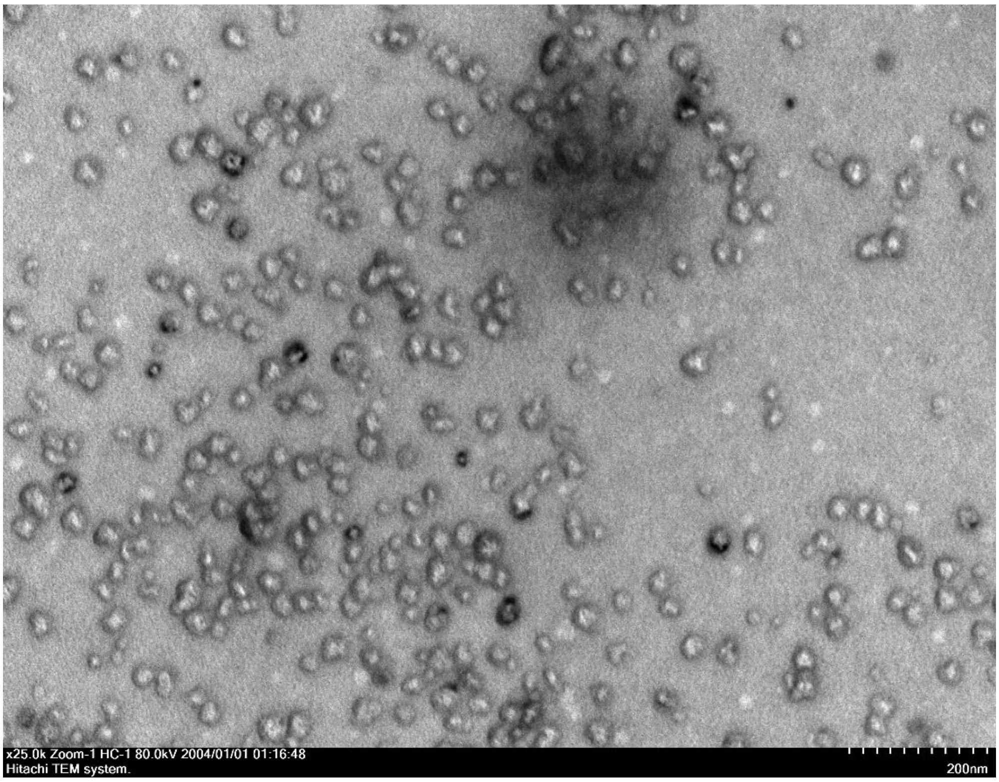

S1-Trimer (6w)

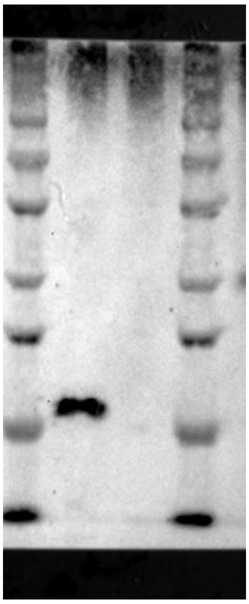
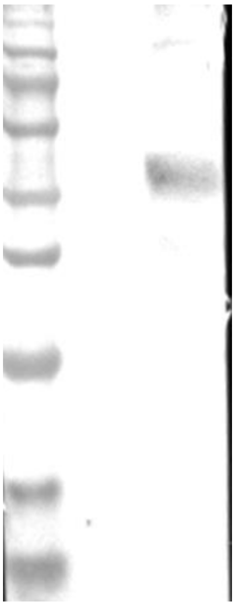
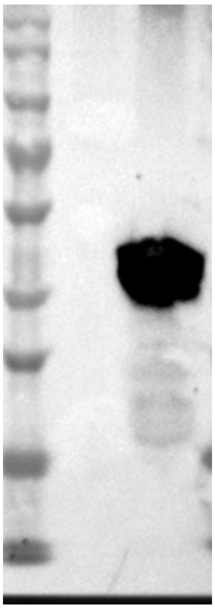
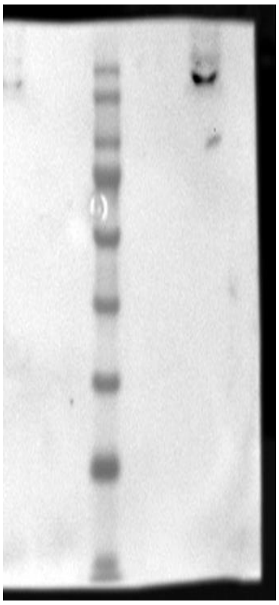

COLIA1 RBD COE S1

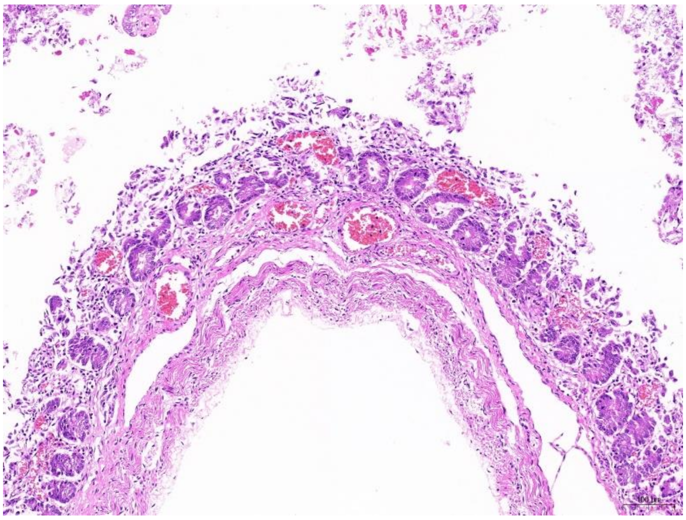

PBS(HE)

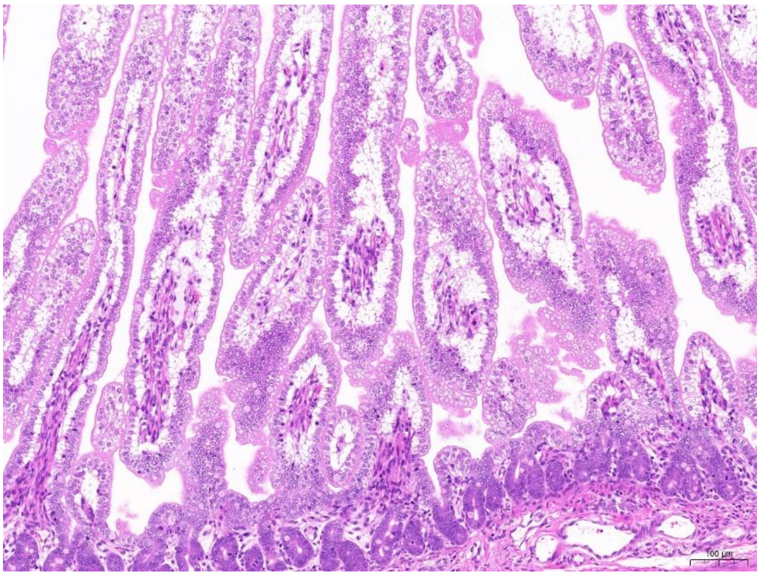

S1-Trimer (HE)

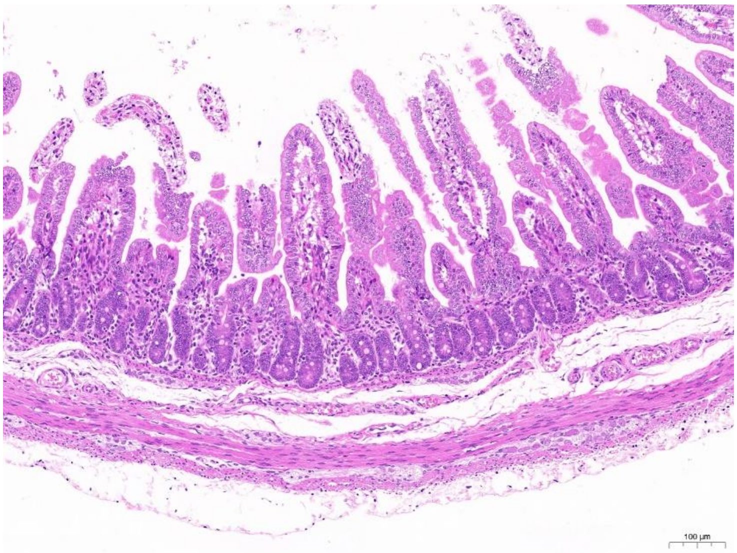

Control (HE)

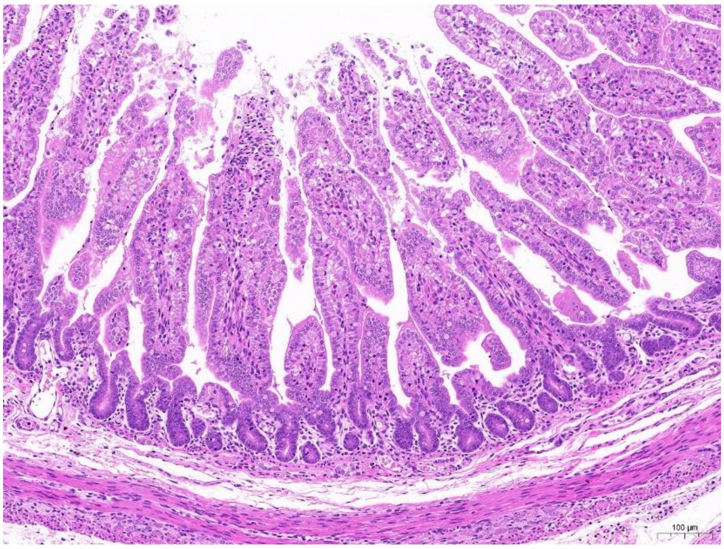

Inactivation PEDV (HE)

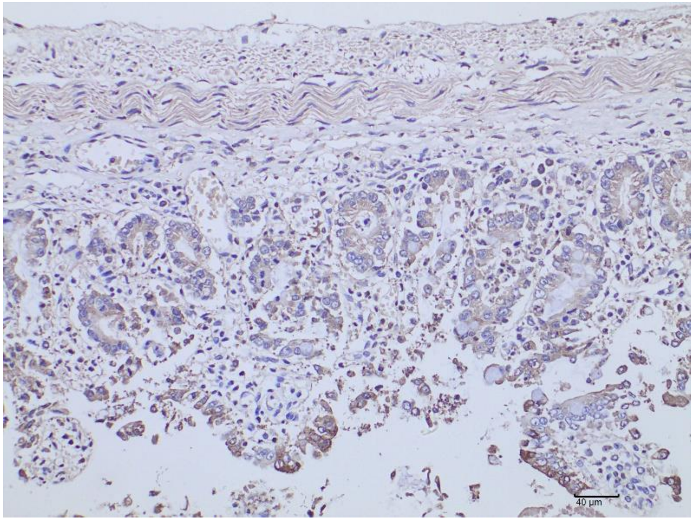

PBS (IHC)

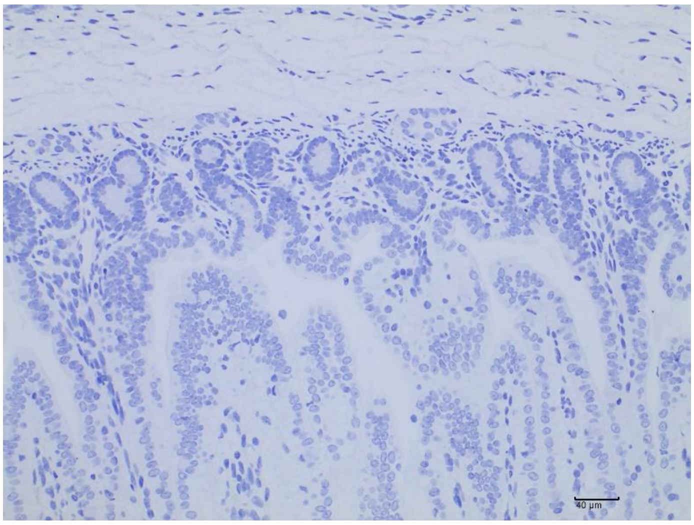

Inactivation PEDV (IHC)

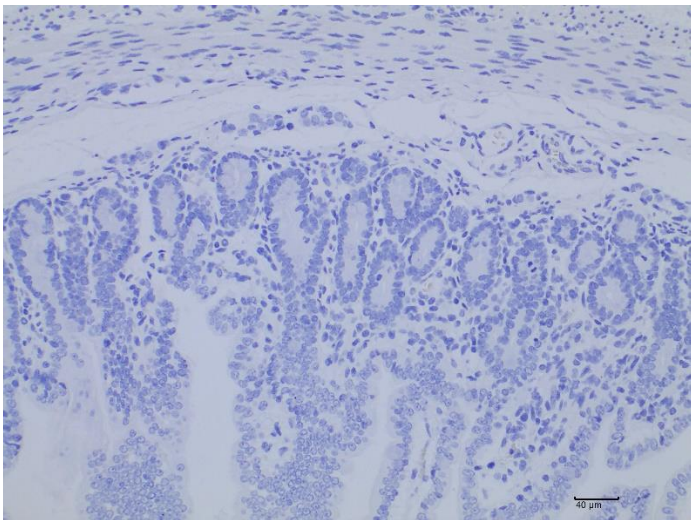

Control (IHC)

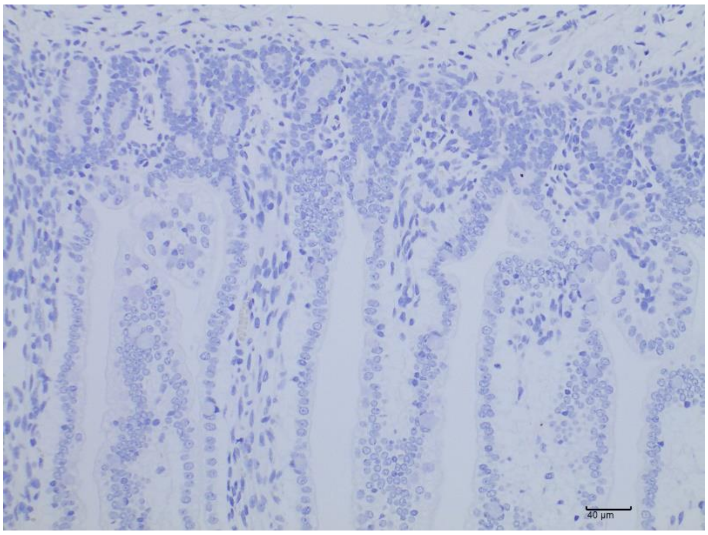

S1-Trimer (IHC)

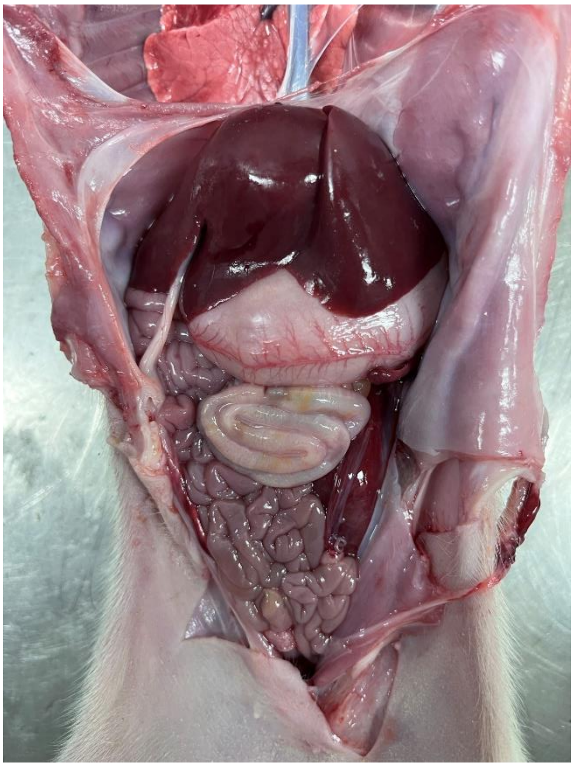

Control

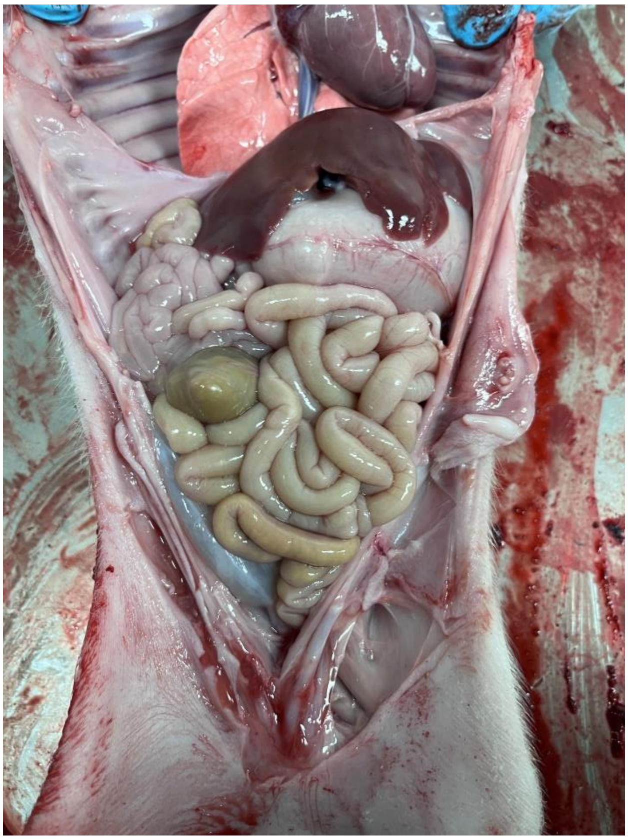

Inactivation PEDV

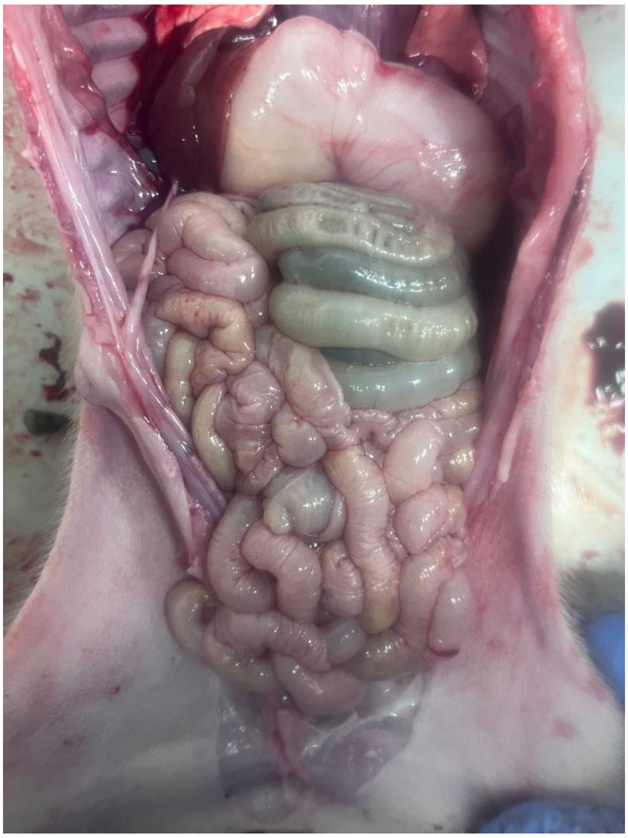

S1-Trimer

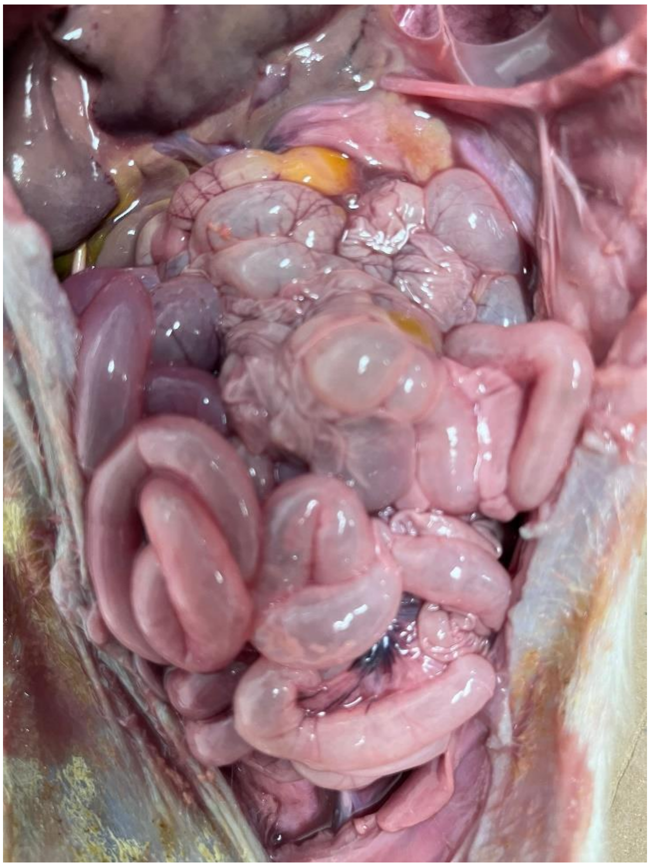

PBS

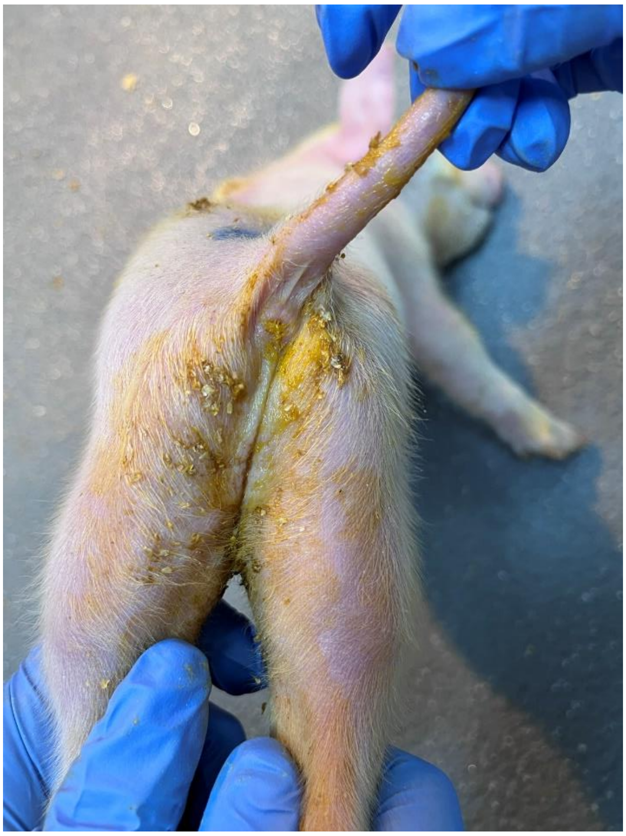

PBS

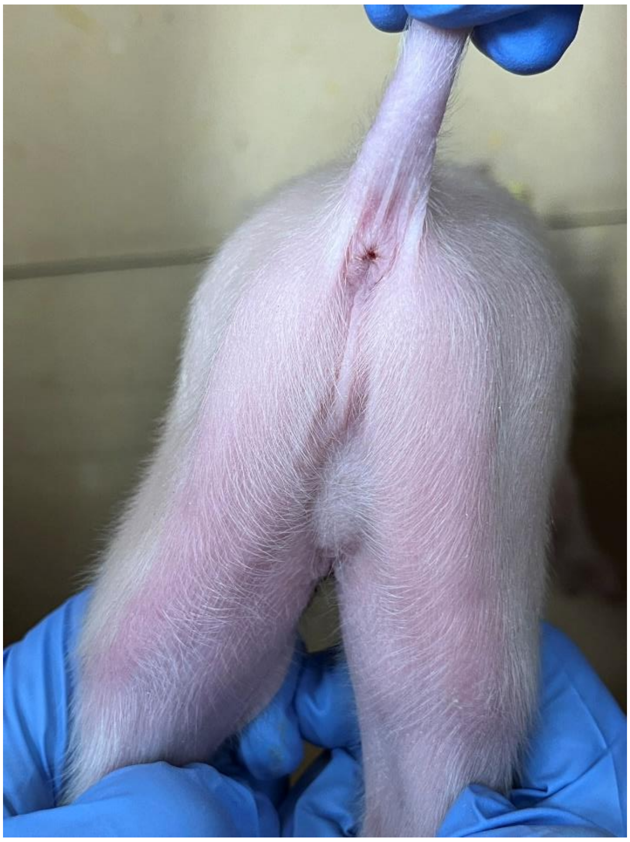

Inactivation PEDV

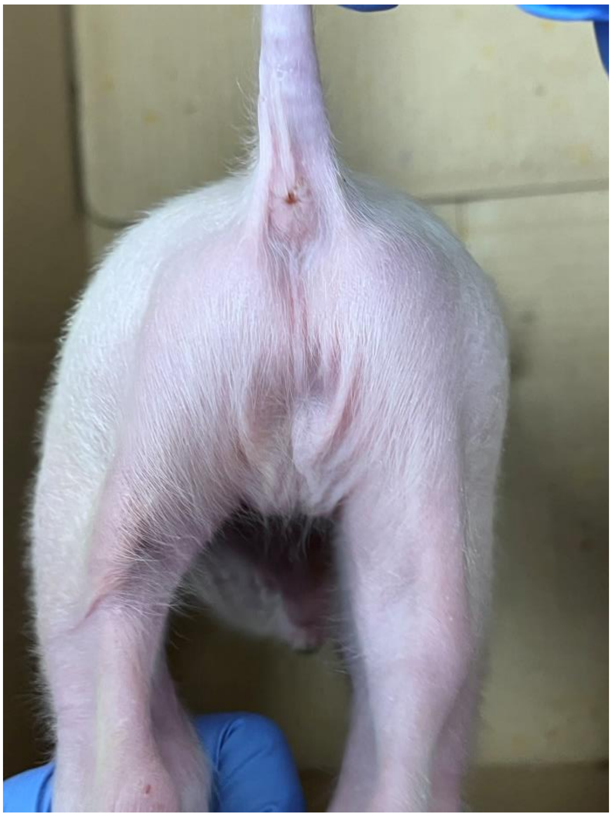

S1-trimer

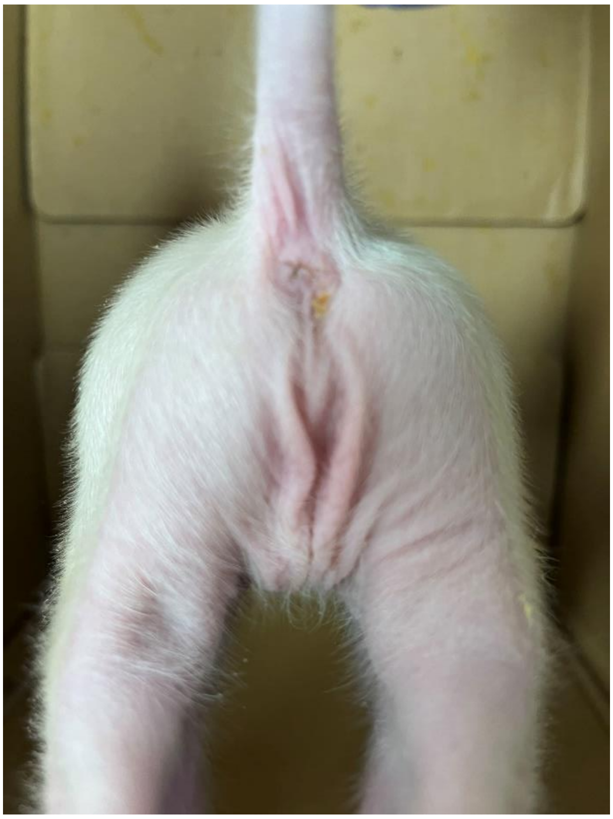

Control

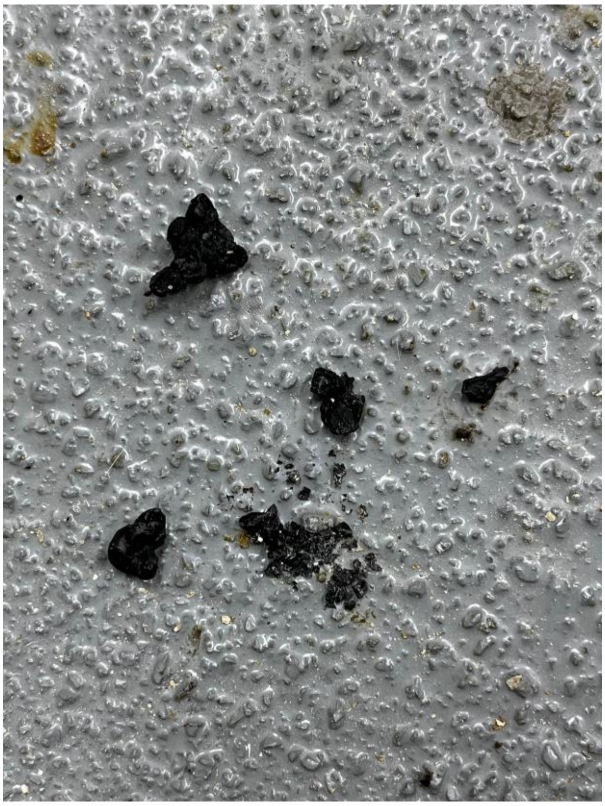

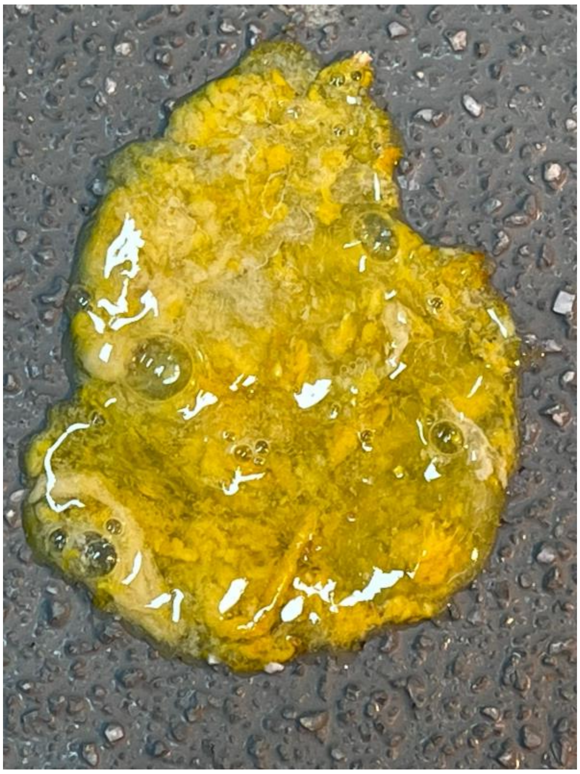

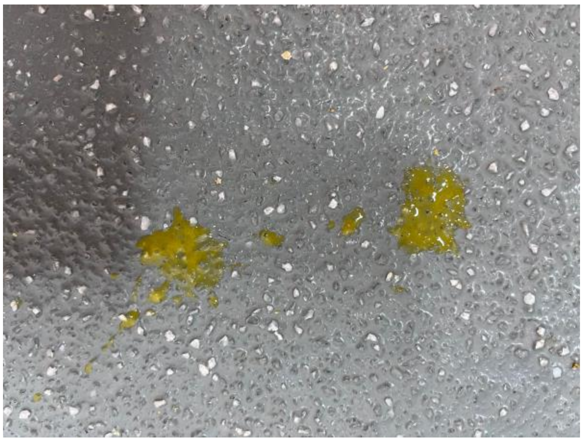

Supplement: Supplementary file 7 [file Data_Sheet_5.PDF]
