# Supplementary material for: Spike 1 trimer, a nanoparticle vaccine against porcine epidemic diarrhea virus induces protective immunity challenge in piglets
Source: Front Microbiol. 2024 Apr 8;15:1386136. doi: 10.3389/fmicb.2024.1386136 (PMC11033347; doi:10.3389/fmicb.2024.1386136)
Supplement: Supplementary file 14 [file Data_Sheet_12.PDF]

Supplementary materials

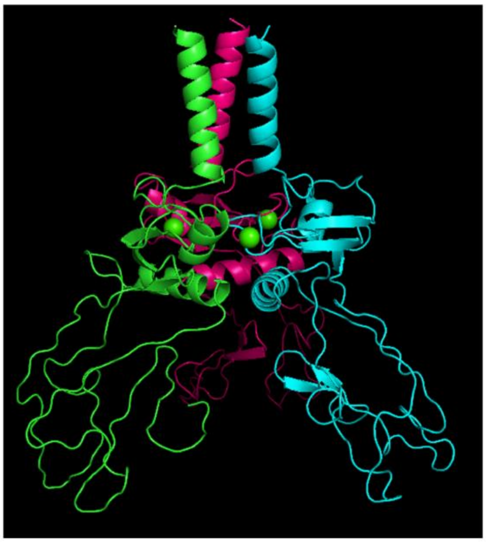

FIGURE S1 The cartoon model of COLIA1-Trimer. Green, blue and pink represented different monomers.

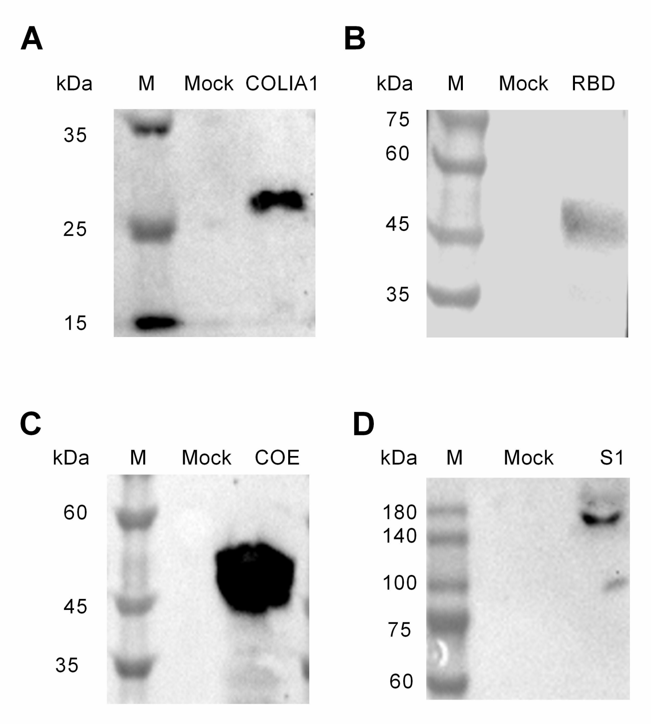

FIGURE S2 The expression of (A) COLIA1, (B) RBD, (C) COE and (D) S1.

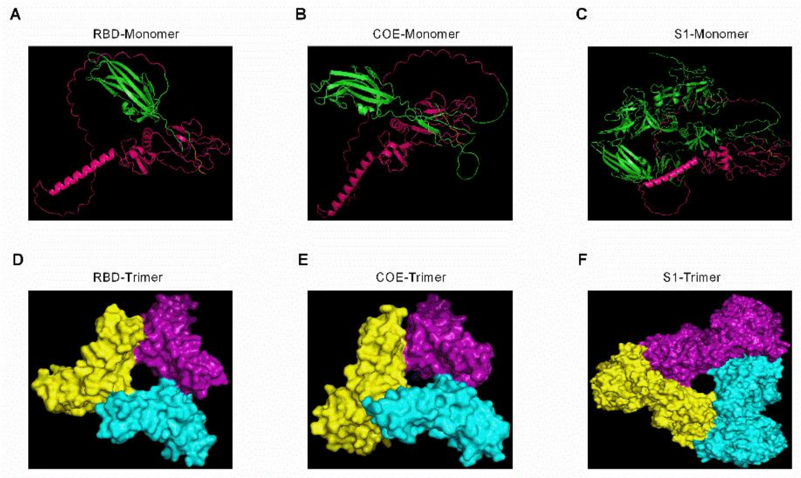

FIGURE S3 3D structures of NPs. (A-C) The cartoon model of RBD-Monomer, COE-Monomer and S1-Monomer.The representation of PEDV antigens and Trimer-Tag were green and pink, respectively. (D-F) The atomic model of RBD-Trimer, COE-Trimer and S1-Trimer.yellow, blue, and pink represented different monomers.

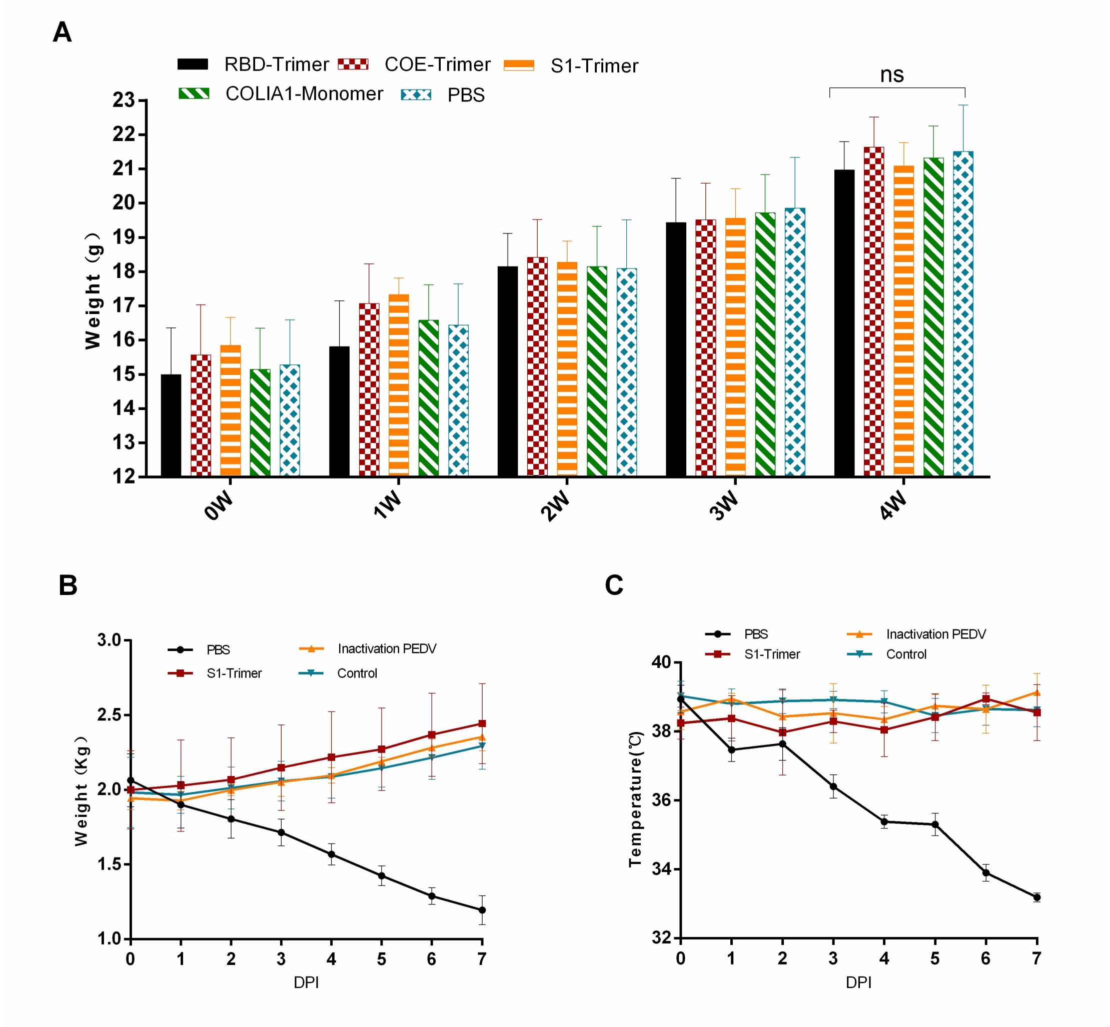

FIGURE S4 Body weight and temperature of mouse and piglet. (A) Body weight of

mouse. (B) Body weight of piglet. (C) The temperature of piglet.

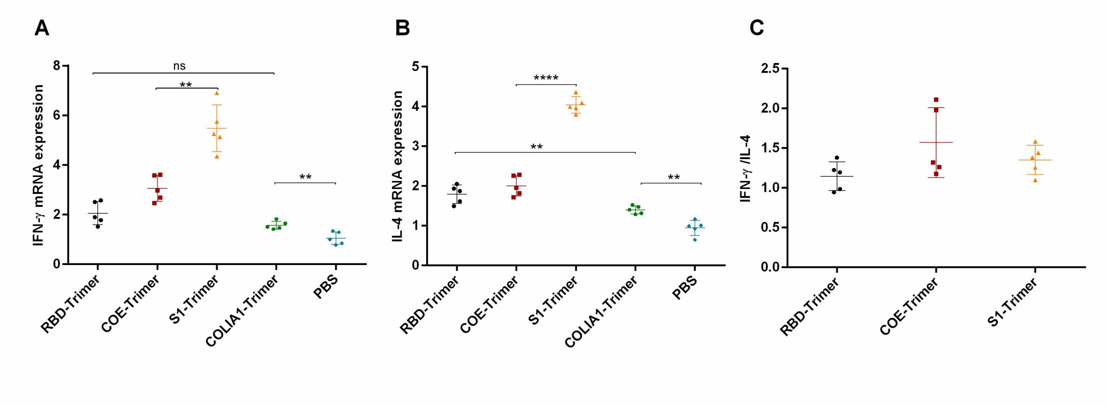

FIGURE S5 IFN-γ and IL-4 mRNA expression in spleen cells. (A) The mRNA expression of IFN-γ and (B) IL-4 in spleen cells. (C) The ratio of IFN-γ/IL-4. All error bars are expressed as ±SD. **P* < 0.05, ***P* < 0.01, ****P* < 0.001, and *****P* < 0.0001. ns, not significant. (All groups, n=5)

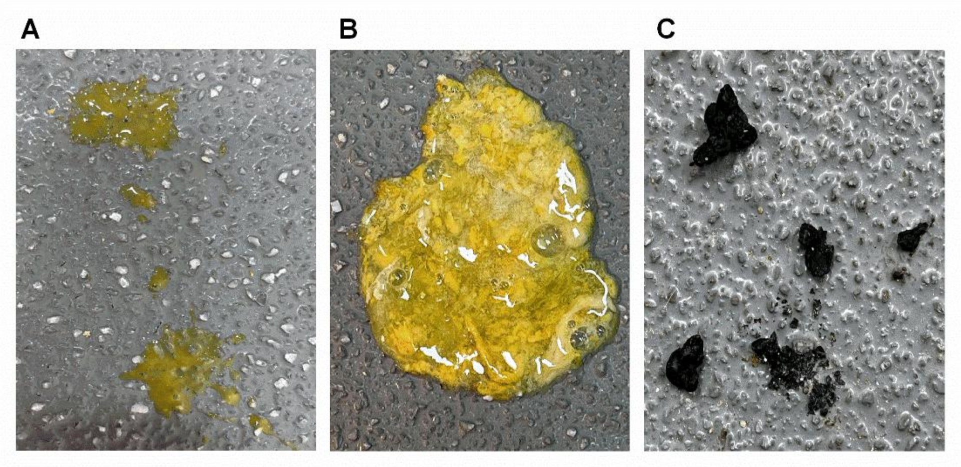

FIGURE S6 piglets clinical symptoms (A) Yellow watery feces. (B) The vomits of piglets. (C) Normal feces.

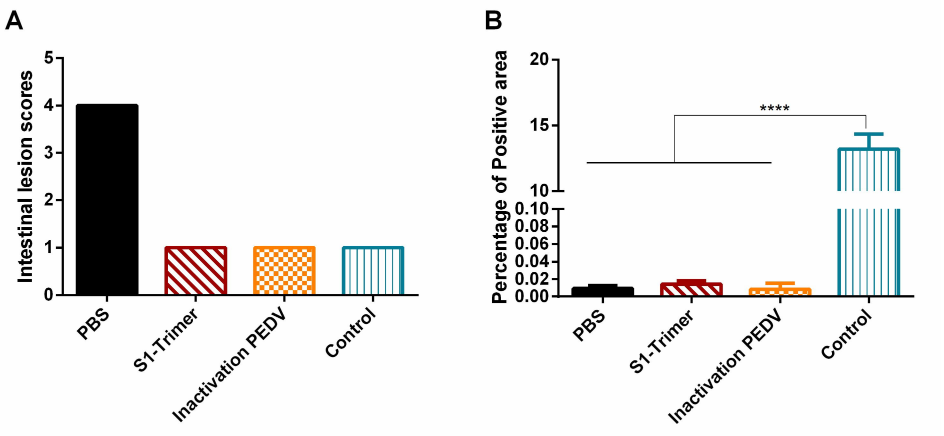

FIGURE S7 Intestinal histopathological lesion scores. (A) Intestinal histopathological

lesion scores. (B) The percentage of positive area detected by immunohistochemistry.
